# Extracellular Vesicles and Hydrogels: An Innovative Approach to Tissue Regeneration

**DOI:** 10.1021/acsomega.3c08280

**Published:** 2024-01-31

**Authors:** Amir Hashemi, Masoumeh Ezati, Minoo Partovi Nasr, Inna Zumberg, Valentine Provaznik

**Affiliations:** † Department of Biomedical Engineering, Faculty of Electrical Engineering and Communication, Brno University of Technology, Technicka 3082/12, 61600 Brno, Czech Republic

## Abstract

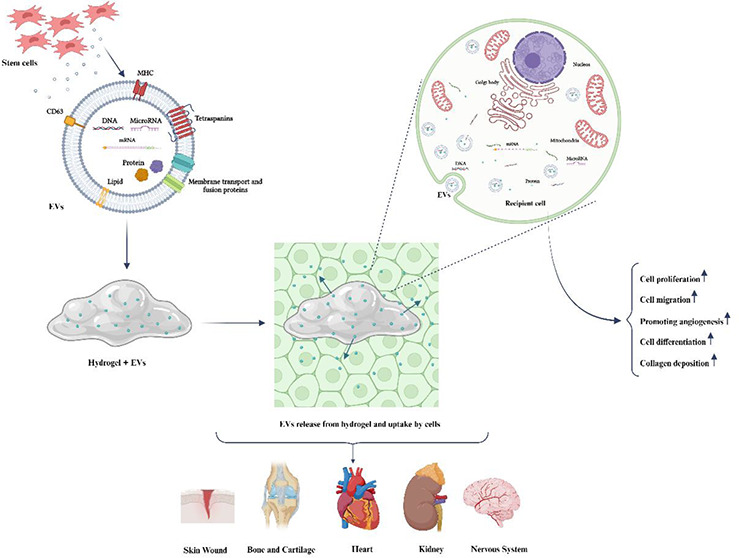

Extracellular vesicles have emerged as promising tools in regenerative medicine due to their inherent ability to facilitate intercellular communication and modulate cellular functions. These nanosized vesicles transport bioactive molecules, such as proteins, lipids, and nucleic acids, which can affect the behavior of recipient cells and promote tissue regeneration. However, the therapeutic application of these vesicles is frequently constrained by their rapid clearance from the body and inability to maintain a sustained presence at the injury site. In order to overcome these obstacles, hydrogels have been used as extracellular vesicle delivery vehicles, providing a localized and controlled release system that improves their therapeutic efficacy. This Review will examine the role of extracellular vesicle-loaded hydrogels in tissue regeneration, discussing potential applications, current challenges, and future directions. We will investigate the origins, composition, and characterization techniques of extracellular vesicles, focusing on recent advances in exosome profiling and the role of machine learning in this field. In addition, we will investigate the properties of hydrogels that make them ideal extracellular vesicle carriers. Recent studies utilizing this combination for tissue regeneration will be highlighted, providing a comprehensive overview of the current research landscape and potential future directions.

## 1 Introduction

Extracellular vesicles (EVs) include exosomes, microvesicles (MVs), and apoptotic bodies (ABs), among other cell-derived membrane structures. These vesicles, found in various body fluids, have been identified as key mediators of intercellular communication, facilitating the transfer of bioactive molecules such as proteins, lipids, and nucleic acids.^1^ This ability to transport biological information has placed EVs at the forefront of numerous fields of biomedical research, including cancer biology, immunology, and regenerative medicine.^2^ EVs are classified into various categories according to their dimensions, biogenesis, and pathways of release. Out of these options, exosomes are frequently emphasized because of their small size, with a typical diameter of 30–150 nm and distinct process of formation. However, within numerous scientific frameworks, the term ‘exosome’ is commonly employed interchangeably with small EVs (sEVs). The use of these terms interchangeably is a result of the practical constraints of existing isolation techniques and recognizes the shared characteristics among various subtypes of EVs. Therefore, it is crucial to approach the term ’exosome’ with a comprehension of these intricacies and the developing field of EVs research.^3^

Multivesicular bodies (MVBs) originate from the inward budding of the endosomal membrane. Exosomes are released into the external environment when MVBs merge with the plasma membrane.^4^ The biogenesis of exosomes involves diverse cellular machinery, such as endosomal sorting complexes required for transport (ESCRT) and lipid-based mechanisms and is controlled by various factors.^5^ This complex process ensures the selective packaging of bioactive molecules such as proteins, lipids, and nucleic acids, such as mRNAs, microRNAs (miRNA), and other noncoding RNAs, into exosomes.^6^ Alternatively, some MVBs undergo degradation either by directly fusing with lysosomes or by merging with autophagosomes and subsequently fusing with lysosomes.^7^ MVs, or ectosomes, are EVs that have a size vary between 50 and 2000 nm. They are produced and released by the plasma membrane through outward budding.^8^ ABs are generated when cells undergo blebbing during the process of apoptosis. Typically, these entities are larger, ranging in size from 50 to 5000 nm,^9^ which falls toward the upper limit of the EVs size range.^10^

The composition of EVs is highly diverse and dependent on cell type. They transport proteins, lipids, and nucleic acids that can be transferred to recipient cells and used to modify their functions. The lipid bilayer of EVs protects this cargo from enzymatic degradation in the extracellular environment, allowing for long-distance communication between cells.^11^ This exceptional capacity of EVs to transport and deliver bioactive molecules has led to their investigation as potential therapeutic agents in fields such as regenerative medicine. sEVs derived from mesenchymal stem cells (MSCs-sEVs) have shown promise in regenerative medicine. These sEVs are loaded with regenerative molecules, such as growth factors, cytokines, and miRNAs, which can promote cell proliferation, angiogenesis, and immunomodulation, promoting tissue repair and regeneration.^12^ However, the therapeutic application of sEVs is frequently hampered by delivery difficulties. sEVs are rapidly cleared from the body due to their small size and susceptibility to degradation, limiting their ability to maintain a sustained presence at the injury site. In addition, the lack of targeting capabilities can make it challenging to deliver exosomes to particular tissues or cells.^13^

To overcome these obstacles, researchers have turned to biomaterials, specifically hydrogels, as sEVs delivery vehicles. Hydrogels are three-dimensional (3D) hydrophilic polymer networks that can absorb large quantities of water, making them highly biocompatible and appropriate for biological applications.^14^ Their porous structure permits exosome encapsulation, providing a localized and controlled release system that can improve the therapeutic efficacy of sEVs.^15^ In addition, the physical properties of hydrogels, such as their mechanical strength, degradation rate, and porosity, can be tailored to meet the specific requirements of the application, making hydrogels a versatile platform for sEVs delivery.^16^

This article aims to provide a comprehensive overview of the role of EVs-loaded hydrogels in tissue regeneration. We will investigate the properties, functions, and methods of EVs characterization. This paper differs from previous reviews that mainly focused on the combination of EVs and hydrogels. The distinctive aspect of our work is the thorough profiling of exosome, which includes in-depth profiling of nucleic acids, proteomics, and lipidomics. The central focus of our discussion is the application of machine learning (ML) in exosome profiling, which demonstrates its capacity to overcome the inherent constraints of conventional methodologies. Through the utilization of ML algorithms, we greatly improve the accuracy and precision of EVs classification, which is a crucial development for both diagnostic and therapeutic purposes. In addition, our Review explores different cutting-edge techniques by which ML can assist in the creation of hydrogels that are specifically designed for the incorporation of EVs. ML is becoming a crucial tool that is ready to completely transform the design and functionality of hydrogels in important fields like tissue engineering and drug delivery. While the use of ML in these fields is still in its early stages, it has the potential to bring about groundbreaking discoveries and significant progress in the future.

In the following sections, we delve into the complexities of EVs formation, composition, and techniques used for characterization. We additionally examine the distinctive characteristics of hydrogels that make them optimal vehicles for EVs transport. We investigate the techniques for efficiently loading and releasing EVs in hydrogels, with a specific emphasis on their regenerative potential. Our objective is to present a thorough analysis of the current research on the combined effects of EVs and hydrogels in tissue regeneration. We will focus on recent studies and explore potential future advancements in this area.

Ultimately, our Review tackles the current obstacles and foresees upcoming patterns, providing readers with a comprehensive and perceptive comprehension of this captivating field of research. This approach not only enhances the scholarly discussion but also facilitates the development of novel therapeutic approaches in regenerative medicine, representing a noteworthy contribution to the field.

## 2 EVs: Biogenesis, Composition, and Characterization Methods

### 2.1 The Biogenesis of Exosomes

**Figure 1:**
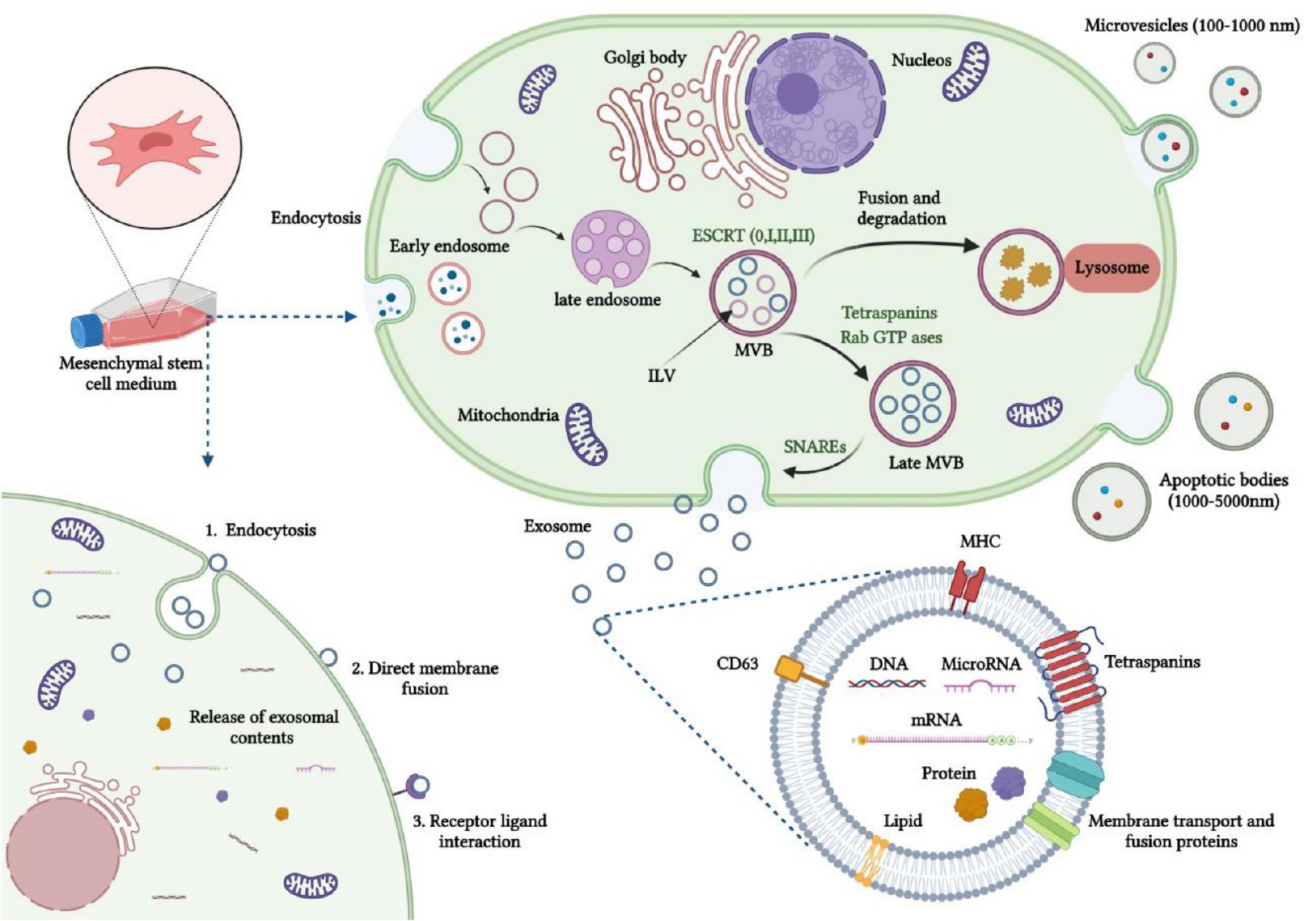
Formation, release, and absorption of MSCs-exosomes involves a specific pathway in stem cells. Endocytosis initiates the formation of early endosomes. The endosomes, along with selected cargos such as nucleic acids, proteins, and lipids, are subsequently packed into MVBs. After merging with the plasma membrane, these MVBs release exosomes into the extracellular space. The contents of these exosomes, which consist of proteins, mRNAs, miRNAs, and DNAs, are then transported to recipient cells via three primary mechanisms: direct fusion with the cell membrane, interaction with receptors, and endocytosis.

As depicted in Figure 1, exosome biogenesis is a complex process involving the transformation of early endosomes into late endosomes or MVBs. This transformation is distinguished by the inward budding and separation of the endosomal membrane, which contains specific cargo. Within the lumen of the endosomes, this results in the formation of intraluminal vesicles (ILVs) that are prepared for exocytosis. Mechanisms involved in exosome biogenesis can be grouped into two pathways: The ESCRT-dependent pathway and the ESCRT-independent pathways.^17^

#### 2.1.1 ESCRT

Approximately 30 proteins are organized into four complexes to form the ESCRT machinery: ESCRT-0, ESCRT-I, ESCRT-II, and ESCRT-III. The discovery of these complexes has shed light on the cellular mechanisms underlying the biogenesis of EVs. The formation of ILVs begins when cargo is loaded onto the limiting membrane of MVBs. ESCRT-0, a multivalent ubiquitin-binding complex composed of the Hepatocyte Growth Factor-Regulated Tyrosine Kinase Substrate (HRS) and the Signal Transducing Adapter Molecule (STAM), facilitates this process. This complex possesses ten ubiquitin-binding sites, which is essential for the capture of polyubiquitylated cargo. Once the ubiquitinated cargo binds to the ESCRT-0 complex, the HRS-STAM complex recruits the ESCRT-I complex in order to transmit cargoes via TSG-101 binding ubiquitin. The ESCRT-I complex then recruits the ESCRT-II complex via the link between the VPS28 and VPS36 subunits. The ESCRT-III complex is finally recruited by the ESCRT-II complex to nucleate charged MVB protein 2–4 (CHMP2–4) polymers, thereby completing the budding of ILVs and the fission of this membrane. Moreover, the accessory protein AAA-ATPase VPS4 is involved in the disassembly and recycling of the ESCRT-III complex.^18−20^

#### 2.1.2 Syntenin

Syntenin, syndecan, and the ESCRT accessory protein ALG-2 interacting protein X (ALIX) participate in an alternative pathway that plays a crucial role in the biogenesis of exosomes. Syntenin recruits ALIX by binding to the cytoplasmic domain of syndecan. This recruitment facilitates the inward budding of ILVs in conjunction with ESCRT-I, II, and III components. This process is dependent on Src-mediated endocytosis of syndecan-1 and requires Phospholipase D2 (PLD2) and ADP-ribosylation factor 6 (ARF6) GTPase activity. Recent research indicates that the dephosphorylation of syntenin by the Src homology 2-containing protein tyrosine phosphatase 2 (Shp2) regulates the biogenesis of exosomes. This regulation has implications for the cross-talk between epithelial cells and macrophages that is mediated by exosomes.^21^

#### 2.1.3 Lipids

Additionally, exosome formation can occur independently of ESCRTs. Multiple studies have demonstrated that MVBs continue to form even when the ESCRT-dependent pathway is maximally inhibited. Trajkovic et al. have uncovered an ESCRT-independent pathway for exosome biogenesis, demonstrating that the transfer of exosome-associated domains into the endosome lumen is dependent on the sphingolipid ceramide in mouse oligodendroglial cells. Ceramide has the ability to promote domain-induced budding by inducing the coalescence of small microdomains into larger ones. In addition, the tapering structure of ceramides can result in spontaneous negative curvature by generating area differences between membrane leaflets. Cholesterol, an essential component of MVBs, is an additional lipid that is abundant in exosome membranes. The accumulation of cholesterol in the late endosomal compartments of oligodendroglial cells has been shown to increase the flotillin-dependent secretion of exosomes.^7,22,23^

#### 2.1.4 Tetraspanins

In addition to lipids, proteins from the tetraspanin family play a significant role in the regulation of cargo sorted for exosome secretion. This family, which is distinguished by four transmembrane domains, has as many as thirty-three members in mammals, including CD9, CD37, CD51, CD53, CD63, CD81, and CD82. Initial research on the tetraspanin-dependent mechanism of cargo sorting to ILVs focused on CD63, an endosomal sorting protein in melanoma cells. CD63 also played a role in targeting cargo to exosomes secreted by melanoma cells and in the biogenesis of exosomes in fibroblasts from Down syndrome patients, according to additional research. CD81, a member of the tetraspanin family that is abundant in exosomes, has been demonstrated to target its ligands array into secreted exosomes in mouse lymphoblasts. It has been reported that the expression of CD9 and CD82 enhances the exosomal release of β-catenin from HEK293 cells. Notably, the tetraspanin Tspan8 contributes to exosome biogenesis in rat adenocarcinoma cells by selectively recruiting proteins and mRNA into exosomes without affecting the amount of secreted exosomes.^24−27^

#### 2.1.5 Other Mechanisms Involved in the Exosomes Biogenesis

MVBs involve additional mechanisms in their biogenesis. For instance, ILVs-isolated soluble proteins are dependent on the chaperones HSC70. This chaperone recruits the transferrin receptor (TFR) to exosomes and binds cytosolic proteins with the KFERQ motif, resulting in their selective transfer to ILVs in reticulocytes.^28,29^

Exosomes transport nucleic acids, including DNA and RNA sequences.^30^ It was revealed that the proportion of miRNAs in exosomes is greater than in their donor cells.^31^ Interestingly, miRNAs are not loaded into exosomes at random.^32^ The transfer of miRNAs into exosomes can occur via several pathways, including the SUMOylated heterogeneous nuclear ribonucleoprotein A2B1 (hnRNPA2B1), the neural sphingomyelinase 2 (nSMase2) -dependent pathway, the 3′-end of the miRNA sequence-dependent pathway, and the miRNA induced silencing complex (miRISC) -related pathway^33−36^

It has also been suggested that retroviral coat proteins such as Gag (and their silent copies present in animal genomes) can facilitate the packaging of RNA into extracellular vesicles in Drosophila. These proteins effectively target the RNA they recognize to the plasma membrane or MVB membrane, resulting in virus-like particle release.^37,38^ Post-translational modifications (PTMs) can also direct cargo into exosomes. Other modifications of proteins, such as SUMOylation, ISGylation, phosphorylation, glycosylation, and acetylation, can also result in their sorting into exosomes,^39,40^ in addition to the ubiquitin domain of ubiquitinated proteins being recognized by the ESCRT protein TSG101. ISGylation of TSG101, for instance, induces its aggregation and degradation, thereby inhibiting exosome secretion.^41^ The interaction between ESCRT and phosphoinositols^42^ can sort SUMOylation of -synuclein, the major protein of pathological aggregates in Parkinson’s Disease, into MVB.

In conclusion, the process of exosome biogenesis is complex and heavily dependent on the cargo that affects the function of exosomes. It is important to note that different mechanisms operate simultaneously within a single cell, resulting in the presence of multiple subtypes of MVBs. During the maturation of MVBs, multiple mechanisms act simultaneously or sequentially to facilitate the release of various exosomes.^43^

### 2.2 Biogenesis of MVs

MVs and exosomes follow different pathways in the process of extracellular vesicle biogenesis. MVs are formed by the direct outward budding and fission of the plasma membrane, which is a fundamentally different process from the endosomal origin of exosomes. MVs are generally larger than exosomes, have a size range of 50 to 2000 nm. However, there is some size overlap between these two types of vesicles. The generation of MVs is an intricate process that involves the redistribution of phospholipids and the contraction of cytoskeletal proteins.^44^ The distribution of phospholipids in the plasma membrane is asymmetrical and rigorously controlled by aminophospholipid translocases, such as flippases and floppases. The function of these translocases is to facilitate the movement of phospholipids between the inner and outer leaflets of the plasma membrane. An essential process in MVs formation involves the movement of phosphatidylserine to the outer layer of the membrane, followed by the contraction of cytoskeletal structures, specifically through interactions between actin and myosin. Studies conducted on melanoma models have demonstrated that the upregulation of ARF6, a GTP-binding protein, results in an augmentation of MVs excretion.^45^ This process entails a series of signals that triggers the activation of phospholipase D, leading to the phosphorylation and activation of the myosin light chain. This activation facilitates the release of MVs. Importantly, this cascade does not substantially modify the release of smaller vesicles that are commonly linked to exosomes. Moreover, MVs and exosomes display unique protein compositions. MVs originating from melanoma cells exhibit a higher abundance of B1 integrin receptors and VAMP3, whereas exosomes are characterized by a notable presence of transferrin receptors, which are not found in MVs. The differentiation in content highlights the distinct roles and functionalities of these two categories of EVs.^46^

### 2.3 Formation of ABs

Apoptosis, a fundamental process of cell death in both healthy and cancerous cells, encompasses a sequence of stages that culminate in the creation of ABs, also referred to as apoptosomes. The process initiates with the condensation of nuclear chromatin, subsequent membrane blebbing, and ultimately results in the fragmentation of cellular content into discrete membrane-enclosed vesicles. ABs are specifically generated during programmed cell death, in contrast to exosomes, MVs, and retrovirus-like particles, which are released as part of normal cellular processes. ABs typically have a larger size, ranging from 500 to 4000 nm, and are distinguished by the presence of organelles inside the vesicles. Additionally, smaller vesicles with sizes ranging from 50 to 500 nm are released during the process of apoptosis. However, it remains uncertain whether these vesicles are a direct consequence of the membrane blebbing that is typically observed during apoptosis. Actin-myosin interactions partially contribute to the process of membrane blebbing.^47^

During typical development, macrophages predominantly phagocytose ABs and eliminate them in the immediate vicinity. The clearance process entails precise interactions between recognition receptors on phagocytes and alterations in the composition of the apoptotic cell’s membrane. Notable modifications involve moving phosphatidylserine to the outer layer of the lipid membrane, which is detected by Annexin V on phagocytes. Additionally, surface molecules undergo oxidation, resulting in the formation of attachment points for thrombospondin or the complement protein C3b. These attachment points are subsequently identified by phagocyte receptors. Annexin V, thrombospondin, and C3b are well-established indicators of ABs.^48^ The identification that exosomes and MVs have the ability to facilitate communication between cells by transferring genetic materials has generated enthusiasm in the scientific community regarding the potential of EVs as cancer biomarkers. The capacity to transfer genetic material is not limited to a single category of EVs. Research has demonstrated that ABs can be identified in the bloodstream of mice that have been implanted with tumor xenografts. Crucially, the absorption of ABs that contain genetic material from transformed cells can lead to substantial alterations in cells, such as the absence of contact inhibition *in vitro* and the development of a cancer-causing phenotype in living organisms. This suggests that ABs are capable of transferring genetic information as well.^49^

The composition of EVs

#### 2.3.1 Exosomes

Exosome formation and composition have been the subject of extensive research over the years. In 1952, Robert C. Hartmann and C. Lockard Conley conducted an experiment on human plasma in which they discovered that removing the pelleted plasma fraction after high-speed centrifugation inhibited plasma clotting.^50^ Peter Wolf discovered that these clotting inhibitors were platelet-derived vesicles ranging in size from 20 to 50 nm.^51^ In 1983, Bin-Tao Pan et al. and Priscilla S. Dannies et al. reported that transferrin receptors on reticulocytes interacted with approximately 50 nm active vesicles. These vesicles were discovered to be secreted into the extracellular environment by maturing sheep reticulocytes.^52,53^ EVs are categorized as MVs, exosomes, and ABs, among others. This classification is based on morphological and semantic characteristics.^54^ Particularly, exosomes are ILVs of MVBs with a diameter of 30–100 nm.^55^ Through high-resolution electron microscopy and advanced proteomic techniques,^56^ the composition of exosomes secreted by different cells has been elucidated. Not only the contents of exosomes reflect the composition of the donor cell, but they also indicate a mechanism for regulated sorting.^57^ Exosomes contain numerous proteins, including receptors, transcription factors, and enzymes, in addition to extracellular matrix proteins, lipids, and nucleic acids (DNA, mRNA, and miRNA). These components are found both inside and on the surface of exosomes.^58,59^

Extensive analysis of the protein composition of exosomes has revealed that some proteins are unique to the cell and tissue of origin, while others are common across by all exosomes.^56^ Typical examples of exosome-specific proteins include adhesion molecules such as Cell Adhesion Molecules (CAMs), integrins, tetraspanins, and Major Histocompatibility Complex (MHC) class I and II, which are present on B lymphocytes and dendritic cells, as well as transferrin receptors (TfR) on the surface of reticulocytes. In contrast, nonspecific exosome proteins include a variety of fusion and transferring proteins such as Rab2, Rab7, flotillin, and annexin, heat shock proteins such as Hsc70 and Hsc90, cytoskeleton proteins including actin, myosin, and tubulin, and proteins such as Alix that facilitate the formation of MVBs.^56,60^ In addition, the lipid composition of exosomes can be cell-specific or conserved. Lipids play a crucial role not only in preserving the shape of exosomes, but also in their biogenesis and in regulating the homeostasis of the recipient cells.^61−63^ The high density of lipids in the internal membrane of MVBs, such as LysoBisPhosphatidic Acid (LBPA), contributes to the formation of ILVs and, consequently, exosomes.^62,64^ The interaction between LBPA and Alix promotes the inward budding of the membrane of MVBs.^65^ Certain factors, including sphingomyelin, phosphatidylcholine, and Bone morphogenetic proteins (BMPs), assist in differentiating between distinct types of vesicles. While different types of MVs contain similar amounts of sphingomyelin and phosphatidylcholine, exosomes have a higher concentration of sphingomyelin. Endosomes, which are the source of exosomes, are the sole source of BMP.^66^ In addition, studies have demonstrated that exosomes transferred to target cells can alter the lipid composition of the recipient cells, specifically in terms of cholesterol and sphingomyelin, thereby influencing cell homeostasis^67−69^ Jalabert et al.^69^ determined that the lipid, protein, and miRNA composition of skeletal muscle (SkM) released extracellular vesicles (ELVs) from Ob/ob (OB) mice differs from that of wild-type (WT) mice. Atrophic insulinresistant OBSkM released fewer ELVs than WTSkM, as evidenced by a decrease in RAB35 and an increase in intramuscular cholesterol content. Liu et al.^70^ isolated exosomes from the perivascular adipose tissue (PVAT) and subcutaneous adipose tissue (SCAT) of C57BL/6J mice of wild type. The results revealed that PVAT-exosomes ameliorated macrophage foam cell formation and intracellular lipid accumulation and they significantly decreased the uptake of fluorescence-labeled oxidized low-density lipoprotein by macrophages. In addition, PVAT-exosomes promoted cholesterol efflux induced by high-density lipoprotein. Overall their findings suggest that PVAT-exosomes decrease macrophage foam cell PVAT protects the vasculature from atherosclerosis by regulating the expression of cholesterol transport proteins.

#### 2.3.2 MVs

MVs exhibit a proteomic profile that is impacted by the isolation technique employed, yet they consistently encompass a specific group of “marker proteins” irrespective of their cellular source. The presence of these proteins in MVs is essential because of their involvement in the biogenesis process. MVs are primarily composed of cytosolic and plasma membrane-associated proteins due to their formation through the outward budding of the cell’s plasma membrane. This encompasses proteins that aggregate at the surface of the plasma membrane, such as tetraspanins, which can be detected in concentrations up to 100 times greater in MVs than in the cell lysate.^71^

Additional frequently observed proteins in MVs include cytoskeletal proteins, heat shock proteins, integrins, and proteins that undergo post-translational modifications such as glycosylation and phosphorylation. The glycan-binding proteins present on the surface of MVs are of particular interest due to their potential significance in the targeting and interaction of MVs with other cells. Although this Review primarily examines the proteome of MVs, it is important to note that the glycome of MVs is also a noteworthy subject of investigation.^72^ Considering the process of MVs formation, it is anticipated that they would encompass proteins originating from the cytosol and the plasma membrane. In contrast, proteins that are linked to organelles such as mitochondria, Golgi apparatus, nucleus, and endoplasmic reticulum are generally reduced in MVs. This is because these organelles do not play a role in the formation of MVs. Nevertheless, it is crucial to acknowledge the absence of distinct indicators to differentiate between MVs and exosomes.^73^

#### 2.3.3 ABs

The composition of apoptotic bodies is noticeably distinct from that of exosomes and MVs. ABs differ from exosomes and MVs in that they contain intact organelles, chromatin, and a small amount of glycosylated proteins. As a result, it is common to observe increased concentrations of nuclear proteins (such as histones), mitochondrial proteins (like HSP60), Golgi apparatus proteins, and endoplasmic reticulum proteins (for instance, GRP78) in apoptotic bodies. Furthermore, the proteomic composition of apoptotic bodies closely resembles that of cell lysate, in stark contrast to the notable disparities observed between the proteomic compositions of exosomes and cell lysate.^74^

### 2.4 Methods for Characterization of EVs

Various techniques, including dynamic light scattering, nanoparticle tracking analysis (NTA), and resistive pulse sensing, are used to identify EVs. These methods cannot differentiate between EVs and other particles, such as lipoprotein particles, protein aggregates, and EVs aggregates. This can lead to the appearance that less pure isolates contain more EVs.^75^ Additional measurements, such as total protein and/or lipid content or quantification of EV marker proteins by ELISA or Western blot, can be used to improve accuracy.^76^ Size, a defining characteristic of exosomes, is an essential EVs separation parameter.^77^ The presence of certain proteins involved in MVB formation or exosome release, which can be used as positive protein markers, is another defining characteristic of MVB-derived exosomes. At least three of these indicators must be demonstrated.^78^

Transmission electron microscopy (TEM) is a powerful technique that enables the visualization of EVs at the level of a single particle, thereby revealing their size, shape, and potential contaminants. Uranyl acetate negative staining is the most commonly used technique for TEM imaging. Due to drying during the preparation process, however, this method may result in a ‘collapsed vesicle’ or ‘cup-shaped’ appearance.^79^ Cryo-TEM has become the gold standard for imaging biological entities in the modern era. This method involves a rapid freezing process that preserves the samples’ native hydrated structure. Cryo-TEM has a number of significant advantages, including the ability to distinguish genuine EVs from nonvesicular particles, precisely determine the size of EVs, and characterize heterogeneous EVs samples, including the detection of EV aggregates that may be present in the original sample or induced during isolation procedures. The combination of electron microscopy and immuno-gold labeling improves the phenotyping of EVs in complex media, such as pure plasma or heterogeneous mixtures.^80^ Other techniques, such as single EV-microarray and atomic force microscopy, can provide images of individual EVs as well as data regarding their biomechanical properties and size. Utilizing advanced imaging techniques such as TEM, particularly cryo-TEM, along with complementary methods such as immuno-gold labeling, single EVs-microarray, and atomic force microscopy has significantly advanced our understanding of EVs, their characteristics, and their biological functions.^81^

Flow cytometry is another effective method for analyzing EVs due to its capacity to perform high-throughput quantitative analysis of individual particles using light scattering and fluorescence. Nonetheless, because flow cytometers are primarily intended for cell analysis, certain requirements must be met to improve the precision and reproducibility of EVs analysis.^82^ Small EVs (less than 300 nm in size) are particularly difficult to analyze due to their weak fluorescence and scatter signals.^82^ Therefore, it is essential to calibrate flow cytometers, confirm the detection of individual EVs, and be aware of the platform’s sensitivity and the possibility of interference from unbound fluorescent probes.^83,84^ Despite these obstacles, single EV flow cytometric analysis has reached a point where nearly reproducible comparisons of EVs concentration measurements, such as circulating EVs in cardiovascular disease patients, can be made. CD61 and CD144 for platelets and endothelium, CD147 for cardiomyocytes, CD235a for erythroid-derived EVs, and CD45/CD3 and CD14 for leukocyte/lymphocyte- and monocyte-derived EVs are of interest for cardiovascular studies^85−87^

The optimal method for evaluating the functional activity of EVs would be a simple *in vitro* potency test that can serve as a surrogate for their *in vivo* activity. However, no universally applicable technique has yet been identified. In the field of cardiovascular medicine, the functionality of EVs is typically assessed using assays that measure *in vitro* angiogenesis, cell viability, contractility, or a combination of these. Common *in vitro* angiogenesis evaluation methods include the scratch assay,^88^ Boyden chamber migration assay,^89,90^ endothelial tube formation^91^ and vessel sprouting assays.^92−94^ When conducting dose–response experiments to determine the function of small electrically charged particles, it is essential to accurately measure their quantity and purity. Currently, there is no consensus on the best measure of quantity (number of particles, protein content, number of starting cells, etc.),^3^ but any normalization technique employed must be reported and justified. In addition, suitable controls should be implemented to confirm that the observed effects are mediated by EVs. For the therapeutic use of EVs, *in vitro* potency assays are required to predict the clinical efficacy of EVs preparations. However, this is contingent upon the ability to identify the mechanism of action and quantify the biological activity convincingly.^95^

#### 2.4.1 Exosome Profiling

Numerous studies have underscored the diagnostic and therapeutic potential of exosomes. While the majority of this research investigates their physical properties, chemical structures, and roles in different diseases, exosome profiling stands out as a specialized field of study. This subset of characterization focuses on the molecular composition of exosomes in minute detail. Through profiling, scientists can gain insight into the precise types of RNA, proteins, lipids, and other molecules found in exosomes. Such in-depth knowledge is essential for identifying specific markers, diagnosing cancer, formulating treatments, and comprehending the molecular mechanisms underlying cancer progression and drug resistance. Notably, even when exosomes are derived from the same parent cells, their molecular composition can vary. This difference can be attributed to variables such as the origin and activation status of the donor cell. In addition, exosomes can be classified into various types based on their size and structure. There are, for instance, large vesicles (90–120 nm), small vesicles (60–80 nm), and nonmembranous particles known as “exomeres” (approximately 35 nm). Each of these classes has a unique genesis, composition, and function.^96^

#### 2.4.2 Proteomic Profiling of Exosomes

Exosomes are complex vesicles that contain proteins from the transmembrane and endosomal protein families. There are notable transmembrane proteins, such as tetraspanins, in particular CD9, CD63, CD81, and CD82. In addition to MHC class I and II proteins, CD81 and galectin are immune regulatory molecules. In addition, adhesion molecules such as EpCAM and integrins are present. Endosomal proteins include cytoskeletal proteins such as actin and myosin, as well as cytosolic proteins. Notable members include heat shock proteins such as HSP60, HSP70, HSC70, and HSP90, and enzymes such as GAPDH, ATPase, Enolase, and Rab GTPases. These proteins are not merely passive passengers; they play important roles. Exosomes derived from metastatic cells are essential for processes such as migration and proliferation. In contrast, in nonmetastasizing cell-derived exosomes, they are essential for adhesion and polarity maintenance. Due to their significance, some of these proteins have emerged as exosome markers. The scientific community is conducting extensive research on these proteins to determine their relationship with cancer. This provides invaluable cancer diagnosis and treatment insights and tools. This investigation is at the forefront of innovative techniques such as multiplexed antibody arrays and protein aptamer arrays. These methods employ an array of detection techniques, ranging from fluorescent detection to label-free SERS.^97^

The observation that the protein composition of exosomes can vary depending on their size is intriguing. For example, TSG101 and Alix predominate in exosomes with a diameter of less than 80 nm. In contrast, CD63 and flotillin-1 are more prevalent in exosomes greater than 80 nm in size. Moreover, the protein profiles of exosomes mirror the environment of their originating cell. Exosomes typically contain proteins such as CD9, CD63, and CD81, which are involved in various cellular processes. However, their abundance can be affected by factors such as cell type and oxygen levels in the environment. This protein profiling serves as a guidepost for our understanding of diseases and the interactions between exosomes and the cells with which they communicate.^98^

#### 2.4.3 RNA Profiling

Exosomes are not only carriers of proteins and glycans, but also of nucleic acids, including mRNAs and various noncoding RNAs such as miRNAs, lncRNAs, and circRNA. Fascinatingly, these exosomal RNAs are protected from RNase degradation by a shield, ensuring their integrity. Their involvement in numerous cancer-related processes suggests that they have the potential to be game-changing noninvasive cancer biomarkers for diagnosis and ongoing monitoring. When comparing cancerous tissues to their healthy counterparts, there is a discernible difference in the expression patterns of exosomal RNAs. This difference is not just academic; it provides a window into the world of RNA regulation and can potentially help us better classify tumors.^96^ For context, the RNA profiles of exosomes from two distinct breast cancer cell lines, MDA-MB-231 and MCF-7, was examined. In the former, sno/scaRNAs predominate, while in the latter, tRNAs predominate. And this is not the end of the discoveries. Specific exosomal lncRNAs, such as HOTTIP, are found in higher concentrations in the serum of patients with gastric cancer, indicating their potential role as diagnostic markers. In addition, a multitude of circRNAs have been identified in diverse tissues. Some of these are tissue-specific and can aid in distinguishing cancerous from healthy samples. Exosomes derived from bone marrow mesenchymal stromal cells were the focus of a particularly intriguing study, which revealed differences in miRNA levels between healthy individuals and those diagnosed with acute myeloid leukemia.^99^

These insights from exosomal nucleic acids profiling are undeniably valuable, providing a new perspective on potential cancer markers and enhancing our understanding of diseases. However, there is a catch. Diverse techniques are currently employed to isolate and quantify these exosomes, resulting in inconsistencies. To truly harness the power of this information and make it clinically relevant, standard, reproducible procedures must be established.

#### 2.4.4 Lipidomic Profiling

Exosomes, which are distinguished by their lipid bilayer, provide insight into their formation, uptake processes, and roles in cellular communication when their lipid composition is analyzed. Although the lipid content of extracellular vesicles from various origins varies, they are primarily composed of plasma membrane lipids such as phospholipids, sphingomyelin, ganglioside GM3, and cholesterol. Intriguingly, when compared to their parent cells, vesicle membranes contain a higher concentration of specific lipids, endowing them with increased stability and a more robust structure.

In 2002, a groundbreaking discovery revealed the biological significance of vesicular lipid. In this study, it was demonstrated that sphingomyelin is primarily responsible for the angiogenic activity of tumor-derived vesicles. Furthermore, when bound to vesicles, specific prostaglandins initiate intracellular signaling pathways.^100^ Emerging evidence also implicates vesicle-bound lysophosphatidylcholine in processes such as dendritic cell maturation and lymphocyte movement regulation. Fascinatingly, membrane-bound molecules on vesicles, such as prostaglandin or lysophosphatidylcholine, exhibit greater biological activity than their free-floating counterparts, as observed with the vesicular ICAM-1 protein.^96^

Despite significant advances in lipid research, our current understanding covers only a small portion of the lipid universe. With the introduction of advanced techniques such as Liquid chromatography and mass spectrometry, we anticipate a more comprehensive profiling of lipid components across vesicles derived from various sources. These exhaustive studies are expected to shed light on the mysteries surrounding the roles of lipids in vesicle formation and their overarching biological functions.

#### 2.4.5 ML in Exosome Profiling

ML, often regarded as the foundation of artificial intelligence (AI), is a dynamic field based on the algorithmic principle. These algorithms empower it with the unique ability to learn from historical data, adapt, and subsequently refine its accuracy without human intervention. Its abilities have been widely recognized and utilized in the field of medical image analysis, where it plays a crucial role in clinical diagnoses. Complex medical images, for instance, are analyzed with greater precision, allowing clinicians to make more informed decisions.^101^ The application of ML to exosome research, however, is a relatively uncharted territory. Exosomes, which are minute vesicles with significant biological implications, frequently necessitate complex differentiation techniques. In many instances, a single molecular biomarker may lack the required specificity to distinguish between different exosomes. This presents a challenge, particularly when precision is essential. The solution may lie in the utilization of multibiomarkers, which, when coupled with the computational prowess of ML, can handle and predict outcomes from complex, multidimensional data.^102^

**Figure 2:**
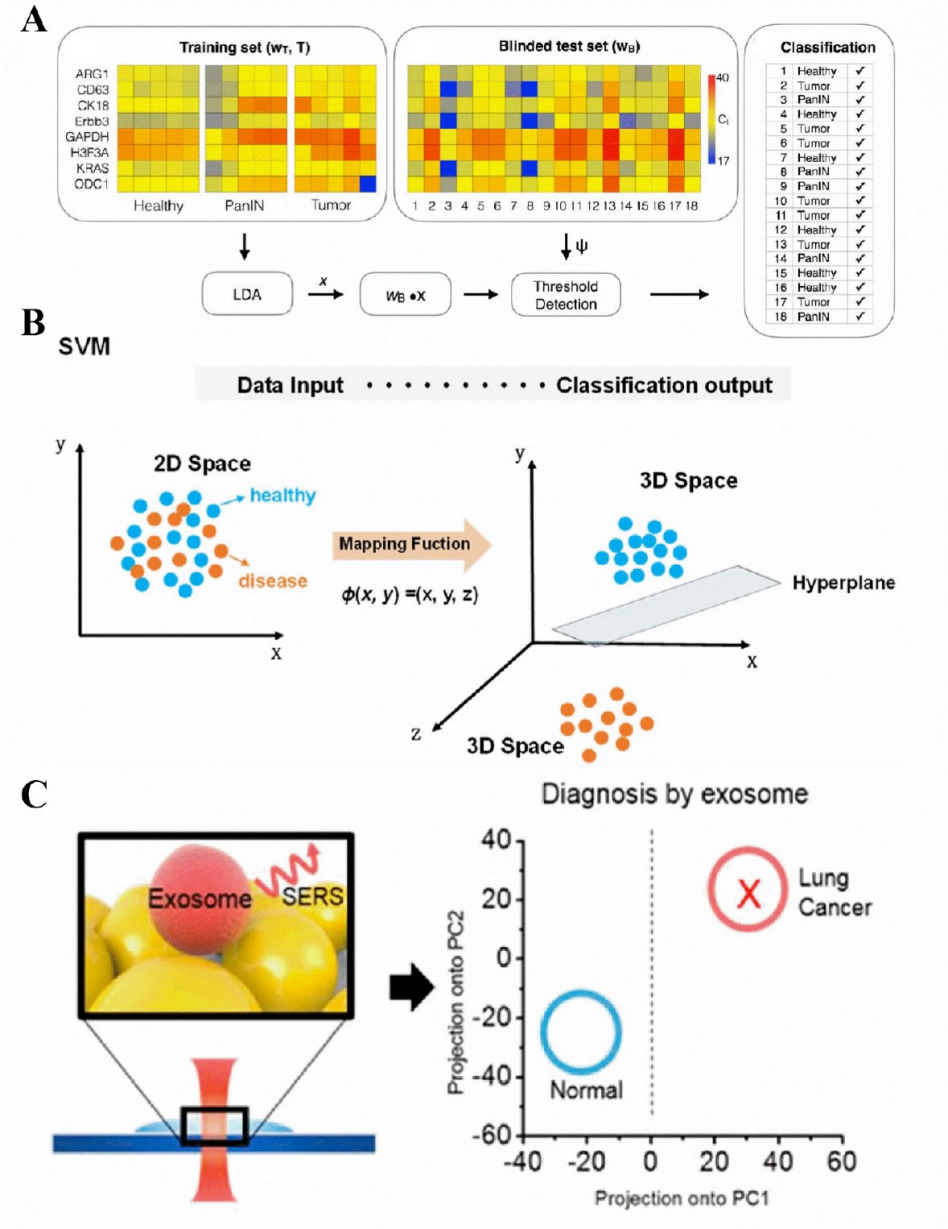
(A) An example of ML-based exosome classification. Reprinted with permission from ref (103). Copyright 2017 American Chemical Society. (B) A diagnostic model for performance of binary classification by SVM using different numbers of urinary extracellular vesicles (uEVs) surface biomarkers. In the SVM’s algorithmic structure, inputs are shifted to a high-dimensional realm through a nonlinear transformation, as determined by the inner product function. This permits the most efficient categorization within that space. In addition, the SVM method is accompanied by a confusion matrix that distinguishes between patients with urinary disease and healthy individuals using various biomarker combinations. Reprinted with permission from ref (105). Copyright 2021 American Chemical Society. (C) An example of a label-free and highly sensitive classification method of exosome by combining SERS and PCA. Reprinted with permission from ref (106). Copyright 2017 American Chemical Society.

Traditional methods of classifying exosomes frequently have their limitations. Typically, they rely on characteristics that must be identified beforehand. In contrast, ML offers a more adaptable and dynamic approach. It learns by observation. The model undergoes a learning phase by receiving a series of features and their corresponding labels and adjusting its internal parameters based on the input. Once trained, this model is capable of making rapid predictions on new, unexplored data sets (Figure 2A).^103^ However, this process is not devoid of obstacles. The model risks overfitting if the number of features introduced is disproportionately large compared to the examples. Dimensionality reduction techniques, such as Principal Component Analysis (PCA), come into play at this point. PCA can extract the essence of the data by reducing a large set of potentially correlated variables to a smaller set of uncorrelated variables known as principal components.^104^

In the world of exosome classification, researchers have access to a variety of models. These include models such as Linear Discriminant Analysis (LDA) and Support Vector Machine (SVM)^105^ (Figure 2B), as well as more complex models such as Convolutional Neural Networks (CNN). The selection of a model is frequently determined by the complexity of the prediction task at hand. For instance, traditional models such as LDA may produce satisfactory results for simpler tasks with fewer features. In contrast, advanced models, such as CNNs, may be more appropriate for more complex tasks with a rich feature set. Diverse characteristics serve as the basis for exosome classification. They consist primarily of proteins and nucleic acids, with data extracted using sophisticated techniques such as mass spectrometry, fluorescence, and quantitative polymerase chain reaction (qPCR). Additionally, methods like Fourier-transform Infrared Spectroscopy (FTIR) and Raman spectra are emerging as valuable tools, offering a plethora of features that can be harnessed for classification (Figure 2C).^106,107^

Nevertheless, it is essential to recognize that the incorporation of ML into exosome analysis is still in its infancy. The path ahead is replete with obstacles and possibilities. There is an urgent need for more extensive and diverse data sets, which can provide models with a more robust training ground. In addition, the development of more generalized methods for feature extraction can pave the way for ML techniques to find broader applications in this domain. To unlock the full potential of ML in exosome research, the focus should be on leveraging larger, higher-quality data sets, perfecting the art of feature engineering, and continuously expanding the scope of applications as research intensifies.

## 3 EVs-Loaded Hydrogels: A Promising Approach for Tissue Regeneration

Before exploring the details of hydrogel synthesis and applications, it is essential to establish the developing relationship between hydrogels and EVs in biomedical research. Hydrogels have distinctive properties that make them suited for a wide range of potential biomedical applications.^108^ For example, their hydrophilic porous structure allows them to absorb and retain water while maintaining their structural integrity, thereby facilitating particle-free diffusion.^109,110^ In addition, Young’s modulus and atomic force microscopy of hydrogels are essential for directing cell migration and proliferation within biomaterial scaffolds.^111^ The structural lattice of hydrogels, formed by a network of cross-linked monomer-polymer bonds, increases their adaptability to the microenvironment, simulating natural tissue.^112^ Moreover, hydrogels can degrade synchronized, promoting cellular growth within the microenvironment of a tissue. Boosting cellular migration, proliferation, and differentiation can facilitate using hydrogel scaffolds in fields such as tissue engineering, regenerative medicine, adhesive medicine, cell-encapsulating matrices, and drug-delivery systems.^113,114^

The rapid elimination of EVs by the immune system frequently impedes their clinical application, necessitating the development of delivery methods that ensure targeted delivery and maximize therapeutic efficacy.^115^ As biomaterials, hydrogels have demonstrated outstanding potential for enhancing tissue retention of EVs and providing a platform for their controlled release. When EVs are incorporated into these systems, hydrogels serve as delivery vehicles and interaction matrices.^14,116^ Hydrogels are frequently used for the prolonged, localized delivery of EVs that have been specially treated.^117^ For example, sEVs derived from human gingival MSCs and human placental-derived MSCs have been grown on a chitosan/silk hydrogel sponge.^118^ In fields such as angiogenesis, osteogenesis, oncology, immunology, and pain management, the hydrophilic and cross-linking properties of hydrogels allow for controlled drug release, making them highly influential.

The subsequent sections will examine the process of creating and categorizing hydrogels, EVs incorporation and controlled release in hydrogels, their primary uses in the field of drug delivery and tissue engineering, the characterization methods of EVs loaded hydrogels, and the contribution of machine learning in advancing EVs loaded hydrogel research. The discussion will consistently address the potential of hydrogels as carriers for EVs and the resulting implications for future biomedical applications.

### 3.1 Hydrogels: Synthesis, Classifications, Primary Biomedical Applications in Drug Delivery and Tissue Engineering

Due to their hydrophilic groups, such as −NH_2_, −COOH, −OH, −CONH_2_, and −SO_3_H, hydrogels are composed of a 3D network capable of absorbing and swelling in water.^108,119^ This network is typically composed of polymer chains that are cross-linked, but can also be created by cross-linked colloidal clusters.^109,120^ Their capacity to absorb water confers flexibility and softness.^121,122^ Hydrogels closely resemble living tissue because of their high-water content, soft structure, and porosity.^123,124^ Wichterle and Lim^110^ created a synthetic poly-2-hydroxyethyl methacrylate (PHEMA) hydrogel that was used as a filler for eye enucleation and contact lenses in 1960. Numerous studies from the 1970s to the 1990s demonstrate an increase in the use of hydrogels for drug delivery and bioactive compound release.^125−127^ Hydrogels found use in tissue engineering in the 1990s.^113,128,129^ From the 1970s to the 1990s, hydrogels were only used in surface environments, such as the eye and open wounds. The swelling–deswelling rate, stiffness, degradability, and size of hydrogels can be altered by modifying the hydrophilic and hydrophobic ratios, the initiator or polymer concentrations, and the reaction conditions (time, temperature, etc.).^130−132^ Due to *in situ* gelation after infection and the hydrogel’s responsiveness to stimuli, the biomedical applications of hydrogels are not restricted to the surface environment. This renders them a useful tool for a variety of biomedical applications.^133^

#### 3.1.1 Synthesis of Hydrogels

**Figure 3:**
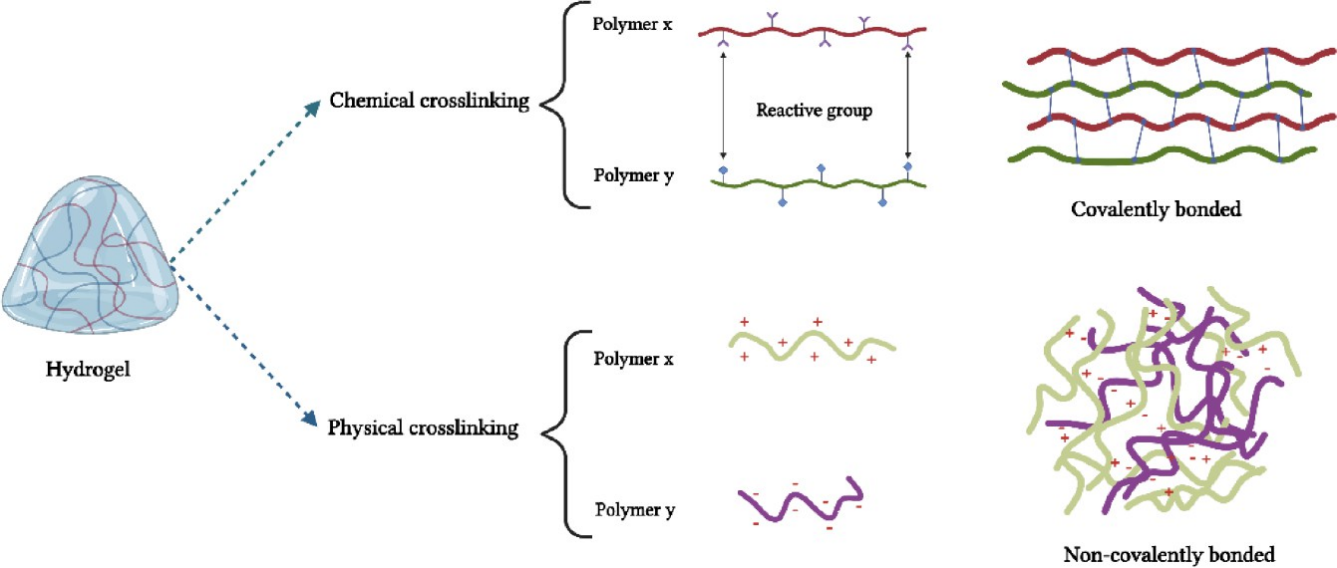
Graphical representation of the hydrogels physical and chemical cross-linking, highlighting the various bond types present.

Hydrogels consist of cross-linked polymer chains to form a 3D network structure. This cross-linking process can occur either after polymer chain synthesis or simultaneously with chain growth. Therefore, hydrogel synthesis can begin with monomers, prepolymers, or polymers. Regardless of the material type, hydrogels can be physically or chemically cross-linked, as depicted in Figure 3. Physical cross-linking in hydrogels can occur through diverse mechanisms, including hydrogen bonding, formation of amphiphilic grafts or block polymers, crystallization, ionic interactions, and protein interactions.^114,134,135^ Hydrogen bonds can form between molecules with N–H, O–H, or F–H functional groups, allowing polymers with these functional groups to form hydrogels via this mechanism. Amphiphilic graft and block polymer formation describe the self-assembly of polymers in hydrophobic or hydrophilic solvents due to their amphiphilic affinity.^136,137^ In the case of polymer chain crystallization, hydrogels can be synthesized by adjusting the crystallization temperature. The freeze–thaw and heating procedure is a common crystallization technique.^138,139^ Ionic interactions involve cross-linking due to ionic group attraction. Polymers added or modified with proteins using molecular medical technology, such as protein and genetic engineering, exhibit protein interactions. These polymers can synthesize hydrogels through antibody–antigen interactions or protein properties such as crystallization, functional groups, or hydrogen bonding.^140,141^

Methods for chemical cross-linking include chemical reactions, high-energy radiation, free-radical polymerization, and enzymatic processes. Complementary or pendant groups or polymers undergo chemical reactions with a cross-linking agent. Free-radical cross-linking is a characteristic of high-energy radiation and free-radical polymerization methods for hydrogel production. γ Rays or electron beam produces free radicals, whereas free-radical polymerization is facilitated by enzyme catalysts or ultraviolet excitation. Cross-linking is catalyzed by enzymes in polymers that have been altered or combined with enzyme-sensitive molecules.^142−144^

#### 3.1.2 Classifications of Hydrogels

**Figure 4:**
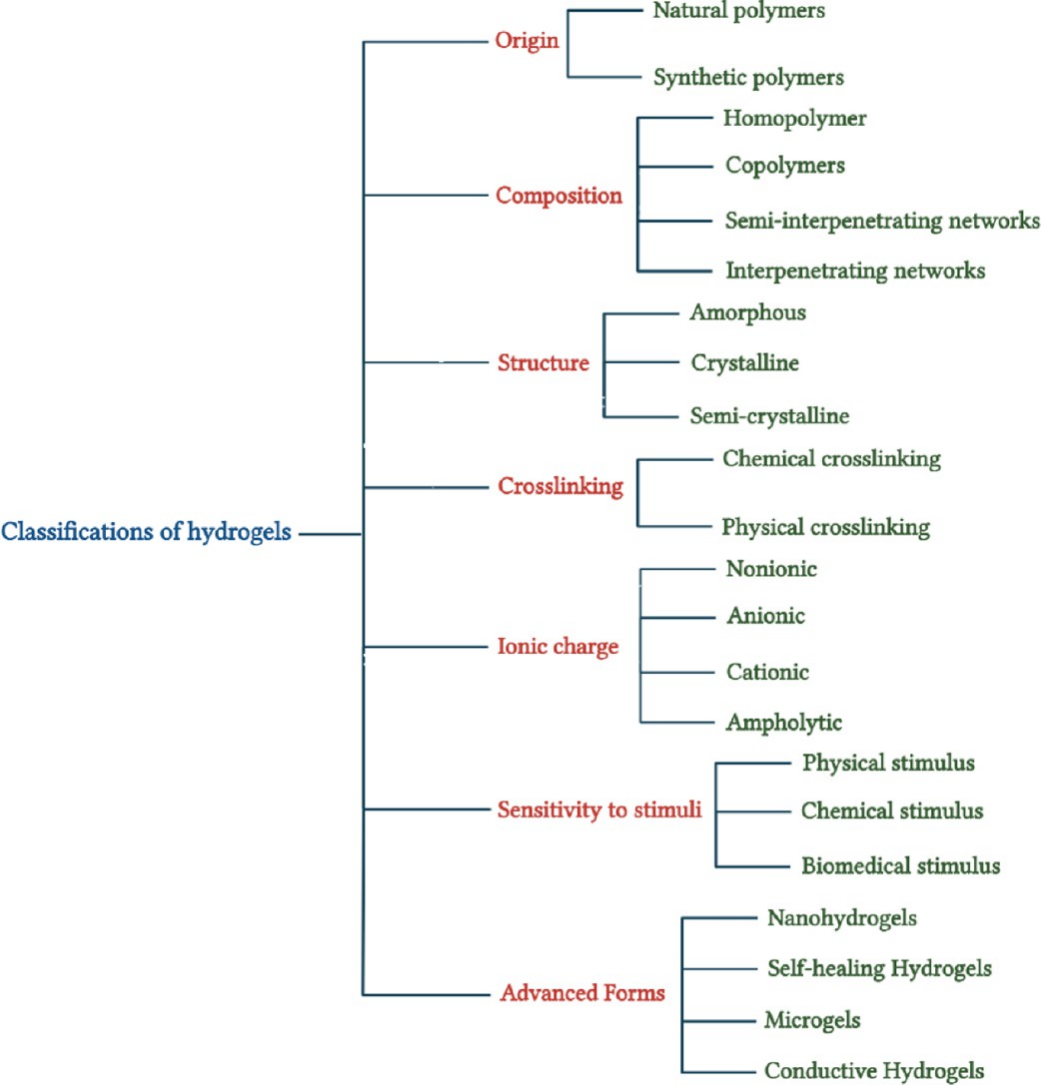
Classification of hydrogels.

Numerous factors, including their origin, composition, environmental responsiveness, cross-linking, properties, configuration, and ionic charge, can be used to classify hydrogels.^142,145,146^ (Figure 4) Comprehending their potential for tissue regeneration in conjunction with EVs requires an understanding of this classification. Hydrogels are produced by cross-linking polymer chains, which may be derived from natural, synthetic, or semisynthetic polymers. Hydrogels derived from natural materials, such as cellulose, chitosan, collagen, alginate, agarose, hyaluronic acid (HA), gelatin, and fibrin.^146,147^ Biocompatibility, bioactivity, and biodegradability are inherent properties, but stability and mechanical strength are typically absent. While natural hydrogels are generally safe, certain materials can occasionally cause allergic reactions, posing immunological risks for sensitive individuals.^148,149^ Synthetic hydrogels, on the other hand, are made from man-made polymers that are created by the polymerization of a monomer. Examples include poly(vinyl alcohol) (PVA), polyethylene glycol (PEG), poly(ethylene oxide) (PEO), PHEMA, poly-*N*-isopropylacrylamide (PNIPAM), poly(acrylic acid) (PAA), and polyacrylamide (PAAM).^145,146^ Despite the fact that only a few of these materials are biocompatible, they provide stability and mechanical strength. Natural polymers that have been chemically altered or a combination of natural and synthetic polymers are used to produce semisynthetic hydrogels. Examples include methacryloyl-modified gelatin (GelMA)^150^ and acrylate-modified hyaluronic acid (AcHyA),^151^ as well as combinations such as PEG-conjugated fibrinogen or gelatin and albumin.^152^ These hydrogels combine the bioactivity of natural hydrogels with the adjustability of their chemical parameters.^153^ This makes them well-suited for encapsulating and gradually releasing EVs in tissue regeneration applications.

Depending on their composition, hydrogel polymers can be classified as homopolymers, copolymers, semi-interpenetrating networks (semi-IPNs), or interpenetrating networks (IPNs).^154^ Homopolymer hydrogels contain polymer chains derived from a single type of monomer.^148,155^ Copolymer hydrogels, in contrast, are derived from two or more distinct types of monomers. Based on the arrangement of their monomer composition, copolymers can be further categorized as block, alternate, or random copolymers. It is possible to link the active side of copolymers to another monomer or copolymer, which increases their complexity. Both homopolymer and copolymer hydrogels are composed of only a single type of polymer chain. However, semi-IPNs and IPNs hydrogels are more complicated, containing multiple types of polymer chains. Semi-IPNs hydrogels are composed of a polymer network surrounded by linear polymer chains. Without the use of a cross-linking agent, these linear polymer chains are incorporated.^135^ IPNs hydrogels, on the other hand, are composed of two or more polymer networks that are cross-linked using a cross-linking agent. The mechanical strength and swelling properties of semi-IPNs and IPNs hydrogels are superior to those of homopolymer and copolymer hydrogels. This is because of their intricate structure, which permits greater flexibility and adaptability. These characteristics make them useful for a variety of applications requiring durability and flexibility.^156,157^

Hydrogels can be classified as amorphous, crystalline, or semicrystalline based on their structure. Hydrogels that are amorphous have a random molecular network structure.^158^ Crystalline hydrogels, on the other hand, consist of a polymer network structure that exhibits a certain degree of crystallization.^159^ In 1994, chemical cross-linking was used to create the first semicrystalline hydrogels, which contain both amorphous and crystalline regions.^160,161^ Physical cross-linking techniques, such as bulk and micellar polymerization, have been employed to produce semicrystalline hydrogels.^162^ The ability of semicrystalline physical hydrogels to rapidly transition from a solid-like state to a liquid-like state is one of their distinguishing characteristics. In semicrystalline chemical hydrogels, this characteristic is not observed. Due to their exceptional properties, semicrystalline physical hydrogels are ideal for the production of injectable hydrogels and shape-memory hydrogels.^163^

On the basis of their cross-linking method, hydrogels can be divided into two main categories: chemical hydrogels and physical hydrogels. Permanent junctions are formed through covalent cross-linking and the polymerization of end-functionalized macromeres in chemical hydrogels. Physical hydrogels, on the other hand, feature transient junctions composed of physical interactions such as ionic interactions, hydrogen bonding, and crystallization.^164^ Physical hydrogels typically have inferior mechanical properties compared to chemical hydrogels as a result of their weaker physical interactions. In contrast, physical hydrogels are typically softer and capable of reversibly transitioning between liquid and solid states.^165^

Based on their ionic charge, chemical hydrogels with their durable junctions composed of covalent cross-linking and polymerizing end-functionalized macromeres can be further divided into four groups: nonionic, anionic, cationic, and ampholytic. Other types of hydrogels contain electric charges within their polymer chains and are pH-sensitive due to the presence of ionic groups; nonionic hydrogels are the exception.^166^ Typically, anionic hydrogels have negative charges in their polymer chains, whereas cationic hydrogels have positive charges. By copolymerizing anionic and cationic monomers or by incorporating zwitterionic monomers into the polymer network, ampholytic hydrogels possess both negative and positive electric charges.^167^ These ionic hydrogels can form complexes with other charged molecules, making them suitable for drug delivery applications in the treatment of disease.^168^ This categorization is especially pertinent in the context of drug delivery applications, wherein the interplay between hydrogels and charged molecules, such as EVs, can be utilized for the purpose of targeted therapy.

Hydrogels react to a variety of physical, chemical, and biomedical stimuli. These stimuli can alter the physical properties of hydrogels, including their shape (transitioning from a swollen to a contracted state) and their ability to self-assemble.^169^ These stimuli can be found in solvents or environments external to the organism. Temperature, pressure, light, electric and magnetic fields are examples of physical stimuli.^170^ The pH level, ionic strength, solvent composition, and the presence of particular molecular species are examples of chemical stimuli.^171^ In contrast, biomedical stimuli involve reactions to antigens, ligands, and enzymes.^172^ On the basis of their responsiveness to these physical properties, conventional and intelligent hydrogels can be distinguished. Conventional hydrogels exhibit minimal alterations, primarily swelling in response to external environmental conditions, and have comparatively low mechanical strength. Smart hydrogels, on the other hand, are extremely sensitive to minor changes in external environmental conditions and can rapidly adjust their physical properties, such as mechanical strength, swellability, and stimulus sensitivity.^169^ These systems can be engineered to react to the specific conditions of the tissue, guaranteeing the most effective release and function of EVs for tissue regeneration.

Hydrogels can be further classified into advanced forms such as nanohydrogels, self-healing hydrogels, hydrogel microspheres, and 3D printed scaffolds, in addition to their basic classifications. These forms possess distinct benefits in the regeneration of tissue and organs, which are well-suited to the concept of EVs in tissue regeneration.^173^

Nanohydrogels, which have dimensions ranging from 1 to 100 nm, combine the properties of hydrogels and nanoparticles. These hydrogels possess a higher surface area-to-volume ratio, which amplifies their interaction with biological systems. Nanohydrogels primarily demonstrate intramolecular cross-linking, in contrast to conventional hydrogels like macro or microgels which usually involve intermolecular cross-linking. Nanohydrogels possess a structural distinction that enables them to more efficiently retain drugs that are incorporated within them, in comparison to larger hydrogels. Due to their diminutive size, they are able to efficiently penetrate and spread throughout tissues, making them ideal for the accurate administration of bioactive substances, such as EVs, in the field of regenerative medicine. Nevertheless, the utilization of nanogels, specifically in the realm of EVs loading and transportation, is still a relatively unexplored field, despite their comparable size to EVs. This provides an opportunity for additional investigation to examine the potential of nanogels as efficient carriers for EVs delivery in diverse biomedical applications.^173,175^

Microgels, also known as hydrogel microspheres, are hydrogels that exist in the micrometer size range. Microgels, unlike larger hydrogels, can be injected directly because they are smaller than the inner diameter of a syringe needle. They provide a greater proportionate surface area, which aids in the natural removal of substances and improves the ability to penetrate tissues, making them well-suited for repairing osteochondral joint injuries by delivering targeted and long-lasting treatment.^173^ Microgels have found extensive application in the biomedical domain for the encapsulation of cells, drugs, and EVs. For instance, MSCs-EVs enclosed in sodium alginate hydrogel microspheres have demonstrated encouraging therapeutic outcomes in acute colitis treatment.^177^ Additionally, they have been employed in the fields of cartilage regeneration^178^ and postinfarct heart tissue repair.^179^

Self-healing hydrogels are a notable breakthrough in biomaterials, distinguished by their capacity to autonomously repair and regenerate themselves following injury. This distinctive characteristic is attained by incorporating reversible covalent and noncovalent bonds, which can be easily broken and reformed without the need for external energy input.^180^ The hydrogels are typically designed with sacrificial bonds that are relatively weak, allowing them to dissipate energy during fracture and deformation. This enhances their durability and functionality. The self-repairing properties of these hydrogels can be triggered by external stimuli, such as light, heat, or changes in pH, or by the interaction of functional groups within the hydrogels.^181^ This inherent ability to undergo self-repair addresses a crucial constraint of conventional hydrogels, which lack the ability to spontaneously recover from damage. Self-healing hydrogels have expanded the range of hydrogel applications, especially in the field of biomedicine, resulting in the creation of multifunctional composite hydrogels. These hydrogels have demonstrated significant promise in 3D printing, drug delivery, and tissue regeneration.^181^ In 3D printing, they can be used to fabricate 3D scaffolds that can be engineered to replicate the inherent structure of bone, rendering them highly suitable for bone regeneration purposes.^182^ Integrating EVs into these scaffolds can enhance the regenerative capacity. Due to their capacity to adjust to damaged tissues and organs, as well as their injectable characteristics, they are highly suitable for wound healing applications.^183^ The versatility of these hydrogels is enhanced by their shear-thinning behavior, which is caused by physical cross-linking through hydrophobic interactions, electrostatic interactions, and hydrogen bonding. The design of self-healing hydrogels can incorporate various interactions, such as double-cross-linking or double-networking, to enhance their functionality for specific applications.^184^

Conductive hydrogels, a significant advancement in regenerative medicine, are commonly created by incorporating conductive polymers, metal or carbon-based substances, or ionic side groups or salts into flexible three-dimensional hydrogel networks. The objective of this integration is to attain electrochromic conductivity and augment the hydrogel’s interaction with biological systems. Nevertheless, a prevalent obstacle in their advancement is the occurrence of phase separation between the conductive additives and the polymer network, which has the potential to undermine both mechanical and conductive durability. In order to address this issue, it is essential to have a reliable and strong combination of the polymer network and conductive additive. These hydrogels need to have crucial characteristics like elasticity and suitability for use, especially for devices that convert human movement into electrical signals. They necessitate exceptional flexibility and the ability to stick firmly to human skin or biological tissues. Furthermore, in order to endure harsh environmental conditions and extend their lifespan, these hydrogels should possess properties such as resistance to drying and freezing, as well as the ability to self-repair. These hydrogels have a crucial role in the field of regenerative medicine by enhancing cellular behaviors, such as cytokine secretion, and thereby enhancing the microenvironment of injured tissues. Composite conductive hydrogels are frequently composed of conductive nanomaterials such as graphene and carbon nanotubes, or conductive polymers like polyaniline and polypyrrole (PPy). Their ability to align with the electrophysiological characteristics of nerve and heart tissues improves intercellular communication, which aids in the healing of nerve injuries and the restoration of heart muscle after a heart attack. For example, O. Gil-Castell et al. fabricated conductive nanofibers composed of polycaprolactone, gelatin, and polyaniline that exhibit optimal performance for cardiac tissue regeneration. These nanofibers were designed to serve as functional scaffolds with customized physicochemical properties.^185^ Hou Liu et al. created a neural stem cells loaded conductive hydrogel scaffolds (named ICH/NSC), consisting of amino-modified gelatin (NH2-Gelatin) and aniline tetramer grafted oxidized hyaluronic acid (AT–OHA). The ICH/NSC scaffold has effectively promoted the regeneration of spinal cord injuries (SCI) and shows potential as a promising therapeutic approach for repairing SCI.^186^ Conductive hydrogels, when used in conjunction with EVs, can create a synergistic impact by improving tissue repair and regeneration. An electroconductive hydrogel nerve dressing composed of tannic acid (TA) and PPy loaded with bone marrow-derived mesenchymal stem cells (BMSCs)-sEVs was developed. This dressing is specifically designed to treat diabetic peripheral nerve injury (PNI) and aims to restore functionality and alleviate pain. sEVs within this system have the ability to regulate the polarization of M2 macrophages through the NF-κB pathway, thus reducing inflammatory pain in diabetic peripheral neuropathy. In addition, the system improved the regeneration of myelinated axons through the MEK/ERK pathway both *in vitro* and *in vivo*. Overall the results indicated that the dressing system holds significant potential for promoting nerve regrowth, restoring function, and alleviating pain in individuals with diabetic PNI.^187^

#### 3.1.3 Hydrogels Applications in Drug Delivery and Tissue Regeneration

Hydrogels have a wide range of applications due to their unique compositions and adaptability to various conditions. Their exceptional water concentration enables them to be adaptable, making them suitable for a wide range of industrial and biological applications.^154^

Controlled drug delivery systems were created to circumvent the limitations of conventional drug formulations by releasing medications at predetermined rates over predetermined time periods. In these systems, hydrogels, which have a strong affinity for water and can expand due to their cross-linked matrix, are frequently employed. Their porous structure makes them highly permeable to various medications, allowing for the targeted and controlled delivery of drugs, including the incorporation of EVs.^188,189^ Hydrogels are valued in drug delivery research due to their ability to sustain the release of medicinal substances over extended time periods, thereby preserving high concentrations of active medicinal material at designated sites.^190^ Physical (electrostatic interaction) and chemical (covalent) methods can be utilized to strengthen the bond between the drug or EVs and the hydrogel matrix and prolong drug release.^188^ Additionally, hydrogels can protect medications and EVs from harsh conditions while ensuring their proper release. Local changes in pH, temperature, the presence of certain enzymes, or external physical stimuli can trigger drug release from hydrogels.^191^ Hydrogels containing water-filled pores that have been covalently cross-linked are used for drug loading and unloading, with the release controlled by a stimulus-induced response. Changes in pH are utilized by ionically cross-linked hydrogels to effectively administer drugs.^112^ The process of drug loading and unloading causes the expansion and contraction of hydrogels. The swelling of hydrogels decreases as the cross-linking density of the polymer network increases, resulting in a more stable polymer network.^192^ This swelling relaxes and expands the polymer chain, allowing the medication to diffuse. This method, known as Case II transport, ensures time-independent, continuous drug release kinetics. Unusual transport, which combines diffusion and swelling processes, facilitates drug release by permitting active pharmaceutical ingredients to diffuse from regions of higher concentration to regions of lower concentration within the hydrogel.^193^ Hydrogels rely on reservoir or matrix technology for diffusion-controlled drug delivery, where drugs are released through diffusion via water-filled channels or a mesh structure within the hydrogel.^194^ A hydrogel membrane coats a drug-filled core in the reservoir delivery system, resulting in capsules, spheres, or slabs with a concentrated drug core that promotes consistent and sustained drug release. The matrix technique employs a macromolecular network or mesh within the hydrogel, enabling diffusion-based drug release.^188,190,195^ Bioinspired hydrogels represent a newer development in hydrogel-based drug delivery systems. These 3D materials mimic the biological microenvironment pertinent to specific medical conditions, thereby facilitating the study of targeted drug delivery mechanisms, *in vivo* therapy performance, disease progression, and other phenomena. They are particularly effective in the field of complex diseases, such as cancer, whose intricate molecular and physiological changes require continuous monitoring.^196,197^

In the medical sciences, tissue regeneration *in vivo* is of paramount importance. A mixture of patient cells and a polymer is prepared in a laboratory prior to implantation. Hydrogels distinguish themselves in this field by substituting for the natural extracellular matrix, thereby promoting cell growth and tissue development. This synthetic matrix, which is enriched with growth factors and metabolites, is intended to connect cells and modulate tissue structure, ultimately replacing damaged or lost tissues.^196,198^ Hydrogels have carved out a niche in tissue engineering due to their mechanical strength, biocompatibility, biodegradability, and close resemblance to the extracellular matrix of the body. These hydrogel frameworks are multifaceted and show promise for the regeneration of diverse tissues, including nerves, cardiac tissue, cartilage, and bones.^199^ In animal models, 3D-printed collagen-chitosan hydrogels have been highlighted for their potential to reduce scarring, promote nerve fiber regeneration, and facilitate functional recovery.^200^ The use of a mixture of HA, alginate, and fibrin as 3D printing ink is another innovative technique that paves the way for nerve tissue regeneration.^201^ Additionally, HA and cellulose-based hydrogels have shown promise in repairing central nerves.^202^

Li J et al. have developed a novel hydrogel containing horseradish peroxidase and choline oxidase. When this hydrogel is infused with BMSC, it demonstrates remarkable abilities for promoting cell health and neural differentiation, providing hope for the recovery of mice with traumatic brain injuries.^203^

Hydrogels may have the potential to direct the differentiation of human induced pluripotent stem cells (hiPSCs) into nucleus pulposus cells, as indicated by prior research. Encapsulated hiPSCs within a hydrogel, which has demonstrated promise in cardiac differentiation, indicated a promising future for regenerative therapies.^204^ Another work utilized a novel hydrogel system to encapsulate adipose-derived stem cells, resulting in positive outcomes for the treatment of cardiac damage following heart attacks.^205^ Simultaneously, in a recent research, a 3D cardiac mesh tissue that has demonstrated efficacy in preserving cell health and enhancing cardiac function in rats was developed.^206^ Additionally, silk fibroin has been lauded for its ability to induce pacemaker cells to resemble authentic sinoatrial-node cardiomyocytes.^207^

In the field of cartilage tissue engineering, Ravi et al.^208^ has unveiled a hydrogel that enhances cell growth and reshapes cell structure, suggesting its potential for cartilage restoration. Another intriguing hydrogel blend has been used to fabricate a 3D-printed knee scaffold that has demonstrated efficacy in boosting collagen production and cell proliferation, indicating its suitability for cartilage repair.^209^

In the field of bone regeneration, recently a gelatin hydrogel variant that can stimulate bone growth even in the presence of inflammation has been identified.^210^ In addition, a 3D-printed hydrogel scaffold has been shown to promote bone regeneration in rabbit tibia defects.^211^ In animal studies, a multitude of natural, synthetic, and semisynthetic hydrogel scaffolds have demonstrated their potential for use in tissue repair.^212,213^ It is essential to note, however, that these discoveries are still in their infancy, confined to the preclinical stages, and require additional research to determine their clinical applicability.

### 3.2 EVs Incorporation in Hydrogels

There are typically three ways to incorporate EVs into hydrogels.^214^ The first step entails combining EVs with a hydrogel precursor solution, and subsequently initiating cross-linking through the use of cross-linking agents or physical methods. This technique utilizes an active precursor to form covalent cross-links, resulting in hydrogels that possess adjustable characteristics such as customizable mechanical strength and degradation rates. These characteristics render them well-suited for EVs encapsulation. Moreover, the utilization of macromonomers, which are commonly derived from biocompatible polymers, augments the safety of EVs delivery. Nevertheless, the incorporation of additional components such as cross-linking agents may potentially undermine the structural integrity of the EVs.^215^

The second method involves the simultaneous blending of EVs with polymers and cross-linking agents. This method enables the containment of EVs during the process of network formation and is especially efficient for in situ gelation. This technique enables the formulation to be injected directly into the desired location, where it subsequently solidifies into a gel. This feature makes it beneficial for delivering EVs to specific areas within intricate anatomical structures. This process often utilizes a dual-cavity syringe.^216^

The third technique, referred to as the “breathing” or swelling method, entails submerging a preformed and freeze-dried hydrogel in an EVs suspension. This enables the hydrogel to expand and assimilate the EVs. This approach safeguards the essential variables from any detrimental consequences of polymerization conditions and facilitates uncomplicated lyophilization for convenient transportation. The breathing technique additionally facilitates a homogeneous dispersion of EVs throughout the hydrogel. Nevertheless, the hydrogel’s porosity must be adequately spacious to accommodate the EVs. Incorporating EVs into hydrogel pores becomes challenging when the size of the EVs exceeds that of the pores. Furthermore, EVs that are not securely bound to the matrix have the potential to pass through larger pores.^217^

In addition to direct encapsulation, augmenting the interaction between EVs and hydrogels via physical or chemical methods can enhance their affinity and retention. The phospholipid bilayer membranes of EVs, which are decorated with a variety of proteins, provide locations for interaction. Phosphate groups present on phospholipids and functional groups such as amide, amine, hydroxyl, and carboxyl found on proteins and peptidoglycans have the ability to create bonds with polymer polar groups through hydrogen bonding or van der Waals forces. For instance, in a research investigation, Tartaric asid was employed to chemically bond photo-cross-linkable gelatin methacrylate (GM) and PPy, resulting in the formation of GMP hydrogels. Tartaric acid interacts with the amide bonds in glycolic monomers and the nitrogen groups in PPy. The polyphenol groups in tartaric acid also establish reversible hydrogen bonds with the phosphate groups on EVs surfaces, effectively immobilizing EVs in the hydrogel and facilitating prolonged release over an extended duration.^218^

The electrostatic interactions between EVs and biomaterials exhibit a significantly higher strength and efficacy in retaining EVs, as compared to the weaker hydrogen bonds and van der Waals forces. The presence of anionic phosphatidylserine and charged residues on the glycocalyx of EVs has led to investigations on the use of positively charged polymers for integrating EVs.^218,219^ Chitosan-based hydrogels, due to their mild cationic charge, have shown a prolonged release of EVs *in vitro* for a duration of 6 days. In an experimental model using rats with diabetes, wounds that were treated with chitosan-EVs composites exhibited a faster healing process compared to wounds treated with chitosan alone or left untreated.^220^

The covalent bonding between EVs and polymers allows for the immobilization of EVs within hydrogels. The EVs are only released when the hydrogel network degrades or when the bonds between the EVs and polymers are broken. These bonds can be intentionally designed to degrade over a period of time or in reaction to external triggers. A study was conducted to create a hydrogel using photocleavable linkers that were connected to EVs. Hemagglutinin, which was originally combined with cysteine to bind to thiol groups, was chemically cross-linked with EVs that were modified with a photocleavable linker. When subjected to UV light, this hydrogel system has the ability to release EVs, which remain stable and intact within a living organism for multiple days, thereby facilitating the regeneration of tissues.^221^

Moreover, the presence of adhesion molecules on the surfaces of EVs can augment their attraction to biomaterials. EVs have the ability to attach to fibronectin and collagen by means of integrins, as well as to Hemagglutinin by means of CD44.^222^ Researchers have created EVs that bind to collagen in order to improve their effectiveness in treating diseases. These EVs, which have been altered with a collagen-binding peptide known as SILY, are specifically engineered to bind to collagen in the extracellular matrix. This binding enhances their ability to remain in place and enhances their effectiveness after transplantation. They exhibited enhanced adherence to collagen and preserved their biological functionalities *in vitro*. In a mouse model of limb ischemia, the modified EVs exhibited extended retention, diminished inflammation, and markedly enhanced muscle and vascular regeneration in comparison to unmodified EVs. Due to collagen’s widespread occurrence in various tissues, SILY-EVs possess the capacity to enhance EVs-based treatments for a wide range of diseases and could be utilized to augment collagen-based biomaterials in the field of regenerative medicine.^223^

### 3.3 Analysis of EV Release Dynamics and Characterization of Released EVs from Hydrogels

The release of EVs from hydrogels is regulated by various physicochemical mechanisms, such as diffusion, degradation, and mechanical deformation.^223,224^ Diffusion is the main mechanism for controlled delivery systems, where particles in a fluid are driven by thermal motion. The rate at which EVs are released through diffusion is influenced by factors such as the gradient of chemical potential, the distance they need to travel to exit the gel, and their mobility within the hydrogel.^225^ The release process is significantly influenced by the mesh size of the hydrogel, the characteristics of the polymer, and the interactions between EVs and the polymer. EVs with a size smaller than the hydrogel’s mesh size are released via diffusion. At first, there is a swift and intense release of EVs caused by the elevated concentration of EVs in the gel. As the level of concentration decreases, the rate of release decelerates, resulting in a more prolonged release that adheres to a first-order model. The length of time it takes for EVs release can vary from hours to days, depending on the characteristics of the hydrogel and its rate of degradation. Equilibrium is achieved in the release process when the concentrations of EVs inside and outside the system become equal. Any remaining EVs are released only when the hydrogel completely degrades.^226^ When the hydrogel’s mesh size closely matches the size of the EVs, it creates frictional resistance that hinders their movement, causing a slowdown. If the size of the EVs exceeds the mesh size, their only means of exiting is through the degradation or swelling of the hydrogel. Higher cross-link density or polymer concentration results in hydrogels with reduced pore size, leading to increased retention of EVs. Hydrogels that undergo expansion in reaction to changes in the surrounding conditions, such as alterations in pH levels, show great potential for the delivery of EVs.^227^ Alginate hydrogels containing nanoparticles of comparable size to EVs can be triggered to release the EVs by changes in pH. This functionality is especially advantageous for the treatment of solid tumors or wound healing. pH-responsive hydrogels can facilitate the controlled release of EVs at the specific locations of injury or tumor sites, which exhibit distinct pH levels in comparison to healthy tissues.^228^

Manipulating the degradation of the hydrogel mesh allows for precise control over the release of EVs from hydrogels. As the hydrogel network undergoes degradation, the size of the mesh expands, thereby promoting the mobility of EVs.^229^ Polymer degradation can happen at the cross-links or backbone of the polymer as a result of hydrolysis, enzymatic activity, or other factors.^230^ Hydrolyzable cross-linking molecules such as anhydrides, esters, and amides are frequently employed. Notably, ester linkages in PEG hydrogels are a prominent example.^231^ By manipulating the cross-linking density and composition of these hydrogels, it is possible to regulate the rate at which EVs are released in a controlled environment for durations spanning from 6 to 26 days, while simultaneously preserving their structural and functional integrity.^232^ Hydrogel-based EVs depots have demonstrated superiority over unbound EVs solutions in skin wound healing models.^233^ As an illustration, EVs in hydrogel groups gather at the site of injection, while free EVs have a tendency to spread out and accumulate in organs such as the liver and kidneys. Matrix metalloproteinases (MMPs) are utilized to produce hydrogels that are responsive to enzymes and capable of degrading gelatin. These hydrogels are then employed for loading EVs.^234^ GelMA hydrogels degrade in the presence of MMPs, which enables controlled release of EVs and enhances their retention in heart tissue compared to EVs that are not encapsulated in the hydrogels.^235^ Moreover, hydrogels can be engineered to undergo degradation in reaction to external environmental factors, such as alterations in pH or exposure to UV light.^236^ Hydrogels that respond to changes in pH, such as those composed of HA, Pluronic F127, and poly-ε-l-lysine, exhibit accelerated degradation in acidic environments, resulting in enhanced release of EVs. Hydrogels containing ortho-nitrobenzyl ester groups have the ability to degrade when exposed to UV light.^237^ Through the process of fine-tuning properties such as degradation rate and mechanical characteristics, hydrogels can be optimized to release EVs in coordination with the material’s degradation. This has been demonstrated in studies that utilized HA hydrogel scaffolds.^238^

Hydrogels that undergo expansion in reaction to changes in the surrounding conditions, such as alterations in pH levels, show great potential for the delivery of EVs. For example, alginate hydrogels containing nanoparticles of comparable size to EVs can release the EVs in response to changes in pH. This feature is especially beneficial for the treatment of solid tumors or wound healing. pH-responsive hydrogels can facilitate the controlled release of EVs at the specific sites of injury or tumors, which exhibit distinct pH levels in comparison to healthy tissues.^239^ Mechanical deformation of the hydrogel network can also affect the release of EVs. External forces, such as mechanical stress, magnetic fields, and ultrasound, can induce this deformation, leading to a change in shape of the hydrogels and subsequent release of the EVs. The application of mechanical force can induce convection within the hydrogel, resulting in the intermittent and temporary release of EVs. Hydrogels containing magnetic nanoparticles (NPs) exhibit high sensitivity to magnetic fields.^240^ By subjecting these hydrogels to a magnetic field, it is possible to alter their shape, resulting in the formation of large pores and enabling rapid deformation without causing any physical harm. This process greatly aids in the quick release of enclosed particles.^241^ Ultrasound, a different external force, has the ability to temporarily disturb the structure of the hydrogel. This disturbance not only initiates the liberation of hydrogel components such as nanobubbles, liposomes, and NPs but also amplifies the infiltration and absorption of medications within tissues.^242^ Nevertheless, it is crucial to acknowledge that external factors have the potential to impair or disrupt the operation of EVs. In order to reduce this risk and prevent permanent mechanical harm to hydrogels, self-healing hydrogels can be utilized. These hydrogels possess reversible physical cross-links, allowing them to restore their networks following mechanical damage, thereby preserving their structural integrity and functionality.^243^

The quantification of EVs released from hydrogels is accomplished using techniques such as micro bicinchoninic acid (BCA) assays, ELISA, or fluorescent labeling. These techniques quantify the concentration of EVs that are released into the surrounding medium, yielding information about the rate and effectiveness of their release.^244^ An additional crucial aspect is the tracking and monitoring of the behavior of electric vehicles (EVs) within hydrogels. Fluorescent markers such as PKH26 and other tracers like amphiphilic NIR-fluorescent probes and nanogold have been used.^245^ These markers play a crucial role in examining the kinetics of EVs, encompassing their secretion and engagement with specific cells. Ultimately, it is crucial to guarantee that the EVs preserve their structural integrity and biological functionality after being released. Electron microscopy and fluorescence microscopy are employed to evaluate the structural soundness of released EVs.^245,246^ Furthermore, the biological efficacy of these EVs is assessed using indirect techniques, including cell proliferation, migration assays, and tube formation assays involving endothelial cells. Conducting these qualitative analyses is essential to verify that the released EVs maintain their therapeutic efficacy.^247^

### 3.4 Utilizing ML in the Development of EVs-Loaded Hydrogels for Tissue Regeneration

The unique chemical and structural properties of hydrogels, especially those loaded with EVssvm, are closely connected to their potential in applications such as tissue engineering scaffolds and drug and gene delivery systems. These properties play a crucial role in determining their physical, chemical, biological, and biomechanical characteristics, which are necessary for successful tissue regeneration.^248^ Although there has been significant research conducted on polymer design and evaluation for these applications, it is still difficult to establish a direct relationship between a polymer’s inherent chemistry, structure, and its performance in hydrogel systems. ML is a vital tool in various areas, including the development of peptide hydrogels, improving cell adhesion on polymer surfaces, advancing gene delivery mechanisms, studying polymer-immune system interactions, and optimizing bioinks for 3D bioprinting.^249^ The incorporation of machine learning not only assists in improving the characteristics of hydrogels, but also plays a crucial role in the development of hydrogels loaded with exosomes, thereby increasing their usefulness in tissue regeneration and other areas of biomedicine.

#### 3.4.1 Self-Assembling Peptide Hydrogels

The advancement of self-assembling peptide hydrogels holds great promise in the realm of tissue regeneration, especially in their capacity as carriers for EVs specially sEVs. Utilizing ML to comprehend and forecast the characteristics of these peptide hydrogels can greatly augment their utilization in regenerative medicine. Fei et al.^250^ have recently shown how ML can be used to analyze the connection between the chemical structures of peptides and their capacity to create hydrogels. The physical and chemical properties of the hydrogel matrix play a vital role in effectively encapsulating and releasing sEVs, making this particularly important for sEVs-loaded hydrogels. They conducted a study where they synthesized a substantial quantity of peptides and examined their properties related to gel formation. Through the utilization of ML techniques, they successfully forecasted the peptides that were most prone to forming hydrogels. This type of predictive modeling is extremely valuable in the process of designing peptide hydrogels for sEVs delivery. It has the ability to identify candidates that possess optimal properties for encapsulating and preserving the bioactivity of sEVs. Furthermore, the utilization of ML in this particular context effectively tackles a significant obstacle in the field, which is the requirement for hydrogels possessing accurate and adjustable characteristics. ML can facilitate the synthesis of peptide hydrogels that are precisely customized for sEVs delivery in tissue regeneration applications by identifying the key structural parameters for gel formation. The incorporation of ML into the fabrication process of self-assembling peptide hydrogels signifies a notable advancement in the production of sEVs delivery systems that are both more efficient and effective. This method not only improves our comprehension of hydrogel formation but also creates new opportunities for tailoring hydrogel structures to meet specific regenerative requirements.^250^

#### 3.4.2 Cell Adhesion and Polymer Properties

**Figure 5:**
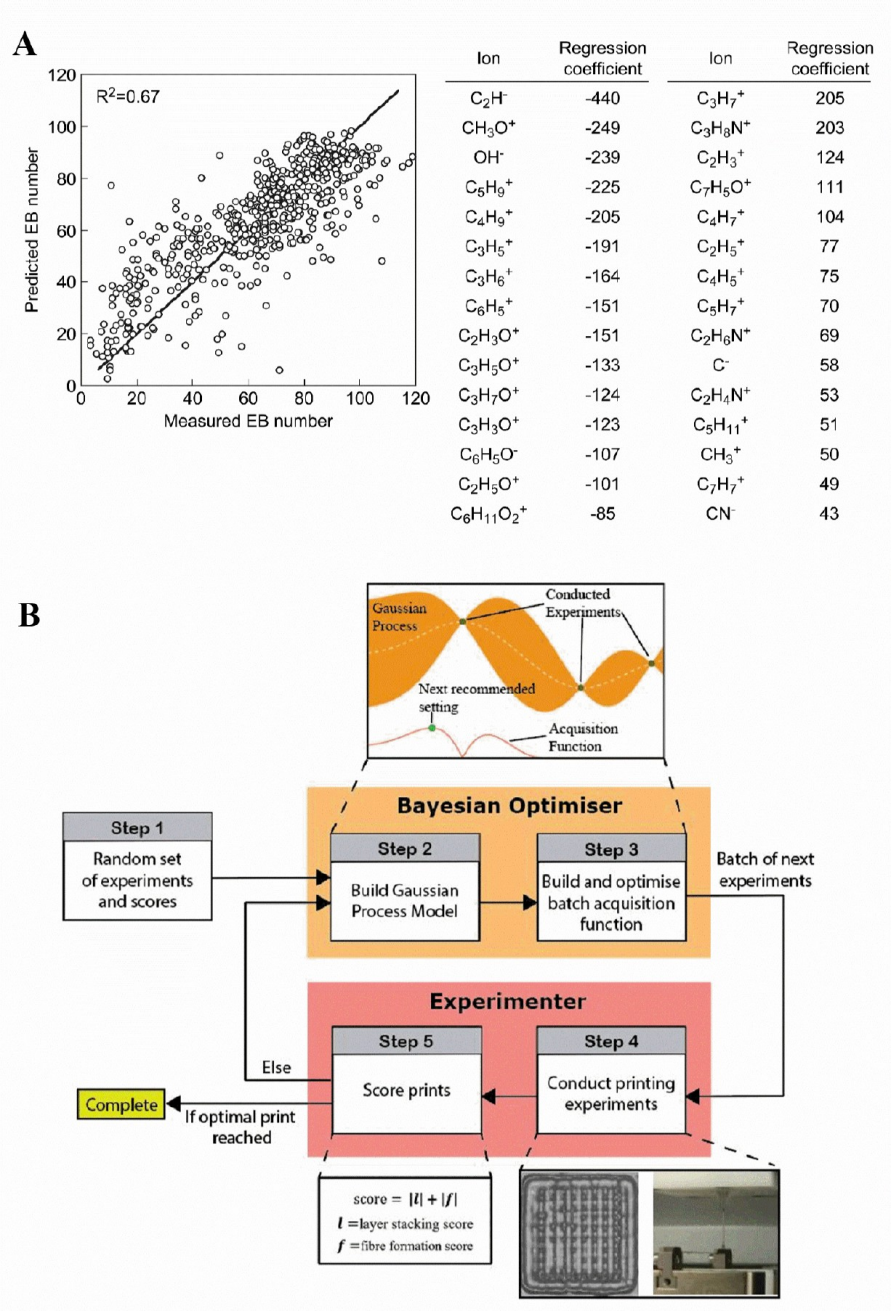
(A) The 576-sample PLS model demonstrates agreement between measured and predicted cell adhesion. List of the 30 most influential ToF SIMS ions on cell adhesion. Negative and positive ToF SIMS ion regression coefficients inhibit and promote cell adhesion, respectively. Reprinted with permission from ref (252). Copyright 2010 Elsevier. (B) The diagram depicts an example of the optimization framework utilized for bioprinting using Bayesian optimization algorithm. The framework commenced with a series of randomized, scored experiments. The Bayesian optimizer was initialized with these experimental outcomes, consisting of pairs of printer settings and their associated printing score. Within the optimizer, a probabilistic model of the system is constructed and used to suggest the next set of experiments (printer settings) to be conducted. Reprinted with permission from ref (257). Copyright 2010 Elsevier.

The interaction between cells and the hydrogel matrix is a crucial aspect in the field of tissue regeneration involving exosome-loaded hydrogels. Utilizing ML to examine the bonding of cells on polymer surfaces yields crucial knowledge for enhancing the development of these hydrogels. The study of the attachment of human embryonic stem cells (hES) to polyacrylate surfaces highlights the importance of surface chemistry and topography in influencing cellular reactions.^251^ Researchers fabricated polyacrylate microarrays from various acrylate monomers. These were then exposed to hES, and the cellular responses were measured. Using time-of-flight secondary-ion mass spectrometry (ToF-SIMS), significant correlations were observed between the frequency of hES colony formation and certain polyacrylate properties, despite the absence of a direct correlation. Additional analysis revealed that particular ions influenced colony formation. Certain ions, for instance, had a positive influence on colony formation, while others had a negative influence. This analytical method was also used to investigate the adhesion behaviors of human embryoid body cells (hEB) to polyacrylate. By examining the adhesion behaviors of hEB, Yang et al.^252^ demonstrated further the efficacy of this analysis. Certain molecular fragments were found to play an important role in promoting or inhibiting cell adhesion. In addition, purely computational models were developed to predict hEB adhesion, and they demonstrated a higher predictive accuracy than models that utilized experimental data (Figure 5A). Lastly, using an Artificial Neural Network (ANN), the correlation between experimental results and predicted fibrinogen adsorption amounts was analyzed. Extremely accurate predictions were made. This ML method also elucidated the factors influencing protein adsorption on SAMs, highlighting the importance of terminal groups over the length of alkyl chains. Similarly, the water contact angle of SAMs was predicted using an ML technique.^253^ These models may allow for the anticipation of the interaction between different polymer compositions and surface treatments with cells, thereby aiding in the development of hydrogels that are more effective in delivering exosomes to specific tissues.

#### 3.4.3 Gene Therapy and Polymer Vectors

Gene therapy has the potential to treat a variety of diseases, but its success is frequently contingent on the efficient and secure delivery of genes into host cells. Polymers, lauded for their biocompatibility and low toxicity, are viewed as the next frontier for gene delivery systems, whereas viral vectors have limitations. ML has been used to identify and predict polymers that could serve as efficient gene delivery systems. About 12,000 transfection experiments were conducted using high-throughput techniques. The Logical Analysis of Data (LAD) technique was used to correlate the chemical structures of particular polymers with DNA transfection efficiency. These polymers were characterized using two modeling approaches. These models assisted in identifying specific polymer characteristics that influence transfection efficiency. This research provides a blueprint for designing novel polymers that can deliver genes safely and efficiently.^254^ By incorporating newly discovered polymers, which have been identified using machine learning techniques for their safe and effective gene delivery properties, into the hydrogel matrix, a dual function can be achieved. It would have the ability to not only enable the regulated release of exosomes but also improve their efficiency in delivering genes.

#### 3.4.4 Polymers and Immune System Interaction

Within the field of regenerative medicine, specifically in relation to exosome-loaded hydrogels used for tissue regeneration, it is crucial to comprehend the interaction between polymers and the immune system. To comprehend these interactions based on polymer chemistry, ML has been applied. Researchers examined the immune responses of polymers of meth(acrylate) and meth(acrylamide). They focused on how these polymers affected the phenotypes of human monocyte macrophages. There are proinflammatory M1-like macrophages and anti-inflammatory M2-like macrophages. Using the log ratio of M2 to M1 macrophages and their attachment value, a composite metric was created. After screening 141 types of these polymers, 400 copolymers were prepared and evaluated for their immune responses. The classification of these copolymers was then based on their immunoreactivity. Molecular signature descriptors linked to polymer structure were used to analyze the data, along with the LASSO method to eliminate irrelevant data. Using three ML algorithms, Support vector machine (SVMs), random forest, and multilayer perceptron, models predicting the immunoreactivity of polymers were developed. SVMs, for instance, function by locating the boundary between two data classes. A type of neural network, the multilayer perceptron employs multiple layers of neurons to process data. The accuracy of the models generated by these algorithms was comparable. The most precise model could classify polymers with 80% precision. These models provide insights regarding the design of biocompatible polymers. The identification of specific molecular structures that influence immune responses provides a roadmap for designing safer polymers for medical devices.^255^ The integration of polymers, identified through machine learning analysis, into the hydrogel matrix can augment the biocompatibility and efficacy of hydrogels loaded with exosomes. This method guarantees that the hydrogels not only function as effective carriers for exosome transportation, but also synchronize with the body’s immune mechanisms to facilitate the regenerative procedure.

#### 3.4.5 Bioinks and 3D Bioprinting

The creation of EVs-loaded scaffolds for tissue regeneration is greatly aided by the development of bioinks and 3D bioprinting technologies. The creation of bioinks tailored to specific applications presents considerable difficulties. The viscosity and rheological properties of bioinks must be maintained during extrusion to guarantee the structural integrity of 3D-printed constructs. Printability, which is governed by the ink’s rheological properties, and mechanical stability play a crucial role in determining the ink’s capacity to form a three-dimensional structure. The accurate composition of bioinks is essential when considering EVs-loaded hydrogels. The viscosity and shear behavior of the hydrogel matrix are influenced by the concentration of the formulation, which in turn affects the encapsulation and release of EVs. In addition to the inherent properties of the ink, the adjustable parameters of the 3D printer, such as nozzle specifications, printing pressure, temperatures, and print-head speed, play a crucial role in achieving the desired structure. Adjusting these parameters to create an optimal 3D model can be complex and time-consuming. Inadequate optimization can result in complications such as nozzle clogging and structurally compromised 3D prints. To address these obstacles, there is a growing emphasis on leveraging AI, particularly ML, to optimize the printability of bioink (Figure 5B).^256^ By analyzing experimental data, ML can predict and optimize the properties of biomaterial ink, thereby enhancing printability and reducing fabrication costs.^257^ Paxton et al.,^258^ for instance, demonstrated how a combination of shear-viscosity rheometry and theoretical modeling can improve bioprinting conditions. In a separate study, Lee et al. utilized multiple regression analysis to develop natural bioink formulations, resulting in 3D scaffolds with remarkable shape fidelity and biocompatibility. However, the success of such endeavors depends on the availability of vast data to train ML models.^256^

In conclusion the capacity of ML to rapidly process and learn from massive data sets can significantly accelerate the development of hydrogels loaded with EVs. However, its full potential has yet to be realized. Overcoming obstacles such as lack of standardization across laboratories and countries and ensuring consistency in data collection will be crucial for maximizing the potential of machine learning in this field.

## 4 Applications of EVs-Loaded Hydrogels in Tissue Regeneration

### 4.1 Extracellular Communication and Cancer: Implications for Regenerative Medicine

While this Review primarily focuses on tissue regeneration, it is crucial to recognize the complex role of EVs in extracellular communication, which has significant implications in the fields of oncology and regenerative medicine. The intricate interaction between EVs and cancer cells offers valuable insights into fundamental cellular processes that are essential for comprehending tissue healing and regeneration.

An example of this is the research conducted by Cristina Mas-Bargues et al.,^259^ which emphasizes the ability of EVs obtained from nonsenescent MSCs to revitalize prematurely senescent MSCs. This discovery holds great importance for the field of regenerative medicine, as it indicates a possible method to augment the functionality of stem cells and prolong their lifespan, both of which are crucial elements in the process of tissue regeneration. Moreover, the significance of EVs in the development of cancer, as explored by Angela Maria Bellmunt et al.^262^ and other researchers, highlights the necessity of comprehending cellular microenvironments. This knowledge can be utilized in regenerative medicine to manipulate the microenvironment in order to enhance tissue repair and regeneration, especially in situations where inflammation or immune responses are involved.

**Figure 6:**
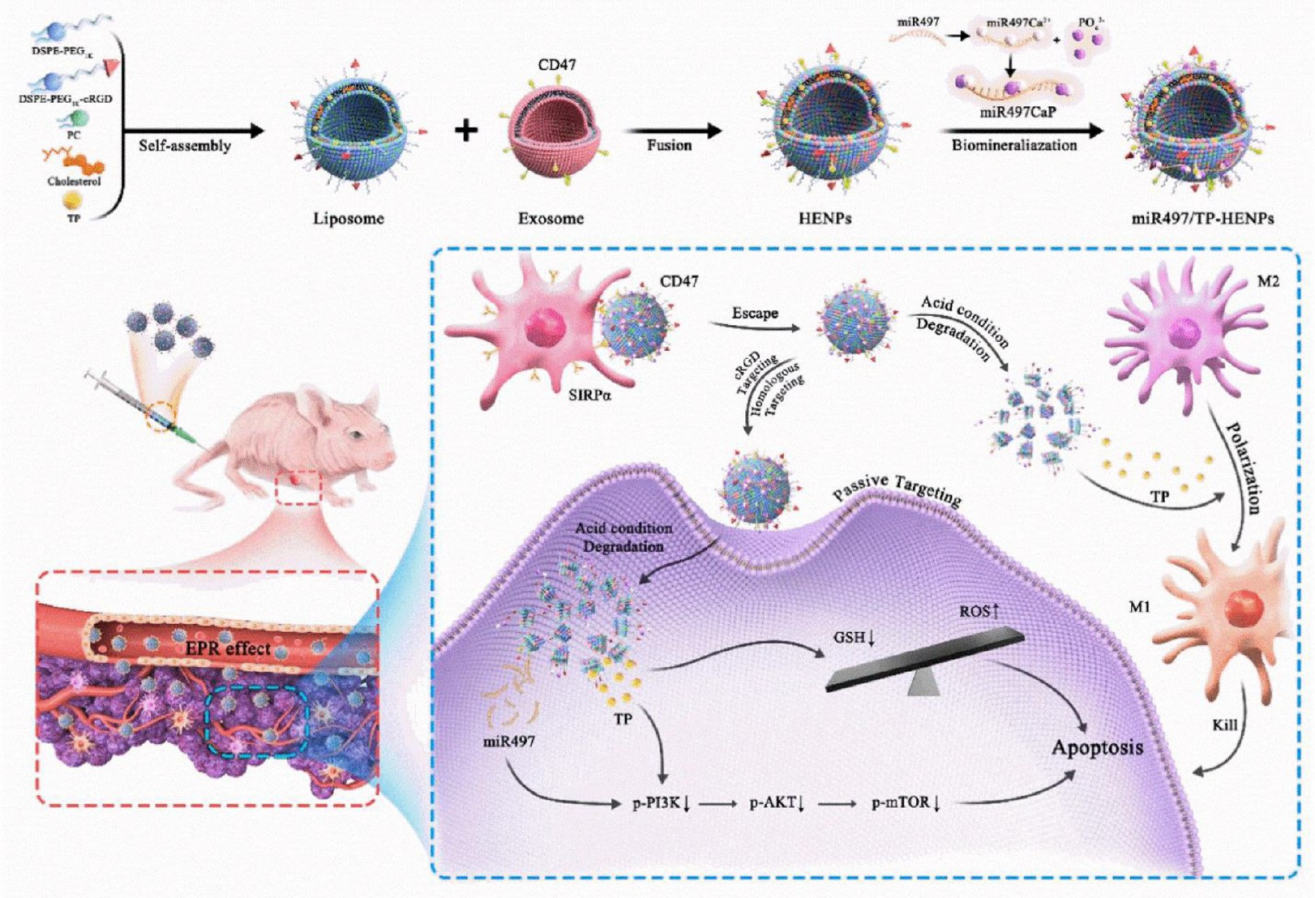
Diagram illustrating the formation process and action mechanism of miR497/TP-HENPs. miR497/TP-HENPs were produced through membrane fusion and biomineralization techniques. First, liposomes were synthesized by assembling phosphatidylcholine (PC), cholesterol, and TP-encapsulated DSPE-PEG1k-cRGD. The membranes of the liposomes and exosomes were then fused together. Finally, CaP adsorbs miR497 onto the nanoparticles’ surface. Reprinted with permission from ref (263). Copyright 2022 Springer Nature.

The study on addressing chemoresistance in ovarian cancer through the utilization of a bioinspired hybrid nanoplatform also offers a significant viewpoint (Figure 6). To accomplish this, the bioinspired hybrid nanoplatform, namely, miR497/TP-HENPs was formed by fusing tumor exosomes expressing CD47 with liposomes modified with cRGD, forming hybrid nanoparticles. These nanoparticles were designed to simultaneously deliver miR497 and TP. These nanoparticles were efficiently absorbed by tumor cells *in vitro*, resulting in a significant increase in tumor cell apoptosis. *In vivo*, the hybrid nanoparticles exhibited a remarkable affinity for tumor regions, resulting in observable anticancer activity with no adverse side effects. They facilitated the dephosphorylation of the hyperactivated Overactivation of the phosphatidylinositol 3-kinase/protein kinase B/mammalian target of rapamycin (PI3K/AKT/mTOR) signaling pathway, increased reactive oxygen species (ROS) production, and facilitated the conversion of M2 macrophages to M1 macrophages.^263^ These findings suggest a promising strategy for combating cisplatin-resistant ovarian cancer and may provide a treatment option for other cisplatin-resistant tumor types. The principles employed in targeting and manipulating cellular pathways for cancer treatment can be modified to facilitate tissue regeneration, particularly in the development of targeted delivery systems for regenerative therapies.^263^

Furthermore, the innovative approach of utilizing a synthetic EVs-based hydrogel to treat advanced ovarian cancer demonstrates the capability of EVs-loaded hydrogels to precisely target particular cell types.^264^ This strategy can be replicated in regenerative medicine to selectively focus on particular groups of cells or to regulate immune reactions during the regenerative procedure.

To summarize, although these studies are mainly used in oncology, the fundamental concepts of cellular communication, manipulation of microenvironment, and precise delivery are also applicable to tissue regeneration. Through comprehending these mechanisms, we can cultivate regenerative therapies that are more potent by utilizing the capabilities of EVs and hydrogels.

### 4.2 Wound Healing

Wound healing is a complex biological process that can be triggered by surgery, trauma, burns, or diabetes, resulting in a mass of nonfunctional fibrotic tissue. In the field of skin regeneration, regenerative medicine, specifically stem cell therapies, has garnered significant attention in recent years. Due to their capacity to secrete cytokines and growth factors that promote wound healing, mesenchymal stem cells are utilized in wound therapy.^266^ Mesenchymal stem cells (MSCs) secrete factors with mitogenic, pro-angiogenic, anti-inflammatory, and antifibrotic properties, which expedite wound healing collectively.^267^ As a result, stem cell secretomes have emerged as an innovative and promising treatment for delayed wound healing. Their application can address a number of issues associated with living cells, including immunological compatibility, tumor development, genetic diversity, and infection transmission.^268^

EVs secreted by these stem cells, according to recent research, may contribute to their paracrine effect.^269^ In regenerative medicine, these EVs, which can secrete large amounts of growth factors, cytokines, and other paracrine factors, have demonstrated promising therapeutic effects. Nonetheless, direct stem cell therapy presents significant obstacles, such as the immune system’s response and the release of inflammatory factors.^270,271^ Several potential safety concerns associated with whole-cell transplantation may be mitigated by cell-free therapeutic strategies in regenerative medicine. Numerous biomolecules contribute to angiogenesis, cell growth and proliferation, migration, tissue reorganization, and ECM remodeling during the wound healing process.^272^ EVs may reach cells involved in skin regeneration, including keratinocytes, endothelial cells, and fibroblasts, and regulate signaling pathways within these target cells.^273^

EVs may have the ability to accelerate and improve various stages of the wound healing process by transporting a vast array of biological cargoes. These cargoes, which include miRNAs, mRNAs, and proteins, are capable of modulating signaling pathways within target cells. By transferring Wnt-4, EVs derived from human umbilical cord MSCs have been shown to stimulate -catenin activation and promote angiogenesis in a rat skin burn model. Once these EVs reach their target cells, they activate signaling pathways that promote the expression of several growth factors involved in wound healing, including STAT3, IL-6, SDF-1, IGF-1, and HGF.^273^

**Figure 7:**
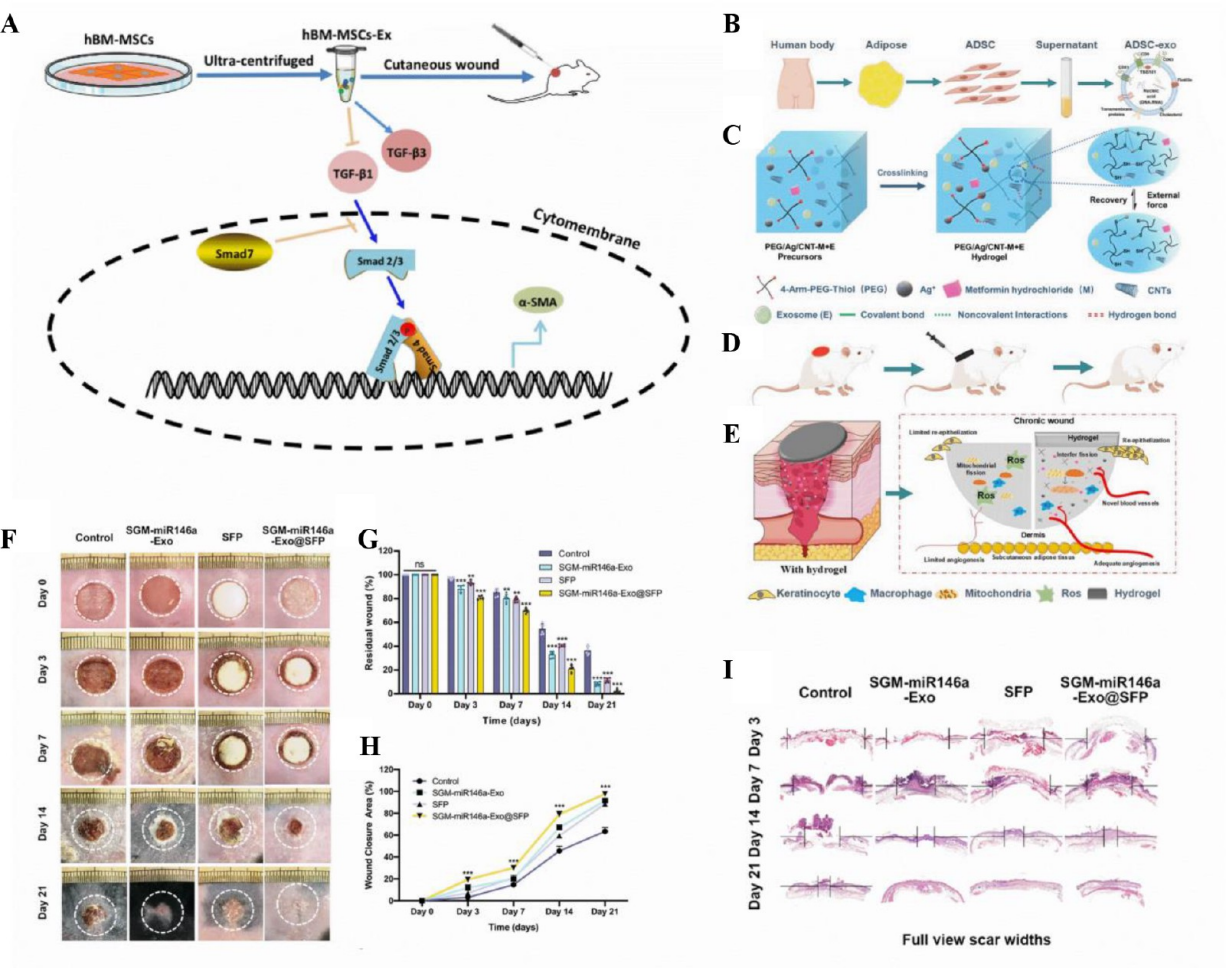
(A) By modulating the TGF-/Smad signaling pathway, hBM-MSC-Ex promotes skin wound healing. Specifically, hBM-MSC-Ex inhibits TGF-1 expression while enhancing TGF-3 expression. Variants of TGF- and activins initiate intracellular signaling via Smad-2/3 transcription factors. When Smad-2 and Smad-3 are phosphorylated, they bind to Smad-4 and stimulate the transcription and expression of -SMA. In addition, the interaction of the TGF- superfamily with cell surface receptors activates the inhibitory Smad7. Reprinted with permission from ref (276). Copyright 2020 Springer Nature. (B) Schematic representation of the preparation and application of PEG/Ag/CNT-M + E hydrogel for chronic wounds. ADSCs and ADSC-Exos are isolated. (C) The hydrogel PEG/Ag/CNT-M + E synthesis diagram. (D, E) The PEG/Ag/CNT-M + E hydrogel was applied to a diabetic mouse, where it promoted wound healing by stimulating cell proliferation and angiogenesis and reduced the level of reactive oxygen species (ROS) by inhibiting mitochondrial fission. E) Schematic representation of the experimental procedures. Reprinted with permission from ref (278). Copyright 2023 Elsevier. (F) The gross appearance of wounds on days 0, 4, 7, 14, and 21. The white circles indicate the initial wound sites. G) Statistical analysis of the wound area remaining in each group (*n* = 6 per group). (H) Statistical analysis of the area of wound closure (*n* = 6 per group). (I) Illustrations of scar widths stained with HE. Reprinted with permission from ref (279). Copyright 2023 Springer Nature.

Numerous studies have used MSCs derived from umbilical cords to demonstrate their efficacy in promoting wound healing. These studies emphasize their enhanced antiscarring properties, ability to inhibit TGF-SMAD signaling, and role in reducing collagen deposition.^274^ Experiments conducted *in vivo* with sEVs derived from human adipose MSCs^275^ and bone marrow MSCs^276^ demonstrated similar positive effects on skin regeneration and wound healing (Figure 7A). These results demonstrate the capacity of sEVs to promote wound healing by activating multiple signaling pathways and molecular events within injured cells. In addition, it is known that MSCs-sEVs possess anti-inflammatory and immunomodulatory properties, highlighting their potential as a promising cell-free approach for regenerative medicine.^277^

Current research indicates, however, that the selection of effective delivery methods is essential for sEVs-based therapies. As these materials can encapsulate and regulate the release of sEVs at the wound bed, engineered polymer-based biomaterials represent a promising avenue. Evaluations *in vitro* and *in vivo* indicate that the combination of biodegradable porous hydrogels and sEVs as wound dressings amplifies the efficacy of sEVs in promoting wound healing.^14^ In a recent research conducted by Yue Zhang et al.^278^ dual-loaded hydrogels with a variety of advantageous properties such adhesiveness to tissues, antioxidant, self-healing, and having electrical conductivity was developed. To accomplish this, they utilized a 4-armed SH-PEG compound that cross-links with Ag+, thereby effectively minimizing the deterioration of the loaded substances (Figure 7B–E). In addition, they investigated the intricate workings of these hydrogels in order to gain a deeper understanding of the mechanisms that promote wound healing. To further improve the hydrogels’ functionality, multiwalled carbon nanotubes were incorporated, which are renowned for their exceptional conductivity. These nanotubes form hydrogen bonds with the thiol group, creating a stable 3D structure that facilitates sEVs and metformin loading. The outcomes of their experiments with a diabetic wound model have been encouraging. The PEG/Ag/CNT-M + E hydrogel has demonstrated the potential to accelerate wound healing by promoting cell proliferation and angiogenesis, as well as by reducing peritraumatic inflammation and vascular injury. This can be attributed to the hydrogel’s unique mechanism, which involves inhibiting mitochondrial fission to reduce the level of ROS. As a result, homeostasis of F-actin is maintained and microvascular dysfunction is mitigated.

In another study by Qiankun Li et al.^279^ a novel and effective miRNA delivery system based on specially engineered exosomes and silk fibroin (SF) scaffolds to accelerate the healing of diabetic wounds was developed. Silk fibroin binding peptide (SFBP)-Gluc-MS2 (SGM) and pac-miR146a-pac fusion proteins were constructed and then introduced into human placenta-derived mesenchymal stem cells (PMSCs). The SGM protein was then incorporated into the plasma or endosomal membrane of PMSCs. Due to the specific binding of the phage MS2 capsid protein and the pac site, when exosomes are released from these membranes, miR146a is efficiently encapsulated within the exosomes. During the biogenesis of the exosomes, the SFBP, which targets SF, is also expressed in the exosomal membrane, enhancing the binding rate of SGM-miR146a-Exos to the SF patch (SFP). This newly developed system could increase the efficacy of engineered exosomes carrying the target miRNA via MS2, allowing them to play a more effective regulatory role. In addition, when bound to SF, exosomes with an affinity for silk fibroin could be better preserved and have longer-lasting therapeutic effects. Compared to untreated, SGM-miR146a-Exo-only treated, and SFP-only treated groups, SGM-miR146a-Exo@SFP exhibited superior wound healing therapeutic effects in diabetic patients (Figure 7F–I).

Despite the progress made in EVs research, there are still obstacles to overcome. The difficulty of separating and purifying exosomes is one of the primary obstacles, necessitating continuous advancements in separation technologies to increase the yield and purity of isolated exosomes. In addition, the mechanisms of exosomes in the diabetic wound healing process are not fully understood. EVs remain an attractive therapeutic target for diabetic wounds, however, due to their specific bioactive substances.

### 4.3 Bone Regeneration

Bone defects, fractures, tumors, and periodontitis cannot be effectively treated without the regeneration process. When bone is damaged, a series of reactions ensue, including inflammation, the activation of repair mechanisms, and various stages of tissue remodeling. During the inflammatory phase, which is characterized by bleeding from the fracture and damage to the surrounding soft tissue, inflammatory cytokines are released. Following this, mesenchymal progenitor cells multiply and differentiate into osteoblasts and chondrocytes at the site of injury. Additionally, key factors influence the differentiation of these mesenchymal stem cells into chondrocytes or osteoblasts.^280^

sEVs, which are secreted by diverse bone tissue cells including MSCs, osteoblasts, osteoclasts, osteoclast precursors, osteocytes, bone marrow adipocytes, and bone marrow stromal cells, play a pivotal role in bone regeneration and remodeling via a variety of mechanisms. These sEVs induce osteogenesis and osteogenic differentiation in MSCs by directly transferring specific active molecules, specifically miRNAs, and then regulating the expression of relevant gene networks in the target cells.^214^ sEVs derived from BMSCs have been shown to increase osteoblast proliferation via the MAPK signaling pathway, demonstrating their efficacy in treating osteoporosis. In a study conducted by P. Zhao et al.^281^ the MSCs-exos was extracted from rat bone marrow, following the identification of the MSCs surface antigen using flow cytometry. Positive identification of surface antigens on MSCs indicated a robust capacity for multidirectional differentiation. It was found that exosomes promoted the proliferation of hFOB 1.19 cells. In addition, the mRNA and protein expressions of GLUT3 in the cells increased, and the cell cycle was also stimulated. An increase in the expression of MAPK signaling pathway-related proteins was also observed. Experiments on hFOB 1.19 revealed that MSCs-exos could stimulate its growth and cell cycle. However, these effects were reversed by inhibiting p-JNK. This suggests that MSCs-exos plays a vital role in promoting cell growth and the cell cycle, which may provide new insights for future research.

**Figure 8:**
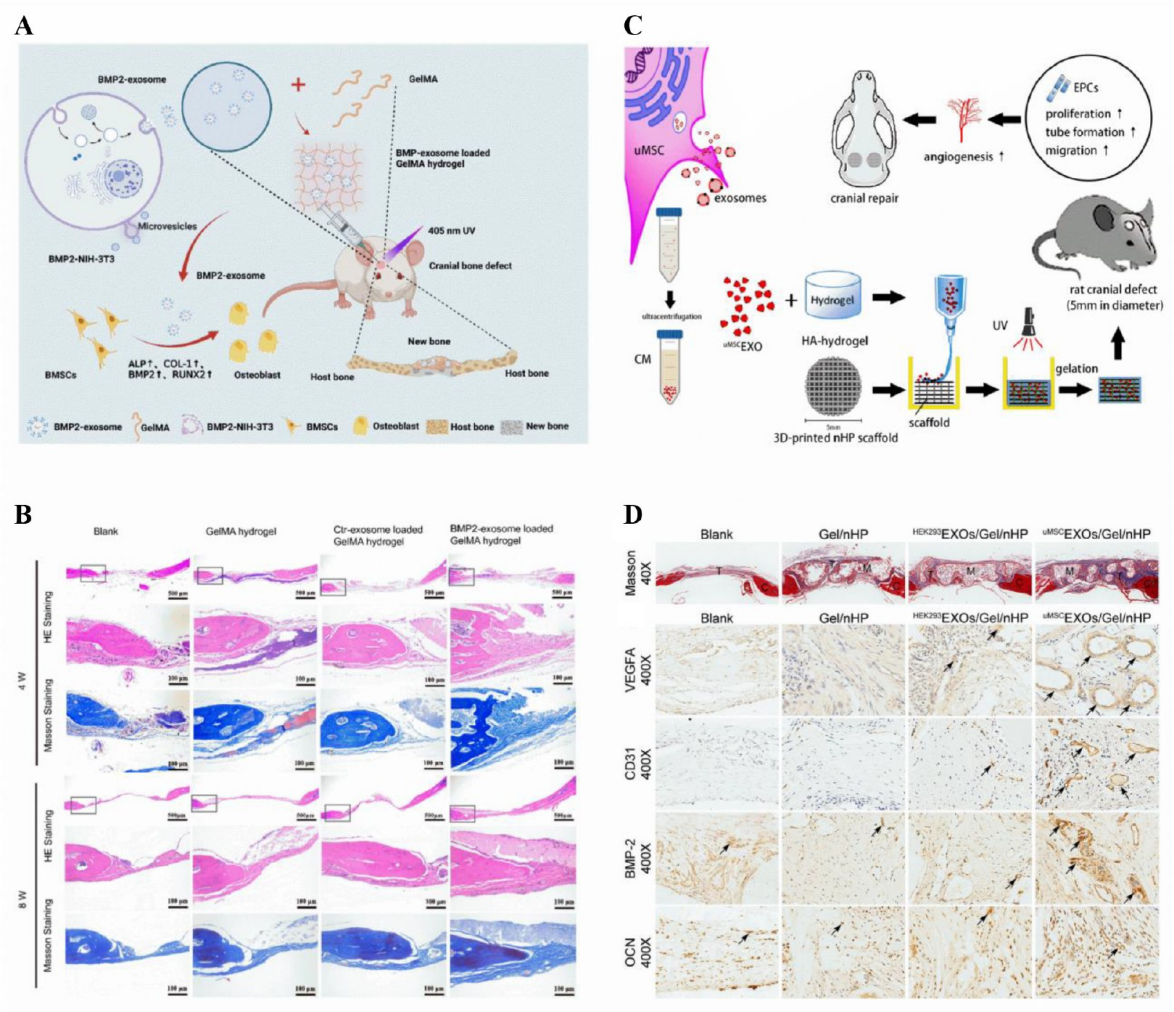
(A) Schematic illustration of the engineered process and continuous release of BMP2-enhanced exosomes embedded in a biodegradable hydrogel for cranial defect healing. (B) Images of 6 histological staining of cranial defect areas in the Blank, GelMA hydrogel, Ctr-exosome loaded GelMA hydrogel, and BMP2-exosome loaded GelMA hydrogel groups following 4 and 8 weeks of treatment.5. Reprinted with permission from ref (282). Copyright 2023 Elsevier. (C) Schematic flow diagram of uMSCEXOs combined with the HA-Gel and the nHP scaffold to enhance angiogenesis and repair the critical-sized cranial defect in rats. (D) Images of Masson’s trichrome staining (40) depicting a sagittal view of the cranial defect region. Reprinted with permission from ref (283). Copyright 2021 American Chemical Society.

Combining gene engineering and ultracentrifugation techniques, Jinru Sun et al.^282^ developed a novel strategy for engineering bone-functional exosomes, namely BMP2-overexpressed exosomes (Figure 8A). These BMP2-overexpressed exosomes were encapsulated in a biodegradable GelMA hydrogel to ensure sustained delivery at bone defect sites. The BMP2-overexpressed exosomes carried by the biodegradable hydrogel significantly enhanced the osteogenic differentiation of BMSCs *in vitro*. In addition, they improved *in vivo* bone regeneration at cranial defect sites (Figure 8B). In another study,^283^ exosomes derived from umbilical mesenchymal stem cells (uMSCEXOs) were encapsulated in a hyaluronic acid hydrogel (HA-Gel) and integrated with nanohydroxyapatite/poly-ε-caprolactone (nHP) scaffold to repair cranial defects in rats (Figure 8C). Imaging and histological analyses revealed that the uMSCEXOs/Gel/nHP composites significantly enhanced bone regeneration *in vivo*, with uMSCEXOs playing a key role in this process (Figure 8D). Moreover, the *in vitro* experiments demonstrated that uMSCEXOs stimulated the proliferation, migration, and angiogenic differentiation of endothelial progenitor cells (EPCs), but had no significant effect on the osteogenic differentiation of BMSCs. Importantly, the mechanistic research identified exosomal miR-21 as a possible intercellular messenger that stimulates angiogenesis by upregulating the NOTCH1/DLL4 pathway. These findings reveal a promising exosome-based strategy for repairing large bone defects via enhanced angiogenesis, which may be regulated by the miR-21/NOTCH1/DLL4 signaling axis. This strategy could pave the way for novel regenerative medicine treatments.

Despite the fact that the role of sEVs in regulating osteogenesis during bone repair is well-established, their therapeutic efficacy may be affected by underlying disorders in the donors. BMSC-derived exosomes derived from rat models with type 1 diabetes and those derived from normal rats both promoted the osteogenic differentiation of BMSCs and enhanced the angiogenic activity of endothelial cells. However, the therapeutic effects of diabetic rats’ exosomes were weaker than those of normal rats. These results suggest that autologous exosome transplantation may not be an appropriate therapeutic strategy for donors with chronic underlying diseases.^284^

### 4.4 Cardiac Repair

As the central component of the circulatory system, the heart connects blood vessels to ensure adequate blood supply to all organs. Cardiovascular disease, the leading cause of death on a global scale, causes damage to blood vessels, valves, and myocardial tissue. Some patients never regain full cardiovascular functionality despite extensive efforts and a variety of treatments.^285^ Recent advances in tissue engineering, however, have shown promise for enhancing cardiovascular function by replacing damaged tissue with endogenous repair. Particularly, cardiac tissue engineering has the potential to meet the demand for heart transplants in the near future. Myocardial infarction, a condition caused by the occlusion of coronary arteries, results in a necrotic area of heart tissue and is potentially fatal. Innovative treatment techniques, such as the use of exosomes, can halt the progression of an infarction and improve its area.^286,287^

**Figure 9:**
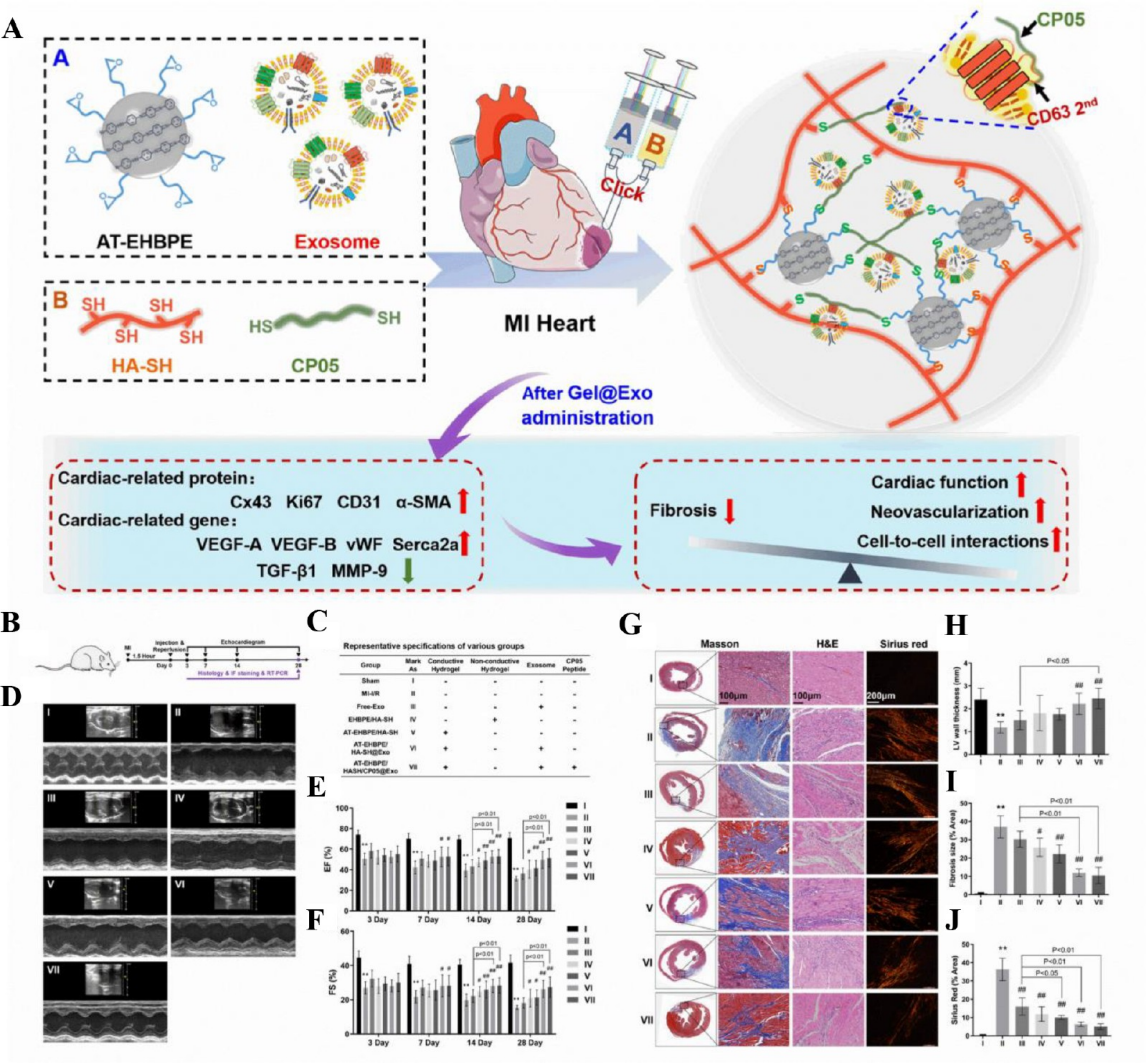
(A) Schematic illustration of the injectable conductive hydrogel that anchors hUC-MSCs-exos for the restoration of cardiac function following Myocardial Infarction-Ischaemia/Reperfusion (MI-I/R). (Blue A) AT-EHBPE precursor solution with exosomes. (Red B) Solution containing HA-SH and the CP05 peptide as precursors. (B) Scheme for animal experimentation. (C) Specifications representative of various groups (+ represents containing and - represents not containing). (D) Representative ECHO images at 28 days post-treatment for the various groups (I, Sham; II, MI-I/R model; III, Free-Exo; IV, EHBPE/HA-SH; V, AT-EHBPE/HA-SH; VI, AT-EHBPE/HA-SH@Exo; and VII, AT-EHBPE/HA-SH/CP05@Exo). (E) Quantitative statistics of EF and (F) FS assessed by ECHO on 3, 7, 14, and 28 days after various treatments (*n* = 6–7; **P < 0.01 versus Sham; #*P* < 0.05, and ##*P* < 0.01 versus MI-I/R model). (G) Representative images of Masson’s-trichrome staining (left: 5 mm, right: 100 μm), HE staining (100 μm), and Sirius red staining (200 μm). (I, Sham; II, MI-I/R; III, Free-Exo; IV, EHBPE/HA-SH; V, AT-EHBPE/HA-SH; VI, AT-EHBPE/HA-SH@Exo; and VII, AT-EHBPE/HA-SH/CP05@Exo). H) Quantitative measurements of fibrosis size and (I) LV wall thickness of rat hearts. (J) Analysis quantitative of Sirius Red. (*n* = 3; **P < 0.01 vs Sham; #*P* < 0.05 and ##*P* < 0.01 vs MI-I/R model). Reprinted with permission from ref (288). Copyright 2021 American Chemical Society.

Notably, the use of sEVs derived from human umbilical cord (hUC-MSCs) and induced pluripotent stem cell-derived cardiomyocytes, encapsulated within an electroconductive hydrogel and injected into a rat model of myocardial infarction, has produced encouraging results. It has been demonstrated that these exosomes encapsulated in functional peptide hydrogels reduced myocardial fibrosis and infarcted area size and promoted cardiac repair (Figure 9). Echocardiographic examinations have revealed an increase in ejection fraction, resulting in an increase in cardiac output and a decrease in arrhythmia caused by myocardial damage (Figure 9D). In addition, the use of hUC-MSCs-sEVs has been linked to the expression of cardiac-related proteins (Cx43, Ki67, CD31, and α-SMA) and remarkably upregulating genes (VEGF-A, VEGF-B, vWF, TGF-β1, MMP-9, and Serca2a) in damaged myocardial tissue. These results suggested that sEVs derived from endothelial progenitor cells can effectively aid in heart tissue repair via slow release when loaded into scaffolds.^288^

According to research,^289^ miRNAs play a crucial role in the onset and progression of myocardial infarctions, making them indispensable for the prevention and treatment of this condition. For instance, miR-30e and miR-92a can regulate lipid metabolism to prevent atherosclerosis, a condition that can lead to vascular stenosis and myocardial infarction. Consequently, these miRNAs have the potential to prevent myocardial infarctions to some extent. By regulating autophagy, miRNAs can also promote the survival of myocardial cells and maintain cellular metabolic balance. For example, miR-210 can regulate vascular endothelial growth factor (VEGF) and hepatocyte growth factor (HGF), thereby stimulating the proliferation and migration of endothelial cells, promoting the formation of cardiac blood vessels, and enhancing ventricular remodeling. However, the degradation of miRNA *in vivo* restricts its application in cardiovascular disease.^289^

Multiple recent studies,^290,291^ however, have demonstrated that exosomes possess cardioprotective properties. sEVs derived from hemin-pretreated MSCs (Hemin-MSCs-sEVs) may transport cardioprotective miRNAs to cardiomyocytes, according to a recent research.^292^ Notably, the levels of miR-183–5p are significantly increased in Hemin-MSCs-sEVs compared to MSCs-sEVs. Moreover, Hemin-MSCs-EVs inhibited SD/H-induced cardiomyocyte senescence in part by delivering miR-183–5p to recipient cardiomyocytes via HMGB1/ERK pathway regulation. Furthermore, the knockdown of miR-183–5p diminished the cardioprotective effects of Hemin-MSCs-sEVs in a mouse model of MI. In another study,^293^ the antiapoptotic effect of exosomes secreted from induced pluripotent stem cells and their differentiated cardiomyocytes (iCMs) in a mouse model of acute myocardial ischemia/reperfusion has been confirmed. After myocardial infarction, mice treated with exosomes derived from iCMs (iCM-sEVs) exhibited significant cardiac improvement, with significantly reduced apoptosis and fibrosis. Hypoxia and exosome biogenesis inhibition significantly reduced *in vitro* iCMs apoptosis, which was restored by treatment with iCM-sEVs or rapamycin. *In vivo* and *in vitro*, autophagosome production and autophagy flux were upregulated in iCM-sEVs groups. The cardioprotective effects of miRNA-144 (miR-144) found in BMSCs-sEVs was also investigated. The researchers discovered that H9C2 cells that absorb these exosomes are protected from apoptosis under hypoxic conditions. This protective effect is diminished by inhibiting miR-144. The study concluded that MSC-sEVs can inhibit cell apoptotic injury under hypoxic conditions by delivering the PTEN/AKT pathway-targeting miR-144. This suggests that MSCs-sEVs may have therapeutic applications in the treatment of ischemic conditions.^291^

### 4.5 Neurological Diseases

MSCs-sEVs have demonstrated significant therapeutic potential for neurodegenerative disorders.^294^ When loaded into hydrogels, these nanoscale vesicles can provide a sustained release mechanism, thereby improving their therapeutic efficacy in neurological applications.^295^ The therapeutic potential of sEVs derived from BMSCs was studied by investigating the role of miRNAs in ischemic stroke (IS) progression. It was hypothesized that sEVs derived from BMSCs containing overexpressed miR-138–5p could protect astrocytes against IS by targeting lipocalin 2 (LCN2). LCN2 was discovered to be highly expressed in IS and a target gene of miR-138–5p. Overexpression of miR-138–5p stimulated the proliferation and inhibited the apoptosis of oxygen-glucose-deprived astrocytes, thereby reducing the expression of inflammatory factors by down-regulating LCN2. Importantly, BMSCs delivered miR-138–5p to astrocytes via exosomes, and this miR-138–5p mitigated neuron damage in IS mice. The results suggest that BMSC-derived exosomal miR-138–5p could reduce neurological impairment by promoting proliferation and inhibiting inflammatory responses of astrocytes following IS by targeting LCN2, thereby providing a potential novel target for treating IS.^296^

It is also known that MSCs-sEVs contain specific miRNAs that promote neuroregeneration. For example, miR-133b and the miR-17–92 cluster, which are present in MSCs-sEVs, have been linked to neurite remodeling, oligodendrogenesis, neurogenesis, and neural remodeling, thereby promoting efficient recovery in stroke models.^297^

The therapeutic potential of MSCs-sEVs also extends to models of traumatic brain injury (TBI). In a recent study by Yunfei Chen et al.^298^ collagen, silk fibroin, and exosomes derived from hypoxia-pretreated hUC-MSCs were used to create 3D-printed scaffolds. These scaffolds, known as 3D-CS-HMExos, exhibited excellent biocompatibility and biodegradability, making them a promising treatment option for TBI. The team also created hypoxia-preconditioned exosomes from hUC-MSCs, which enhanced neuroregeneration following TBI. The 3D-CS-HMExos scaffolds enhanced with hypoxia-induced exosomes promoted neuroregeneration and angiogenesis, reduced nerve cell apoptosis, and modulated inflammation when implanted into the brains of injured beagle dogs. This resulted in enhanced motor function recovery in the dogs with TBI. The findings suggest that 3D-CS-HMExos implants may be a viable option for repairing TBI-related cavities. In another research,^299^ the researchers hypothesized that these exosomes would inhibit apoptosis, reduce neuroinflammation, and stimulate neurogenesis following TBI. Using a rat model, the researchers discovered that treatment with exosomes derived from hUC-MSCs significantly improved sensorimotor function and spatial learning following TBI. Inhibiting the NF-κB signaling pathway, these exosomes reduced the expression of proinflammatory cytokines. In the injured cortex of TBI rats, they also observed a decrease in neuronal apoptosis, decreased inflammation, and enhanced neuron regeneration. These findings suggest that exosomes derived from hUC-MSCs could be a promising treatment for TBI.

Electroconductive hydrogels have been used extensively to stimulate nerve regeneration and restore locomotor function after PNI, especially in diabetic patients. In this context, the use of nerve guidance conduits (NGCs) constructed from graphene oxide (GO) and GelMA for the treatment of peripheral nerve injuries was investigated. The NGCs, which are chemically reduced to produce reduced (GO/GelMA) (r(GO/GelMA)), possess excellent electrical conductivity, flexibility, mechanical stability, and permeability. These NGCs significantly promoted neuritogenesis in PC12 neuronal cells, according to *in vitro* studies. *In vivo* studies utilizing a rat sciatic nerve injury model revealed that r(GO/GelMA) NGCs significantly improved peripheral nerve regeneration, as evidenced by enhanced muscle weight gain, electro-conduction velocity, and sciatic nerve function index. Preclinical studies indicate that these electrically conductive hydrogel NGCs show promise as functional conduits for enhanced nerve regeneration.^300^

In conclusion, the incorporation of sEVs into hydrogel systems represents a promising treatment strategy for neurological disorders. These exosome-loaded hydrogels can provide a sustained release of therapeutic agents, thereby enhancing their potential to alleviate neurodegenerative disease symptoms and promote recovery.

### 4.6 Kidney Diseases

EVs, specifically sEVs, have become a hopeful option for regenerative medicine as they possess the natural capability to enhance intercellular communication and regulate cellular functions. Their significance is progressively acknowledged in the realm of kidney diseases, wherein they present potential therapeutic approaches for conditions like acute kidney injury (AKI). Stem cells-derived EVs, particularly those derived from MSCs, contain a wide range of bioactive molecules such as miRNAs that have a significant impact on the regeneration and repair of renal tissues. sEVs derived from human BMSCs (hBMSC-sEVs) have been shown to promote the proliferation of tubular cells in a model of glycerol-induced AKI in severe combined immunodeficiency disease (SCID) mice, resulting in the recovery of renal function via the transfer of specific miRNAs. A recent study^254^ demonstrated that hBMSC-sEVs can protect against hypoxia/reoxygenation (H/R) injury. These sEVs are abundant in the miRNA miR-199a-3p, which, when introduced to renal cells, decreases their susceptibility to H/R injury and apoptosis. The study also discovered that hBMSC-sEVs treatment inhibits the expression of semaphorin 3A (Sema3A) and activates the AKT and ERK pathways, which are essential for cell survival. In contrast, inhibition of miR-199a-3p restores Sema3A expression and inhibits the activation of the AKT and ERK pathways. Injecting hBMSC-sEVs into mice with ischemia/reperfusion (I/R) injury resulted in functional recovery, histologic protection, and decreased levels of cleaved caspase-3 and Sema3A, according to *in vivo* experiments. The delivery of miR-199a-3p to renal cells, downregulation of Sema3A expression, and activation of the AKT and ERK pathways suggest that hBMSC-sEVs could be a novel therapeutic approach for kidney diseases.

**Figure 10:**
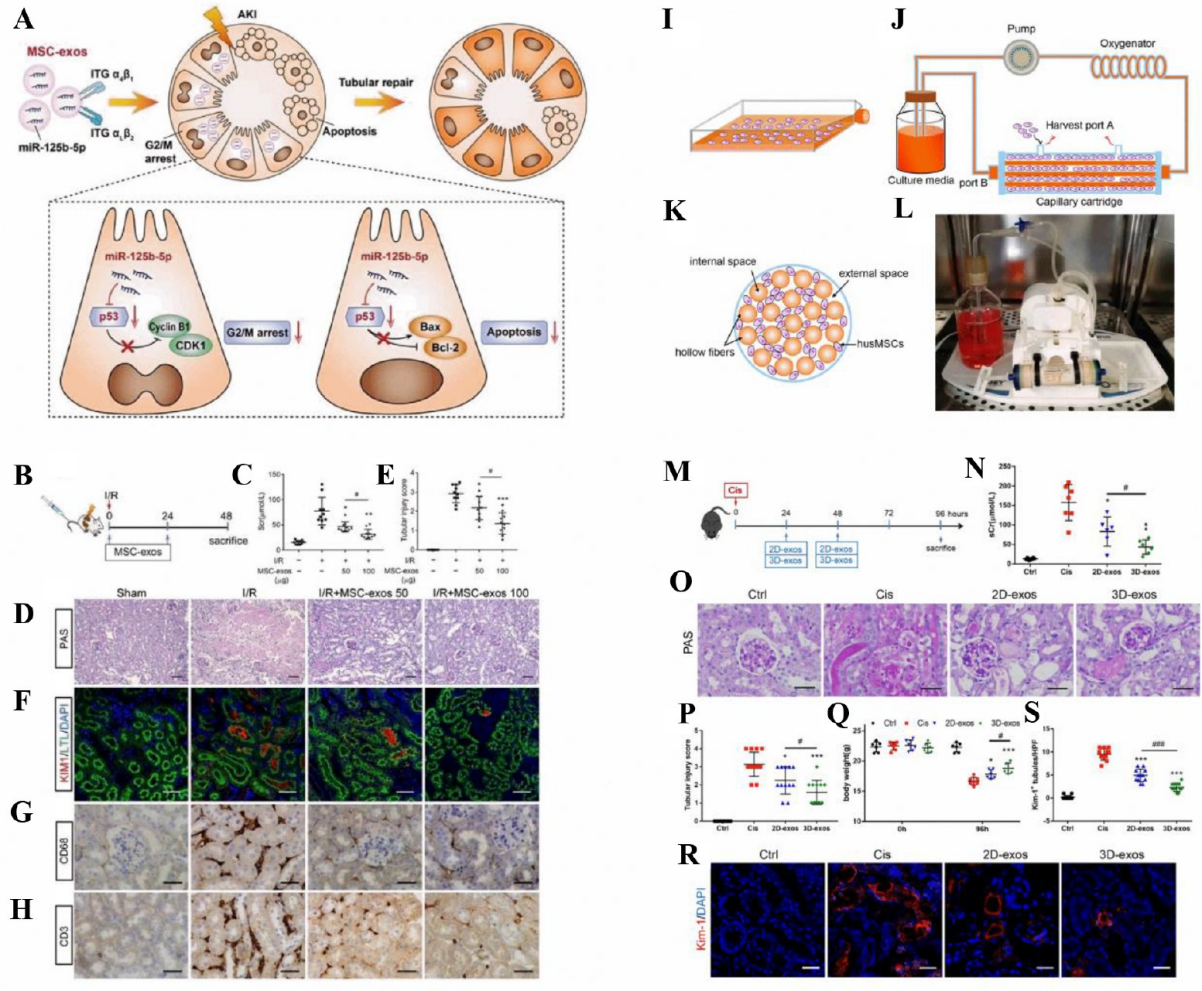
(A) A schematic depiction of the mechanism by which MSC-exos treat ischemic AKI. In ischemic AKI, TEC damage may cause cell cycle arrest in the G2/M phase and apoptosis. Due to VLA-4 and LFA-1-mediated adhesive interactions, MSCs-exos targeted injured kidney, particularly the proximal tubules. In addition, miR-125b-5p was enriched in MSCs-exos and inhibited the expression of p53, which not only up-regulated CDK1 and Cyclin B1 to rescue tubular G2/M arrest but also modulated Bcl-2 and Bax to inhibit TECs apoptosis. (B) Diagrammatic representation of the experimental design. Mice were administered MSCs-exos (50 or 100 μg) or PBS at 0 and 24 h after inducing renal I/R injury and were euthanized at 48 h. (C) Effects of MSCs-exos on serum creatinine dependent on dosage (*n* = 10). (D) PAS-stained images of representative renal I/R injuries. 50 μm for scale bars. (E) The semiquantification of tubular injury (*n* = 10). (F) Exemplary confocal images of KIM-1+-positive tubules. Scale bars, 25 μm. (G) Images illustrative of CD68+ macrophages in the tubulointerstitium. 50 μm for scale bars. (H) Examples of CD3+ T cells within the tubulointerstitium. 50 μm for scale bars. Reprinted with permission from ref (302). Copyright 2023 Springer Nature. (I) The diagram of a standard 2D flask. J) The general schematic of the 3D culture system based on hollow fiber bioreactors. The system included a pulsatile perfusion pump, an oxygenator, a cartridge with thousands of hollow fibers, a bottle of culture medium, and a connecting tube. (K) A schematic representation of a cross-sectional view of the bioreactor. (L) The image of the 3D cultivation system. (M) Diagrammatic representation of the experimental design. Mice were administered PBS, 2D-exos (100 μg), or 3D-exos (100 μg) at 24 and 48 h after cisplatin injection and sacrificed 96 h after disease induction. (N) Serum creatinine effects of 2D-exos and 3D-exos (*n* = 6–7). (O) Illustrations of PAS staining of the renal cortex. Scale bar is 50 μm. (P) Quantification of tubular damage using PAS staining (*n* = 6–7). (Q) Body weight effects of 2D-exos and 3D-exos (*n* = 6–7). (R) Typical confocal images of kidney injury molecular-1 (Kim-1) in tubules. Scale bar is 25 μm. (S) The number of Kim-1+ tubules in each HPF (*n* = 6). Reprinted with permission from ref (303). Copyright 2020 Springer Nature.

In another study^302^ the therapeutic potential of sEVs derived from hUC-MSCs for AKI was investigated (Figure 10A–H). sEVs were discovered to efficiently target the ischemic kidney, accumulating in the proximal tubules. In a dose-dependent manner, they ameliorated murine ischemic AKI and decreased renal tubule injury. sEVs also significantly reduced tubular epithelial cell cycle arrest and apoptosis. This effect was attributable to the high concentration of miR-125b-5p in the sEVs, which inhibited the protein expression of p53 in the cells, resulting in the up-regulation of CDK1 and Cyclin B1 and the modulation of Bcl-2 and Bax. Inhibiting miR-125b-5p could diminish the sEVs’ protective effects. These sEVs ameliorate ischemic AKI and promote tubular repair by targeting cell cycle arrest and apoptosis of tubular epithelial cells via the miR-125b-5p/p53 pathway, according to the study’s conclusion.

Another research^303^ aimed to develop a new method for producing MSCs-sEVs using a 3D culture system in a hollow fiber bioreactor, as well as to evaluate their therapeutic efficacy on AKI (Figure 10I–S). Compared to the conventional two-dimensional (2D) system, the 3D culture system significantly increased MSCs-sEVs production by up to 19.4 times. In a mouse model of cisplatin-induced AKI, both 2D and 3D cultured sEVs significantly improved renal function and reduced inflammation, with 3D-sEVs being more effective. The study concludes that the 3D culture system provides an effective method for the continuous production of MSCs-sEVs, which have increased therapeutic potential for treating AKI.

MSCs-sEVs may also induce angiogenesis in kidney injury models, as postulated. Recent research^304^ indicates that kidney-derived MSCs-sEVs promote angiogenesis in renal tissue. The therapeutic effects of sEVs, which are vesicles secreted by cells and contain various biologically active components, in a pig model of I/R-AKI was investigated. The pig model was administered hUC-MSCs-sEVs. The results demonstrated that a single dose of these sEVs significantly enhanced kidney function, decreased cell death, and reduced inflammation. sEVs also promoted renal tubular cell proliferation, angiogenesis, and upregulation of renoprotective molecules. These results suggest that hUC-MSCs-sEVs could be utilized as a biological treatment for acute kidney injury.

### 4.7 Repair of Cartilage Defects

Due to the cartilage’s limited self-regenerative capacity, articular cartilage defects, which are common disorders caused by trauma, pathology, or age-related degeneration, present a formidable challenge. This is because the absence of blood supply, nerves, and lymphangion increases osteoarthritis (OA) susceptibility. Various cell-based therapy strategies, including the implantation of autologous chondrocytes, local implantation of MSCs such as bone marrow-derived, adipose-derived, and synovium-derived MSCs, and specific growth factors such as TGF- and BMPs to stimulate cartilage generation, have been employed to address this issue.^305,306^ These are frequently combined with scaffolds such as HA or PLGA for improved MSCs delivery and differentiation into chondrocytes after implantation.^307,308^ Additionally, exosomes have been utilized alone or in conjunction with various scaffolds such as hydrogels.^309^

The use of a thermosensitive, injectable hydrogel as a vehicle for sEVs derived from primary chondrocytes in the early treatment of OA was investigated in a recent study.^310^ The hydrogel formed by cross-linking Pluronic F-127 and HA can gradually release sEVs, positively influencing chondrocyte growth, movement, and differentiation, and effectively inducing the transition from M1 to M2 macrophages. By promoting cartilage matrix formation, the injection of this sEVs-infused hydrogel into the joint significantly prevented cartilage damage. It also demonstrated a regenerative immune response characterized by a greater presence of CD163+ regenerative M2 macrophages in synovial and cartilage tissue than CD86+ M1 macrophages. In synovial fluid, this was accompanied by a decrease in pro-inflammatory cytokines (TNF-, IL-1, and IL-6) and an increase in anti-inflammatory cytokine (IL-10). The findings suggest that the local sustained release of sEVs derived from primary chondrocytes may alleviate OA by promoting the transformation of macrophages from M1 to M2, indicating significant therapeutic potential for OA.

**Figure 11:**
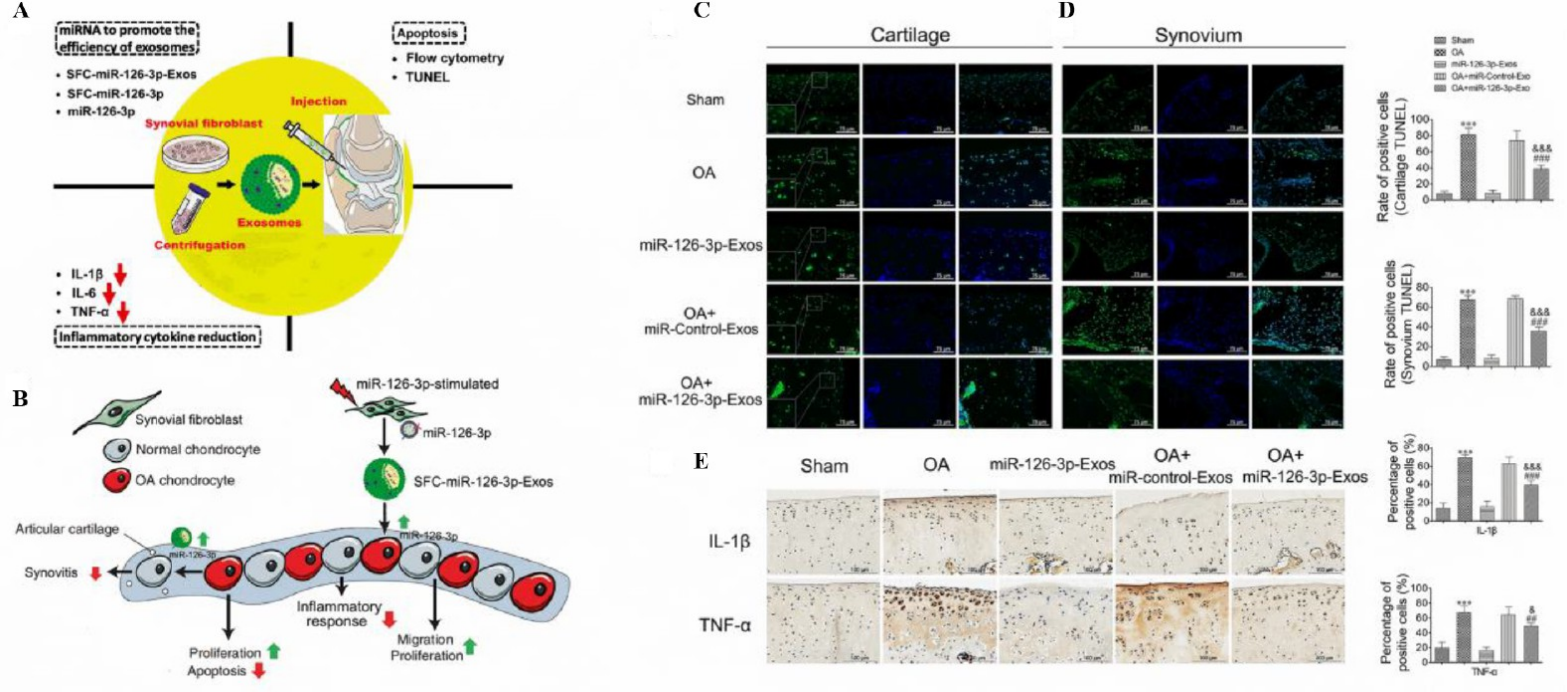
(A) The therapeutic effects of exosomes derived from SFC on OA are primarily reflected by the three parameters listed below. (1) reduction of inflammatory cytokines, (2) inhibition of apoptosis, and (3) modulation of miRNA-126–3p in exosome to improve therapeutic effectiveness. (B) Exosomes derived from SFC can mediate cell–cell communications and regulate diverse cell phenotypes, such as inflammatory response, cell proliferation, migration, apoptosis, etc. (C) A representative TUNEL-stained articular cartilage section was used to evaluate apoptosis. (D) A representative synovial tissue section stained with TUNEL was used to evaluate apoptosis. (E) IL-1 and TNF- immunohistochemical stains were performed on OA model rats. The ratios of immunoreactive positive cells were analyzed. Data were expressed as mean ± SEM (*n* = 5). Data were expressed as mean ± SEM (*n* = 5). ***P < 0.001 vs sham-operated group; ##*P* < 0.01 and ###*P* < 0.001 vs OA induction group; &*P* < 0.05, &&*P* < 0.01, and &&&*P* < 0.001 vs OA + miR-Control-Exos group. Reprinted with permission from ref (313). Copyright 2021 Springer Nature.

Exosomal miRNAs (such as miR-100–5p, miR-135b, miR-92a-3p, miR-140–5, miR-23b, miR-125b, miR-126–3p, miR-320) and lncRNAs like KLF3-AS1 and their interactions with diverse signaling pathways are primarily responsible for the therapeutic potential of MSCs-sEVs in the treatment of articular cartilage and OA.^311,312^ The function of miRNAs in sEVs derived from synovial fluid, focusing on their impact on chondrocyte inflammation, proliferation, and survival, as well as their potential effect on cartilage degeneration in an OA model in rats was examined. Nineteen miRNAs were identified whose expression levels differed between OA patients and healthy controls. Notably, was significantly reduced in the synovial fluid of OA patients. Using a miRNA-126–3p mimic, the researchers observed increased chondrocyte migration and proliferation, decreased apoptosis, and decreased expression of the pro-inflammatory cytokines IL-1, IL-6, and TNF. sEVs from synovial fluid cells (SFCs) carrying the microRNA-126–3p reduced chondrocyte apoptosis and inflammation. Experiments performed *in vivo* demonstrated that exosomal miRNA-126–3p from rat SFCs inhibits osteophyte formation and cartilage degeneration, and exerts antiapoptotic and anti-inflammatory effects on articular cartilage (Figure 11A–E). These results suggest that miRNA-126–3p delivered by sEVs from SFCs can reduce chondrocyte inflammation and cartilage degeneration, suggesting a potential therapeutic strategy for the treatment of OA.^313^ Therefore, MSCs-sEVs can be viewed as a ready-to-use and cell-free therapeutic strategy for complex tissue defects, such as joint injuries.

## 5 Future Perspectives

Future research will improve our understanding of how biomaterials interact with cellular responses, allowing these materials to adapt to environmental changes.^314^ This potential is exemplified by the biocompatible hydrogel Clustered Regularly Interspaced Short Palindromic Repeats (CRISP) system, which is based on endonuclease cas12a and contains single-stranded DNA in a polyethylene glycol hydrogel. It has the potential to improve the sustained release of drugs, nanoparticles, cells, and EVs lowering transplant rejection rates through the release of anti-inflammatory cytokines.^315^ Similarly, incorporating strontium into bioglass can modulate the immune response, thereby promoting bone regeneration via the sustained release of EVs from hydrogels.^316^

sEVs, particularly those obtained from MSCs, are recognized for their minimal immunogenicity, exceptional biocompatibility, and minimal biosafety risks. MSCs-sEVs have demonstrated the ability to promote regeneration and repair in various tissues, such as neurons and cardiomyocytes. Nevertheless, the precise mechanisms responsible for their therapeutic effects, primarily ascribed to their abundant protein, nucleic acid, and biological factor content, remain incompletely comprehended. The constitution of MSCs-sEVs obtained from different origins, such as human adipose MSCs and bone marrow MSCs, varies, offering both prospects and difficulties in their utilization.^317^ Because of their high water content and biocompatibility, hydrogels, particularly those containing exosomes, are increasingly being used in tissue engineering. The host–guest gelatin (HGM) supramolecular hydrogels and HA-pamidronate-grafted (HA-Pam) hydrogels have shown promise in enhancing cartilage formation and promoting the sustained release of small molecules and proteins.^318^

Despite these advances, there are still challenges in the clinical application of EVs-loaded hydrogels. Hydrogels’ poor mechanical properties may limit their use in bone and cartilage tissue repair. To improve these properties, natural and synthetic polymers, inorganic materials, and scaffolds are being modified. Furthermore, the low yield of EVs makes large-scale production difficult, necessitating advancements in EVs extraction technologies, bioreactors, 3D cell culture, and specialized reagents.^319^ Storage and stability of EV-loaded hydrogels are also issues. EVs typically require storage at −80 °C to maintain biological activity, and repeated freeze–thaw cycles can reduce their efficacy. Most current studies use ready-to-use EV-loaded hydrogels, with little emphasis on long-term storage and effectiveness maintenance. The production, storage, transportation, and economic considerations for these therapies are critical for clinical translation.^320^

Advancements in hydrogel manufacturing technology, such as the use of microfluidic-based methods for preparing hydrogel microspheres, fabricating microneedle patches, and employing hydrogel-based 3D bioprinting, will greatly enhance the potential for developing hydrogels loaded with EVs. These advancements are ready to bring about a new period of repairing human tissue and regenerative medicine, emphasizing the promising future of hydrogels loaded with EVs in these fields.^321^ While there are challenges in determining the kinetic release profile of EVs from scaffolds *in vivo*, the potential of bioactive exosome-laden scaffolds as alternatives to cell-based tissue engineering is significant. Continued research in this area is likely to yield solutions that improve their therapeutic potential.^322,323^

Furthermore, the incorporation of ML in the development and optimization of exosome-loaded hydrogels for tissue regeneration is critical. ML can improve the design and functionality of these hydrogels by allowing precise control over their physicochemical properties and release kinetics. This is especially important in overcoming obstacles like increasing the mechanical strength of hydrogels for bone and cartilage repair and ensuring the sustained, targeted delivery of therapeutic exosomes. Furthermore, the use of ML in exosome profiling is critical for comprehending their complex molecular composition, which has a direct impact on the efficacy of hydrogel-based delivery systems. In response to the referee’s concerns, our future research will concentrate on standardizing sensitive detection methods and investigating single exosome detection technologies, both of which are critical for the clinical translation of these advanced biomaterials. We hope to overcome current limitations and unlock the full potential of exosome-loaded hydrogels in regenerative medicine by leveraging the power of ML, paving the way for innovative treatments in tissue repair and beyond.

## 6 Conclusion

EVs have garnered significant attention in the field of regenerative medicine because of their ability to facilitate communication between cells and modulate cellular function. Their ability to carry bioactive molecules such as proteins, lipids, and nucleic acids makes them suitable as potential therapeutic agents. Nevertheless, the need for creative solutions has arisen due to obstacles such as swift elimination from the body and challenges in delivery. Hydrogels have become a favorable option for delivering EVs because they provide targeted and regulated release, thereby enhancing the effectiveness of therapy. The combination of EVs and hydrogels holds significant regenerative capacity for tissue. Furthermore, the integration of ML has completely transformed the analysis of EVs, enabling a more comprehensive comprehension of their intricate profiles. This Review offers a thorough examination of the present status of research, with a specific emphasis on the formation and composition of EVs, the characteristics and uses of hydrogels, and the potential of hydrogels containing EVs in the regeneration of tissues. As we further explore the intricacies of EVs biology and hydrogel technology, it becomes increasingly clear that their combined potential for regenerative medicine is highly promising.

## References

[ref1] Lu Y.; Mai Z.; Cui L.; Zhao X. Engineering exosomes and biomaterial-assisted exosomes as therapeutic carriers for bone regeneration. Stem Cell Res. Ther 2023, 14 (1), 55 10.1186/s13287-023-03275-x. 36978165 PMC10053084

[ref2] Tang Q.; et al. Exosomes-loaded thermosensitive hydrogels for corneal epithelium and stroma regeneration. Biomaterials 2022, 280, 121320 10.1016/j.biomaterials.2021.121320. 34923312

[ref3] Théry C. Minimal information for studies of extracellular vesicles 2018 (MISEV2018): a position statement of the International Society for Extracellular Vesicles and update of the MISEV2014 guidelines. J. Extracell Vesicles 2018, 7 (1), 1535750 10.1080/20013078.2018.1535750. 30637094 PMC6322352

[ref4] Dyball L. E.; Smales C. M. Exosomes: Biogenesis, targeting, characterization and their potential as ‘Plug & Play’ vaccine platforms. Biotechnol J. 2022, 17 (11), 2100646 10.1002/biot.202100646. 35899790

[ref5] Thakur A.; et al. Therapeutic Values of Exosomes in Cosmetics, Skin Care, Tissue Regeneration, and Dermatological Diseases. Cosmetics 2023, 10 (2), 65 10.3390/cosmetics10020065.

[ref6] Wang X.; Tian L.; Lu J.; Ng I. O.-L. Exosomes and cancer - Diagnostic and prognostic biomarkers and therapeutic vehicle. Oncogenesis 2022, 11 (1), 54 10.1038/s41389-022-00431-5. 36109501 PMC9477829

[ref7] Kalluri R.; LeBleu V. S. The biology, function, and biomedical applications of exosomes. Science (1979) 2020, 367 (6478), eaau6977 10.1126/science.aau6977. PMC7717626 32029601

[ref8] Tricarico C.; Clancy J.; D’Souza-Schorey C. Biology and biogenesis of shed microvesicles. Small GTPases 2017, 8 (4), 220–232. 10.1080/21541248.2016.1215283. 27494381 PMC5680703

[ref9] Doyle L.; Wang M. Overview of Extracellular Vesicles, Their Origin, Composition, Purpose, and Methods for Exosome Isolation and Analysis. Cells 2019, 8 (7), 727 10.3390/cells8070727. 31311206 PMC6678302

[ref10] Kakarla R.; Hur J.; Kim Y. J.; Kim J.; Chwae Y.-J. Apoptotic cell-derived exosomes: messages from dying cells. Exp Mol. Med. 2020, 52 (1), 1–6. 10.1038/s12276-019-0362-8. 31915368 PMC7000698

[ref11] Di Bella M. A. Overview and Update on Extracellular Vesicles: Considerations on Exosomes and Their Application in Modern Medicine. Biology (Basel) 2022, 11 (6), 804 10.3390/biology11060804. 35741325 PMC9220244

[ref12] Dinescu S. Exosomes as Part of the Human Adipose-Derived Stem Cells Secretome- Opening New Perspectives for Cell-Free Regenerative Applications. Adv. Exp. Med. Biol . 2020, 1312, 139–163. 10.1007/5584_2020_588. 32986128

[ref13] Tenchov R.; Sasso J. M.; Wang X.; Liaw W.-S.; Chen C.-A.; Zhou Q. A. Exosomes—Nature’s Lipid Nanoparticles, a Rising Star in Drug Delivery and Diagnostics. ACS Nano 2022, 16 (11), 17802–17846. 10.1021/acsnano.2c08774. 36354238 PMC9706680

[ref14] Xie Y.; Guan Q.; Guo J.; Chen Y.; Yin Y.; Han X. Hydrogels for Exosome Delivery in Biomedical Applications. Gels 2022, 8 (6), 328 10.3390/gels8060328. 35735672 PMC9223116

[ref15] Deng H.; Wang J.; An R. Hyaluronic acid-based hydrogels: As an exosome delivery system in bone regeneration. Front Pharmacol 2023, 14, 1 10.3389/fphar.2023.1131001. PMC10063825 37007032

[ref16] Guo L.; et al. Chitosan hydrogel, as a biological macromolecule-based drug delivery system for exosomes and microvesicles in regenerative medicine: a mini review. Cellulose 2022, 29 (3), 1315–1330. 10.1007/s10570-021-04330-7.

[ref17] Ma S.; et al. Improved intracellular delivery of exosomes by surface modification with fluorinated peptide dendrimers for promoting angiogenesis and migration of HUVECs. RSC Adv. 2023, 13 (17), 11269–11277. 10.1039/D3RA00300K. 37057265 PMC10087381

[ref18] Colombo M.; Raposo G.; Théry C. Biogenesis, Secretion, and Intercellular Interactions of Exosomes and Other Extracellular Vesicles. Annu. Rev. Cell Dev Biol. 2014, 30 (1), 255–289. 10.1146/annurev-cellbio-101512-122326. 25288114

[ref19] Juan T.; Fürthauer M. Biogenesis and function of ESCRT-dependent extracellular vesicles. Semin Cell Dev Biol. 2018, 74, 66–77. 10.1016/j.semcdb.2017.08.022. 28807885

[ref20] Baietti M. F.; et al. Syndecan-syntenin-ALIX regulates the biogenesis of exosomes. Nat. Cell Biol. 2012, 14 (7), 677–685. 10.1038/ncb2502. 22660413

[ref21] Juan T.; Fürthauer M. Biogenesis and function of ESCRT-dependent extracellular vesicles. Semin Cell Dev Biol. 2018, 74, 66–77. 10.1016/j.semcdb.2017.08.022. 28807885

[ref22] Kalluri R.; LeBleu V. S. The biology, function, and biomedical applications of exosomes. Science (1979) 2020, 367 (6478), eaau6977 10.1126/science.aau6977. PMC7717626 32029601

[ref23] Babst M. MVB vesicle formation: ESCRT-dependent, ESCRT-independent and everything in between. Curr. Opin Cell Biol. 2011, 23 (4), 452–457. 10.1016/j.ceb.2011.04.008. 21570275 PMC3148405

[ref24] Perez-Hernandez D.; et al. The Intracellular Interactome of Tetraspanin-enriched Microdomains Reveals Their Function as Sorting Machineries toward Exosomes. J. Biol. Chem. 2013, 288 (17), 11649–11661. 10.1074/jbc.M112.445304. 23463506 PMC3636856

[ref25] Andreu Z.; Yanez-Mo M. Tetraspanins in Extracellular Vesicle Formation and Function. Front Immunol 2014, 5, 1 10.3389/fimmu.2014.00442. 25278937 PMC4165315

[ref26] Nazarenko I.; et al. Cell Surface Tetraspanin Tspan8 Contributes to Molecular Pathways of Exosome-Induced Endothelial Cell Activation. Cancer Res. 2010, 70 (4), 1668–1678. 10.1158/0008-5472.CAN-09-2470. 20124479

[ref27] Chairoungdua A.; Smith D. L.; Pochard P.; Hull M.; Caplan M. J. Exosome release of β-catenin: a novel mechanism that antagonizes Wnt signaling. J. Cell Biol. 2010, 190 (6), 1079–1091. 10.1083/jcb.201002049. 20837771 PMC3101591

[ref28] Bissig C.; Gruenberg J. ALIX and the multivesicular endosome: ALIX in Wonderland. Trends Cell Biol. 2014, 24 (1), 19–25. 10.1016/j.tcb.2013.10.009. 24287454

[ref29] Géminard C.; de Gassart A.; Blanc L.; Vidal M. Degradation of AP2 During Reticulocyte Maturation Enhances Binding of Hsc70 and Alix to a Common Site on TfR for Sorting into Exosomes. Traffic 2004, 5 (3), 181–193. 10.1111/j.1600-0854.2004.0167.x. 15086793

[ref30] Raposo G.; Stoorvogel W. Extracellular vesicles: Exosomes, microvesicles, and friends. J. Cell Biol. 2013, 200 (4), 373–383. 10.1083/jcb.201211138. 23420871 PMC3575529

[ref31] Curtaz C. J.; et al. Analysis of microRNAs in Exosomes of Breast Cancer Patients in Search of Molecular Prognostic Factors in Brain Metastases. Int. J. Mol. Sci. 2022, 23 (7), 3683 10.3390/ijms23073683. 35409043 PMC8999078

[ref32] Ju C.; Liu D. Exosomal microRNAs from Mesenchymal Stem Cells: Novel Therapeutic Effect in Wound Healing. Tissue Eng. Regen Med. 2023, 20 (5), 647–660. 10.1007/s13770-023-00542-z. 37131016 PMC10352215

[ref33] Villarroya-Beltri C.; et al. Sumoylated hnRNPA2B1 controls the sorting of miRNAs into exosomes through binding to specific motifs. Nat. Commun. 2013, 4 (1), 2980 10.1038/ncomms3980. 24356509 PMC3905700

[ref34] Katakowski M.; et al. Exosomes from marrow stromal cells expressing miR-146b inhibit glioma growth. Cancer Lett. 2013, 335 (1), 201–204. 10.1016/j.canlet.2013.02.019. 23419525 PMC3665755

[ref35] Kosaka N.; Iguchi H.; Yoshioka Y.; Takeshita F.; Matsuki Y.; Ochiya T. Secretory Mechanisms and Intercellular Transfer of MicroRNAs in Living Cells. J. Biol. Chem. 2010, 285 (23), 17442–17452. 10.1074/jbc.M110.107821. 20353945 PMC2878508

[ref36] Gibbings D. J.; Ciaudo C.; Erhardt M.; Voinnet O. Multivesicular bodies associate with components of miRNA effector complexes and modulate miRNA activity. Nat. Cell Biol. 2009, 11 (9), 1143–1149. 10.1038/ncb1929. 19684575

[ref37] O’Brien K.; Breyne K.; Ughetto S.; Laurent L. C.; Breakefield X. O. RNA delivery by extracellular vesicles in mammalian cells and its applications. Nat. Rev. Mol. Cell Biol. 2020, 21 (10), 585–606. 10.1038/s41580-020-0251-y. 32457507 PMC7249041

[ref38] Ashley J.; Cordy B.; Lucia D.; Fradkin L. G.; Budnik V.; Thomson T. Retrovirus-like Gag Protein Arc1 Binds RNA and Traffics across Synaptic Boutons. Cell 2018, 172 (1–2), 262–274. 10.1016/j.cell.2017.12.022. 29328915 PMC5793882

[ref39] Carnino J. M.; Ni K.; Jin Y. Post-translational Modification Regulates Formation and Cargo-Loading of Extracellular Vesicles. Front Immunol 2020, 11, 1 10.3389/fimmu.2020.00948. 32528471 PMC7257894

[ref40] Wei H.; et al. Regulation of exosome production and cargo sorting. Int. J. Biol. Sci. 2021, 17 (1), 163–177. 10.7150/ijbs.53671. 33390841 PMC7757038

[ref41] Villarroya-Beltri C.; et al. ISGylation controls exosome secretion by promoting lysosomal degradation of MVB proteins. Nat. Commun. 2016, 7 (1), 13588 10.1038/ncomms13588. 27882925 PMC5123068

[ref42] Kunadt M.; et al. Extracellular vesicle sorting of α-Synuclein is regulated by sumoylation. Acta Neuropathol 2015, 129 (5), 695–713. 10.1007/s00401-015-1408-1. 25778619 PMC4405286

[ref43] van Niel G.; D’Angelo G.; Raposo G. Shedding light on the cell biology of extracellular vesicles. Nat. Rev. Mol. Cell Biol. 2018, 19 (4), 213–228. 10.1038/nrm.2017.125. 29339798

[ref44] Drobiova H.; Sindhu S.; Ahmad R.; Haddad D.; Al-Mulla F.; Al Madhoun A. Wharton’s jelly mesenchymal stem cells: a concise review of their secretome and prospective clinical applications. Front Cell Dev Biol. 2023, 11, 1 10.3389/fcell.2023.1211217. PMC10333601 37440921

[ref45] Harding C. V.; Heuser J. E.; Stahl P. D. Exosomes: Looking back three decades and into the future. J. Cell Biol. 2013, 200 (4), 367–371. 10.1083/jcb.201212113. 23420870 PMC3575527

[ref46] Zhang Y.; Liu Y.; Liu H.; Tang W. H. Exosomes: biogenesis, biologic function and clinical potential. Cell Biosci 2019, 9 (1), 19 10.1186/s13578-019-0282-2. 30815248 PMC6377728

[ref47] Ma Y.; Brocchini S.; Williams G. R. Extracellular vesicle-embedded materials. J. Controlled Release 2023, 361, 280–296. 10.1016/j.jconrel.2023.07.059. 37536545

[ref48] Poon I. K. H. Moving beyond size and phosphatidylserine exposure: evidence for a diversity of apoptotic cell-derived extracellular vesicles *in vitro*. J. Extracell Vesicles 2019, 8 (1), 1608786 10.1080/20013078.2019.1608786. 31069027 PMC6493268

[ref49] Caruso S.; Poon I. K. H. Apoptotic Cell-Derived Extracellular Vesicles: More Than Just Debris. Front Immunol 2018, 9, 1 10.3389/fimmu.2018.01486. 30002658 PMC6031707

[ref50] Hartmann R. C.; Conley C. L.; Poole E. L. STUDIES ON THE INITIATION OF BLOOD COAGULATION. III. THE CLOTTING PROPERTIES OF CANINE PLATELET FREE PLASMA. J. Clin. Invest. 1952, 31, 685 10.1172/JCI102650. 14938443 PMC436461

[ref51] Wolf P. The Nature and Significance of Platelet Products in Human Plasma. Br. J. Hamaetol. 1967, 13 (3), 269–288. 10.1111/j.1365-2141.1967.tb08741.x. 6025241

[ref52] Dannies P. S.; Rudnick M. S.; Fishkes H.; Rudnick G. Spiperone: evidence for uptake into secretory granules. Proc. Natl. Acad. Sci. U. S. A. 1984, 81 (6), 1867–1870. 10.1073/pnas.81.6.1867. 6584920 PMC345023

[ref53] Pan B. T.; Teng K.; Wu C.; Adam M.; Johnstone R. M. Electron microscopic evidence for externalization of the transferrin receptor in vesicular form in sheep reticulocytes. J. Cell Biol. 1985, 101 (3), 942–948. 10.1083/jcb.101.3.942. 2993317 PMC2113705

[ref54] György B.; et al. Membrane vesicles, current state-of-the-art: emerging role of extracellular vesicles. Cell. Mol. Life Sci. 2011, 68 (16), 2667–2688. 10.1007/s00018-011-0689-3. 21560073 PMC3142546

[ref55] Azmi A. S.; Bao B.; Sarkar F. H. Exosomes in cancer development, metastasis, and drug resistance: a comprehensive review. Cancer and Metastasis Reviews 2013, 32 (3–4), 623–642. 10.1007/s10555-013-9441-9. 23709120 PMC3843988

[ref56] van Niel G.; Porto-Carreiro I.; Simoes S.; Raposo G. Exosomes: A Common Pathway for a Specialized Function. Journal of Biochemistry 2006, 140 (1), 13–21. 10.1093/jb/mvj128. 16877764

[ref57] Valadi H.; Ekström K.; Bossios A.; Sjöstrand M.; Lee J. J.; Lötvall J. O. Exosome-mediated transfer of mRNAs and microRNAs is a novel mechanism of genetic exchange between cells. Nat. Cell Biol. 2007, 9 (6), 654–659. 10.1038/ncb1596. 17486113

[ref58] D’Asti E.; Garnier D.; Lee T. H.; Montermini L.; Meehan B.; Rak J. Oncogenic extracellular vesicles in brain tumor progression. Front Physiol 2012, 3, 1 10.3389/fphys.2012.00294. 22934045 PMC3429065

[ref59] Mathivanan S.; Ji H.; Simpson R. J. Exosomes: Extracellular organelles important in intercellular communication. J. Proteomics 2010, 73 (10), 1907–1920. 10.1016/j.jprot.2010.06.006. 20601276

[ref60] Poliakov A.; Spilman M.; Dokland T.; Amling C. L.; Mobley J. A. Structural heterogeneity and protein composition of exosome-like vesicles (prostasomes) in human semen. Prostate 2009, 69 (2), 159–167. 10.1002/pros.20860. 18819103

[ref61] Minciacchi V. R.; Freeman M. R.; Di Vizio D. Extracellular Vesicles in Cancer: Exosomes, Microvesicles and the Emerging Role of Large Oncosomes. Semin Cell Dev Biol. 2015, 40, 41–51. 10.1016/j.semcdb.2015.02.010. 25721812 PMC4747631

[ref62] Chu Z.; Witte D. P.; Qi X. Saposin C-LBPA interaction in late-endosomes/lysosomes. Exp. Cell Res. 2005, 303 (2), 300–307. 10.1016/j.yexcr.2004.09.029. 15652344

[ref63] Vidal M.; Sainte-Marie J.; Philippot J. R.; Bienvenue A. Asymmetric distribution of phospholipids in the membrane of vesicles released during in vitro maturation of guinea pig reticulocytes: Evidence precluding a role for ?aminophospholipid translocase?. J. Cell Physiol 1989, 140 (3), 455–462. 10.1002/jcp.1041400308. 2777884

[ref64] Bissig C.; et al. Viral Infection Controlled by a Calcium-Dependent Lipid-Binding Module in ALIX. Dev Cell 2013, 25 (4), 364–373. 10.1016/j.devcel.2013.04.003. 23664863 PMC4129370

[ref65] LAULAGNIER K.; MOTTA C.; HAMDI S.; ROY S.; FAUVELLE F.; PAGEAUX J.-F.; KOBAYASHI T.; SALLES J.-P.; PERRET B.; BONNEROT C.; RECORD M.; et al. Mast cell- and dendritic cell-derived exosomes display a specific lipid composition and an unusual membrane organization. Biochem. J. 2004, 380 (1), 161–171. 10.1042/bj20031594. 14965343 PMC1224152

[ref66] Huotari J.; Helenius A. Endosome maturation. EMBO J. 2011, 30 (17), 3481–3500. 10.1038/emboj.2011.286. 21878991 PMC3181477

[ref67] Simbari F. Plasmalogen enrichment in exosomes secreted by a nematode parasite versus those derived from its mouse host: implications for exosome stability and biology. J. Extracell Vesicles 2016, 5 (1), 1 10.3402/jev.v5.30741. PMC4937767 27389011

[ref68] LAULAGNIER K.; MOTTA C.; HAMDI S.; ROY S.; FAUVELLE F.; PAGEAUX J.-F.; KOBAYASHI T.; SALLES J.-P.; PERRET B.; BONNEROT C.; RECORD M.; et al. Mast cell- and dendritic cell-derived exosomes display a specific lipid composition and an unusual membrane organization. Biochem. J. 2004, 380 (1), 161–171. 10.1042/bj20031594. 14965343 PMC1224152

[ref69] Jalabert A.; et al. Profiling of ob/ob mice skeletal muscle exosome-like vesicles demonstrates combined action of miRNAs, proteins and lipids to modulate lipid homeostasis in recipient cells. Sci. Rep 2021, 11 (1), 21626 10.1038/s41598-021-00983-3. 34732797 PMC8566600

[ref70] Liu Y. Perivascular Adipose-Derived Exosomes Reduce Foam Cell Formation by Regulating Expression of Cholesterol Transporters. Front Cardiovasc Med. 2021, 8, 1 10.3389/fcvm.2021.697510. PMC8416751 34490366

[ref71] Doyle L.; Wang M. Overview of Extracellular Vesicles, Their Origin, Composition, Purpose, and Methods for Exosome Isolation and Analysis. Cells 2019, 8 (7), 727 10.3390/cells8070727. 31311206 PMC6678302

[ref72] Akers J. C.; Gonda D.; Kim R.; Carter B. S.; Chen C. C. Biogenesis of extracellular vesicles (EV): exosomes, microvesicles, retrovirus-like vesicles, and apoptotic bodies. J. Neurooncol 2013, 113 (1), 1–11. 10.1007/s11060-013-1084-8. 23456661 PMC5533094

[ref73] Muralidharan-Chari V.; et al. ARF6-Regulated Shedding of Tumor Cell-Derived Plasma Membrane Microvesicles. Curr. Biol. 2009, 19 (22), 1875–1885. 10.1016/j.cub.2009.09.059. 19896381 PMC3150487

[ref74] Zou X.; et al. Advances in biological functions and applications of apoptotic vesicles. Cell Communication and Signaling 2023, 21 (1), 260 10.1186/s12964-023-01251-9. 37749626 PMC10519056

[ref75] Davidson S. M.; et al. Methods for the identification and characterization of extracellular vesicles in cardiovascular studies: from exosomes to microvesicles. Cardiovasc. Res. 2023, 119 (1), 45–63. 10.1093/cvr/cvac031. 35325061 PMC10233250

[ref76] Paolini L.; et al. Residual matrix from different separation techniques impacts exosome biological activity. Sci. Rep 2016, 6 (1), 23550 10.1038/srep23550. 27009329 PMC4806376

[ref77] Böing A. N.; van der Pol E.; Grootemaat A. E.; Coumans F. A. W.; Sturk A.; Nieuwland R. Single-step isolation of extracellular vesicles by size-exclusion chromatography. J. Extracell Vesicles 2014, 3 (1), 1 10.3402/jev.v3.23430. PMC4159761 25279113

[ref78] Kang Y.-T.; Kim Y. J.; Bu J.; Cho Y.-H.; Han S.-W.; Moon B.-I. High-purity capture and release of circulating exosomes using an exosome-specific dual-patterned immunofiltration (ExoDIF) device. Nanoscale 2017, 9 (36), 13495–13505. 10.1039/C7NR04557C. 28862274

[ref79] Théry C.; Amigorena S.; Raposo G.; Clayton A. Isolation and Characterization of Exosomes from Cell Culture Supernatants and Biological Fluids. Curr. Protoc Cell Biol. 2006, 30 (1), 1 10.1002/0471143030.cb0322s30. 18228490

[ref80] Arraud N.; et al. Extracellular vesicles from blood plasma: determination of their morphology, size, phenotype and concentration. Journal of Thrombosis and Haemostasis 2014, 12 (5), 614–627. 10.1111/jth.12554. 24618123

[ref81] Ridolfi A.; et al. AFM-Based High-Throughput Nanomechanical Screening of Single Extracellular Vesicles. Anal. Chem. 2020, 92 (15), 10274–10282. 10.1021/acs.analchem.9b05716. 32631050

[ref82] Nolan J. P. Flow Cytometry of Extracellular Vesicles: Potential, Pitfalls, and Prospects,. Curr. Protoc Cytom 2015, 73 (1), 13.14.1 10.1002/0471142956.cy1314s73. 26132176

[ref83] Libregts S. F. W. M.; Arkesteijn G. J. A.; Németh A.; Nolte-’t Hoen E. N. M.; Wauben M. H. M. Flow cytometric analysis of extracellular vesicle subsets in plasma: impact of swarm by particles of non-interest,. Journal of Thrombosis and Haemostasis 2018, 16 (7), 1423–1436. 10.1111/jth.14154. 29781099

[ref84] Welsh J. A. MIFlowCyt-EV: a framework for standardized reporting of extracellular vesicle flow cytometry experiments. J. Extracell Vesicles 2020, 9 (1), 1 10.1080/20013078.2020.1713526. PMC7034442 32128070

[ref85] Koganti S.; Eleftheriou D.; Gurung R.; Hong Y.; Brogan P.; Rakhit R. D. Persistent circulating platelet and endothelial derived microparticle signature may explain on-going pro-thrombogenicity after acute coronary syndrome. Thromb Res. 2021, 206, 60–65. 10.1016/j.thromres.2021.07.018. 34418680

[ref86] Kränkel N.; et al. Extracellular vesicle species differentially affect endothelial cell functions and differentially respond to exercise training in patients with chronic coronary syndromes. Eur. J. Prev Cardiol 2021, 28 (13), 1467–1474. 10.1177/2047487320919894. 34695219

[ref87] Amabile N.; et al. Association of circulating endothelial microparticles with cardiometabolic risk factors in the Framingham Heart Study. Eur. Heart J. 2014, 35 (42), 2972–2979. 10.1093/eurheartj/ehu153. 24742886 PMC4223610

[ref88] Anselmo A.; et al. Myocardial hypoxic stress mediates functional cardiac extracellular vesicle release. Eur. Heart J. 2021, 42 (28), 2780–2792. 10.1093/eurheartj/ehab247. 34104945

[ref89] Takov K.; et al. Small extracellular vesicles secreted from human amniotic fluid mesenchymal stromal cells possess cardioprotective and promigratory potential. Basic Res. Cardiol 2020, 115 (3), 26 10.1007/s00395-020-0785-3. 32146560 PMC7060967

[ref90] Boyden S. THE, CHEMOTACTIC EFFECT OF MIXTURES OF ANTIBODY AND ANTIGEN ON POLYMORPHONUCLEAR LEUCOCYTES. J. Exp Med. 1962, 115 (3), 453–466. 10.1084/jem.115.3.453. 13872176 PMC2137509

[ref91] Liang C.-C.; Park A. Y.; Guan J.-L. In vitro scratch assay: a convenient and inexpensive method for analysis of cell migration in vitro. Nat. Protoc 2007, 2 (2), 329–333. 10.1038/nprot.2007.30. 17406593

[ref92] Baker M.; et al. Use of the mouse aortic ring assay to study angiogenesis. Nat. Protoc 2012, 7 (1), 89–104. 10.1038/nprot.2011.435. 22193302

[ref93] Todorova D.; Simoncini S.; Lacroix R.; Sabatier F.; Dignat-George F. Extracellular Vesicles in Angiogenesis. Circ. Res. 2017, 120 (10), 1658–1673. 10.1161/CIRCRESAHA.117.309681. 28495996 PMC5426696

[ref94] Ribeiro-Rodrigues T. M.; et al. Exosomes secreted by cardiomyocytes subjected to ischaemia promote cardiac angiogenesis. Cardiovasc. Res. 2017, 113 (11), 1338–1350. 10.1093/cvr/cvx118. 28859292

[ref95] Gimona M.; et al. Critical considerations for the development of potency tests for therapeutic applications of mesenchymal stromal cell-derived small extracellular vesicles. Cytotherapy 2021, 23 (5), 373–380. 10.1016/j.jcyt.2021.01.001. 33934807

[ref96] Ferguson S. W.; Nguyen J. Exosomes as therapeutics: The implications of molecular composition and exosomal heterogeneity. J. Controlled Release 2016, 228, 179–190. 10.1016/j.jconrel.2016.02.037. 26941033

[ref97] Xiong H. Recent Progress in Detection and Profiling of Cancer Cell-Derived Exosomes. Small 2021, 17 (35), 2007971 10.1002/smll.202007971. 34075696

[ref98] Willis G. R.; Kourembanas S.; Mitsialis S. A. Toward Exosome-Based Therapeutics: Isolation, Heterogeneity, and Fit-for-Purpose Potency. Front Cardiovasc Med. 2017, 4, 1 10.3389/fcvm.2017.00063. 29062835 PMC5640880

[ref99] Barrera-Ramirez J.; et al. Micro-RNA Profiling of Exosomes from Marrow-Derived Mesenchymal Stromal Cells in Patients with Acute Myeloid Leukemia: Implications in Leukemogenesis. Stem Cell Rev. Rep 2017, 13 (6), 817–825. 10.1007/s12015-017-9762-0. 28918518 PMC5730624

[ref100] Kim C. W.; Lee H. M.; Lee T. H.; Kang C.; Kleinman H. K.; Gho Y. S. Extracellular membrane vesicles from tumor cells promote angiogenesis via sphingomyelin. Cancer Res. 2002, 62 (21), 6312–7. 12414662

[ref101] Deo R. C. Machine Learning in Medicine. Circulation 2015, 132 (20), 1920–1930. 10.1161/CIRCULATIONAHA.115.001593. 26572668 PMC5831252

[ref102] Gulshan V.; et al. Development and Validation of a Deep Learning Algorithm for Detection of Diabetic Retinopathy in Retinal Fundus Photographs. JAMA 2016, 316 (22), 2402 10.1001/jama.2016.17216. 27898976

[ref103] Ko J.; et al. Combining Machine Learning and Nanofluidic Technology To Diagnose Pancreatic Cancer Using Exosomes. ACS Nano 2017, 11 (11), 11182–11193. 10.1021/acsnano.7b05503. 29019651

[ref104] Kennel P. J.; et al. Serum exosomal protein profiling for the non-invasive detection of cardiac allograft rejection. Journal of Heart and Lung Transplantation 2018, 37 (3), 409–417. 10.1016/j.healun.2017.07.012. 28789823

[ref105] Wu N.; Zhang X.-Y.; Xia J.; Li X.; Yang T.; Wang J.-H. Ratiometric 3D DNA Machine Combined with Machine Learning Algorithm for Ultrasensitive and High-Precision Screening of Early Urinary Diseases. ACS Nano 2021, 15 (12), 19522–19534. 10.1021/acsnano.1c06429. 34813275

[ref106] Park J.; et al. Exosome Classification by Pattern Analysis of Surface-Enhanced Raman Spectroscopy Data for Lung Cancer Diagnosis. Anal. Chem. 2017, 89 (12), 6695–6701. 10.1021/acs.analchem.7b00911. 28541032

[ref107] Zlotogorski-Hurvitz A.; Dekel B. Z.; Malonek D.; Yahalom R.; Vered M. FTIR-based spectrum of salivary exosomes coupled with computational-aided discriminating analysis in the diagnosis of oral cancer. J. Cancer Res. Clin Oncol 2019, 145 (3), 685–694. 10.1007/s00432-018-02827-6. 30603907 PMC11810221

[ref108] Ho T.-C.; et al. Hydrogels: Properties and Applications in Biomedicine. Molecules 2022, 27 (9), 2902 10.3390/molecules27092902. 35566251 PMC9104731

[ref109] Dror M.; Elsabee M. Z.; Berry G. C. Interpenetrating Polymer Networks for Biological Applications. Biomater Med. Devices Artif Organs 1979, 7 (1), 31–39. 10.3109/10731197909119370. 454781

[ref110] WICHTERLE O.; LÍM D. Hydrophilic Gels for Biological Use. Nature 1960, 185 (4706), 117–118. 10.1038/185117a0.

[ref111] Wanasekara N.; Chen M.; Chalivendra V.; Bhowmick S. Investigation of the Young’s Modulus of Fibers in an Electrospun PCL Scaffold Using AFM and its Correlation to cell Attachment. MEMS and Nanotechnology 2011, 2, 157–162. 10.1007/978-1-4419-8825-6_22.

[ref112] Peppas N. A.; Hilt J. Z.; Khademhosseini A.; Langer R. Hydrogels in Biology and Medicine: From Molecular Principles to Bionanotechnology. Adv. Mater. 2006, 18 (11), 1345–1360. 10.1002/adma.200501612.

[ref113] Bellamkonda R.; Ranieri J. P.; Bouche N.; Aebischer P. Hydrogel-based three-dimensional matrix for neural cells. J. Biomed Mater. Res. 1995, 29 (5), 663–671. 10.1002/jbm.820290514. 7622552

[ref114] Madduma-Bandarage U. S. K.; Madihally S. V. Synthetic hydrogels: Synthesis, novel trends, and applications. J. Appl. Polym. Sci. 2021, 138 (19), 50376 10.1002/app.50376.

[ref115] Choi H.; Choi Y.; Yim H. Y.; Mirzaaghasi A.; Yoo J.-K.; Choi C. Biodistribution of Exosomes and Engineering Strategies for Targeted Delivery of Therapeutic Exosomes. Tissue Eng. Regen Med. 2021, 18 (4), 499–511. 10.1007/s13770-021-00361-0. 34260047 PMC8325750

[ref116] Khayambashi P.; Iyer J.; Pillai S.; Upadhyay A.; Zhang Y.; Tran S. Hydrogel Encapsulation of Mesenchymal Stem Cells and Their Derived Exosomes for Tissue Engineering. Int. J. Mol. Sci. 2021, 22 (2), 684 10.3390/ijms22020684. 33445616 PMC7827932

[ref117] Yerneni S. S.; et al. Controlled Release of Exosomes Using Atom Transfer Radical Polymerization-Based Hydrogels. Biomacromolecules 2022, 23 (4), 1713–1722. 10.1021/acs.biomac.1c01636. 35302760

[ref118] Shi Q. GMSC-Derived Exosomes Combined with a Chitosan/Silk Hydrogel Sponge Accelerates Wound Healing in a Diabetic Rat Skin Defect Model. Front Physiol 2017, 8, 1 10.3389/fphys.2017.00904. 29163228 PMC5681946

[ref119] Gbenebor O. P.; Adeosun S. O.; Lawal G. I.; Jun S.; Olaleye S. A. Acetylation, crystalline and morphological properties of structural polysaccharide from shrimp exoskeleton. Engineering Science and Technology, an International Journal 2017, 20 (3), 1155–1165. 10.1016/j.jestch.2017.05.002.

[ref120] Peppas N. A.; Merrill E. W. Development of semicrystalline poly(vinyl alcohol) hydrogels for biomedical applications. J. Biomed Mater. Res. 1977, 11 (3), 423–434. 10.1002/jbm.820110309. 853047

[ref121] Zhang Y.; Liu Y.; Liu J.; Guo P.; Heng L. Super water absorbency OMMT/PAA hydrogel materials with excellent mechanical properties. RSC Adv. 2017, 7 (24), 14504–14510. 10.1039/C7RA00372B.

[ref122] Ezati M.; Safavipour H.; Houshmand B.; Faghihi S. Development of a PCL/gelatin/chitosan/β-TCP electrospun composite for guided bone regeneration. Prog. Biomater 2018, 7 (3), 225–237. 10.1007/s40204-018-0098-x. 30242739 PMC6173671

[ref123] Ranjha N. M.; Mudassir J.; Akhtar N. Methyl methacrylate-co-itaconic acid (MMA-co-IA) hydrogels for controlled drug delivery. J. Solgel Sci. Technol. 2008, 47 (1), 23–30. 10.1007/s10971-008-1750-z.

[ref124] Hahn S. K.; Park J. K.; Tomimatsu T.; Shimoboji T. Synthesis and degradation test of hyaluronic acid hydrogels. Int. J. Biol. Macromol. 2007, 40 (4), 374–380. 10.1016/j.ijbiomac.2006.09.019. 17101173

[ref125] Yean L.; Bunel C.; Vairon J.-P. Reversible immobilization of drugs on a hydrogel matrix, 2†. Diffusion of free chloramphenicol from poly(2-hydroxyethyl methacrylate) hydrogels. Makromol. Chem. 1990, 191 (5), 1119–1129. 10.1002/macp.1990.021910514.

[ref126] Song S. Z.; Cardinalx J. R.; Kim S. H.; Kim S. W. Progestin Permeation Through Polymer Membranes V: Progesterone Release from Monolithic Hydrogel Devices. J. Pharm. Sci. 1981, 70 (2), 216–219. 10.1002/jps.2600700226. 7205230

[ref127] Korsmeyer R. W.; Peppas N. A. Effect of the morphology of hydrophilic polymeric matrices on the diffusion and release of water soluble drugs. J. Membr. Sci. 1981, 9 (3), 211–227. 10.1016/S0376-7388(00)80265-3.

[ref128] Soon-Shiong P.; et al. Insulin independence in a type 1 diabetic patient after encapsulated islet transplantation. Lancet 1994, 343 (8903), 950–951. 10.1016/S0140-6736(94)90067-1. 7909011

[ref129] Rowley J. A.; Madlambayan G.; Mooney D. J. Alginate hydrogels as synthetic extracellular matrix materials. Biomaterials 1999, 20 (1), 45–53. 10.1016/S0142-9612(98)00107-0. 9916770

[ref130] Liu T. Effect of Freezing Process on the Microstructure of Gelatin Methacryloyl Hydrogels. Front Bioeng Biotechnol 2021, 9, 1 10.3389/fbioe.2021.810155. PMC8717941 34976995

[ref131] Lin C.-Y.; Battistoni C. M.; Liu J. C. Redox-Responsive Hydrogels with Decoupled Initial Stiffness and Degradation. Biomacromolecules 2021, 22 (12), 5270–5280. 10.1021/acs.biomac.1c01180. 34793135

[ref132] Miao L.; et al. Alkynyl-functionalization of hydroxypropyl cellulose and thermoresponsive hydrogel thereof prepared with P(NIPAAm- co -HEMAPCL). Carbohydr. Polym. 2016, 137, 433–440. 10.1016/j.carbpol.2015.11.001. 26686148

[ref133] Kouchak M. In Situ Gelling Systems for Drug Delivery. Jundishapur J. Nat. Pharm. Prod 2014, 9 (3), e20126 10.17795/jjnpp-20126. 25237648 PMC4165193

[ref134] Wu W.-C.; et al. Theoretical and Experimental Studies on the Surface Structures of Conjugated Rod-Coil Block Copolymer Brushes. Langmuir 2007, 23 (5), 2805–2814. 10.1021/la0631769. 17249707

[ref135] Yan K.; et al. A multifunctional metal-biopolymer coordinated double network hydrogel combined with multi-stimulus responsiveness, self-healing, shape memory and antibacterial properties. Biomater Sci. 2020, 8 (11), 3193–3201. 10.1039/D0BM00425A. 32373851

[ref136] Haraguchi K.; Takehisa T. Nanocomposite Hydrogels: A Unique Organic-Inorganic Network Structure with Extraordinary Mechanical, Optical, and Swelling/De-swelling Properties. Adv. Mater. 2002, 14 (16), 1120 10.1002/1521-4095(20020816)14:16<1120::AID-ADMA1120>3.0.CO;2-9.

[ref137] Lu Z.-R.; Kopečková P.; Kopeček J. Antigen Responsive Hydrogels Based on Polymerizable Antibody Fab′ Fragment. Macromol. Biosci 2003, 3 (6), 296–300. 10.1002/mabi.200390039.

[ref138] Wen X.; Zhang Y.; Chen D.; Zhao Q. Reversible Shape-Shifting of an Ionic Strength Responsive Hydrogel Enabled by Programmable Network Anisotropy. ACS Appl. Mater. Interfaces 2022, 14 (35), 40344–40350. 10.1021/acsami.2c11693. 36017981

[ref139] Peppas N. A.; Stauffer S. R. Reinforced uncrosslinked poly (vinyl alcohol) gels produced by cyclic freezing-thawing processes: a short review. J. Controlled Release 1991, 16 (3), 305–310. 10.1016/0168-3659(91)90007-Z.

[ref140] Hu Y.; Han W.; Huang G.; Zhou W.; Yang Z.; Wang C. Highly Stretchable, Mechanically Strong, Tough, and Self-Recoverable Nanocomposite Hydrogels by Introducing Strong Ionic Coordination Interactions. Macromol. Chem. Phys. 2016, 217 (24), 2717–2725. 10.1002/macp.201600398.

[ref141] Wang X.; Wei C.; Cao B.; Jiang L.; Hou Y.; Chang J. Fabrication of Multiple-Layered Hydrogel Scaffolds with Elaborate Structure and Good Mechanical Properties via 3D Printing and Ionic Reinforcement. ACS Appl. Mater. Interfaces 2018, 10 (21), 18338–18350. 10.1021/acsami.8b04116. 29718655

[ref142] Wei Q.; Duan J.; Ma G.; Zhang W.; Wang Q.; Hu Z. Enzymatic crosslinking to fabricate antioxidant peptide-based supramolecular hydrogel for improving cutaneous wound healing. J. Mater. Chem. B 2019, 7 (13), 2220–2225. 10.1039/C8TB03147A. 32073581

[ref143] Iwanaga S.; et al. Design and Fabrication of Mature Engineered Pre-Cardiac Tissue Utilizing 3D Bioprinting Technology and Enzymatically Crosslinking Hydrogel. Materials 2022, 15 (22), 7928 10.3390/ma15227928. 36431414 PMC9693247

[ref144] Shen S.; Shen J.; Shen H.; Wu C.; Chen P.; Wang Q. Dual-Enzyme Crosslinking and Post-polymerization for Printing of Polysaccharide-Polymer Hydrogel. Front Chem. 2020, 8, 1 10.3389/fchem.2020.00036. 32117869 PMC7025582

[ref145] Bashir S.; et al. Fundamental Concepts of Hydrogels: Synthesis, Properties, and Their Applications. Polymers (Basel) 2020, 12 (11), 2702 10.3390/polym12112702. 33207715 PMC7697203

[ref146] Zhang Y.; Huang Y. Rational Design of Smart Hydrogels for Biomedical Applications. Front Chem. 2021, 8, 1 10.3389/fchem.2020.615665. PMC7889811 33614595

[ref147] Benwood C.; et al. Natural Biomaterials and Their Use as Bioinks for Printing Tissues. Bioengineering 2021, 8 (2), 27 10.3390/bioengineering8020027. 33672626 PMC7924193

[ref148] Wan Ali W. N. S.; Ahmad Tarmidzi N. A. A Rare Case of Contact Allergy towards Impression Compound Material. Eur. J. Dent 2021, 15 (04), 798–801. 10.1055/s-0041-1731584. 34384124 PMC8630957

[ref149] Hashemi A.; Ezati M.; Mohammadnejad J.; Houshmand B.; Faghihi S. Chitosan Coating of TiO2 Nanotube Arrays for Improved Metformin Release and Osteoblast Differentiation. Int. J. Nanomedicine 2020, 15, 4471–4481. 10.2147/IJN.S248927. 32606689 PMC7319596

[ref150] Van Den Bulcke A. I.; Bogdanov B.; De Rooze N.; Schacht E. H.; Cornelissen M.; Berghmans H. Structural and Rheological Properties of Methacrylamide Modified Gelatin Hydrogels. Biomacromolecules 2000, 1 (1), 31–38. 10.1021/bm990017d. 11709840

[ref151] Dienes J.; et al. Semisynthetic Hyaluronic Acid-Based Hydrogel Promotes Recovery of the Injured Tibialis Anterior Skeletal Muscle Form and Function. ACS Biomater Sci. Eng. 2021, 7 (4), 1587–1599. 10.1021/acsbiomaterials.0c01751. 33660968

[ref152] Berkovitch Y.; Seliktar D. Semi-synthetic hydrogel composition and stiffness regulate neuronal morphogenesis. Int. J. Pharm. 2017, 523 (2), 545–555. 10.1016/j.ijpharm.2016.11.032. 28449923

[ref153] Park S.; Park K. Engineered Polymeric Hydrogels for 3D Tissue Models. Polymers (Basel) 2016, 8 (1), 23 10.3390/polym8010023. 30979118 PMC6432530

[ref154] Ho T.-C.; et al. Hydrogels: Properties and Applications in Biomedicine. Molecules 2022, 27 (9), 2902 10.3390/molecules27092902. 35566251 PMC9104731

[ref155] Iizawa T.; Taketa H.; Maruta M.; Ishido T.; Gotoh T.; Sakohara S. Synthesis of porous poly(N-isopropylacrylamide) gel beads by sedimentation polymerization and their morphology. J. Appl. Polym. Sci. 2007, 104 (2), 842–850. 10.1002/app.25605.

[ref156] Cui L.; Jia J.; Guo Y.; Liu Y.; Zhu P. Preparation and characterization of IPN hydrogels composed of chitosan and gelatin cross-linked by genipin. Carbohydr. Polym. 2014, 99, 31–38. 10.1016/j.carbpol.2013.08.048. 24274476

[ref157] Liu Z.; Luo Y.; Zhang K. P(AAm-co-MAA) semi-IPN hybrid hydrogels in the presence of PANI and MWNTs-COOH: improved swelling behavior and mechanical properties. J. Biomater Sci. Polym. Ed 2008, 19 (11), 1503–1520. 10.1163/156856208786140373. 18973726

[ref158] Agrawal S. K.; Sanabria-DeLong N.; Tew G. N.; Bhatia S. R. Rheological characterization of biocompatible associative polymer hydrogels with crystalline and amorphous endblocks. J. Mater. Res. 2006, 21 (8), 2118–2125. 10.1557/jmr.2006.0261.

[ref159] Jin F.; et al. Impact of Entanglement on Folding of Semicrystalline Polymer during Crystallization. ACS Macro Lett. 2023, 12 (8), 1138–1143. 10.1021/acsmacrolett.3c00364. 37503873

[ref160] Shi S.; Xu T.; Wang D.; Oeser M. The Difference in Molecular Orientation and Interphase Structure of SiO2/Shape Memory Polyurethane in Original, Programmed and Recovered States during Shape Memory Process. Polymers (Basel) 2020, 12 (9), 1994 10.3390/polym12091994. 32887279 PMC7564273

[ref161] Zhang H.; Han D.; Yan Q.; Fortin D.; Xia H.; Zhao Y. Light-healable hard hydrogels through photothermally induced melting-crystallization phase transition. J. Mater. Chem. A 2014, 2 (33), 13373–13379. 10.1039/C4TA02463J.

[ref162] Kurt B.; Gulyuz U.; Demir D. D.; Okay O. High-strength semi-crystalline hydrogels with self-healing and shape memory functions. Eur. Polym. J. 2016, 81, 12–23. 10.1016/j.eurpolymj.2016.05.019.

[ref163] Wei D.; et al. Semicrystalline Hydrophobically Associated Hydrogels with Integrated High Performances. ACS Appl. Mater. Interfaces 2018, 10 (3), 2946–2956. 10.1021/acsami.7b15843. 29278483

[ref164] Bustamante-Torres M.; Romero-Fierro D.; Arcentales-Vera B.; Palomino K.; Magaña H.; Bucio E. Hydrogels Classification According to the Physical or Chemical Interactions and as Stimuli-Sensitive Materials. Gels 2021, 7 (4), 182 10.3390/gels7040182. 34842654 PMC8628675

[ref165] Tang S.; Zhao L.; Yuan J.; Chen Y.; Leng Y. Physical hydrogels based on natural polymers. In Hydrogels Based on Natural Polymers; Elsevier, 2020; pp 51–89. 10.1016/B978-0-12-816421-1.00003-3.

[ref166] Kang H.-S.; Park S.-H.; Lee Y.-G.; Son T.-I. Polyelectrolyte complex hydrogel composed of chitosan and poly(γ-glutamic acid) for biological application: Preparation, physical properties, and cytocompatibility. J. Appl. Polym. Sci. 2007, 103 (1), 386–394. 10.1002/app.24623.

[ref167] Huglin M. B.; Rego J. M. Thermodynamic properties of copolymeric hydrogels based on 2-hydroxyethyl methacrylate and a zwitterionic methacrylate. Colloid Polym. Sci. 1992, 270 (3), 234–242. 10.1007/BF00655475.

[ref168] Nuhn L.; et al. Size-Dependent Knockdown Potential of siRNA-Loaded Cationic Nanohydrogel Particles. Biomacromolecules 2014, 15 (11), 4111–4121. 10.1021/bm501148y. 25338185

[ref169] Hiratani T.; Kose O.; Hamad W. Y.; MacLachlan M. J. Stable and sensitive stimuli-responsive anisotropic hydrogels for sensing ionic strength and pressure. Mater. Horiz 2018, 5 (6), 1076–1081. 10.1039/C8MH00586A.

[ref170] Hawes C. S.; et al. A resilient and luminescent stimuli-responsive hydrogel from a heterotopic 1,8-naphthalimide-derived ligand. Chem. Commun. 2017, 53 (44), 5989–5992. 10.1039/C7CC03482B. 28513664

[ref171] Selegård R.; Aronsson C.; Brommesson C.; Dånmark S.; Aili D. Folding driven self-assembly of a stimuli-responsive peptide-hyaluronan hybrid hydrogel. Sci. Rep 2017, 7 (1), 7013 10.1038/s41598-017-06457-9. 28765593 PMC5539109

[ref172] Li Y.; Zhou C.; Xu L.; Yao F.; Cen L.; Fu G. D. Stimuli-responsive hydrogels prepared by simultaneous ‘click chemistry’ and metal-ligand coordination. RSC Adv. 2015, 5 (24), 18242–18251. 10.1039/C4RA11946K.

[ref173] Chander S.; Kulkarni G. T.; Dhiman N.; Kharkwal H. Protein-Based Nanohydrogels for Bioactive Delivery. Front Chem. 2021, 9, 1 10.3389/fchem.2021.573748. PMC8299995 34307293

[ref175] Ju Y.; Hu Y.; Yang P.; Xie X.; Fang B. Extracellular vesicle-loaded hydrogels for tissue repair and regeneration. Mater. Today Bio 2023, 18, 100522 10.1016/j.mtbio.2022.100522. PMC9803958 36593913

[ref177] Gan J.; Sun L.; Chen G.; Ma W.; Zhao Y.; Sun L. Mesenchymal Stem Cell Exosomes Encapsulated Oral Microcapsules for Acute Colitis Treatment. Adv. Healthc Mater. 2022, 11 (17), 2201105 10.1002/adhm.202201105. 35737997

[ref178] Feng Q. Dynamic Nanocomposite Microgel Assembly with Microporosity, Injectability, Tissue-Adhesion, and Sustained Drug Release Promotes Articular Cartilage Repair and Regeneration. Adv. Healthc Mater. 2022, 11 (8), 2102395 10.1002/adhm.202102395. 34874119

[ref179] Hao Y.; Zhang W.; Qin J.; Tan L.; Luo Y.; Chen H. Biological Cardiac Patch Based on Extracellular Vesicles and Extracellular Matrix for Regulating Injury-Related Microenvironment and Promoting Cardiac Tissue Recovery. ACS Appl. Bio Mater. 2022, 5 (11), 5218–5230. 10.1021/acsabm.2c00659. 36265007

[ref180] Yang Y.; et al. Recent advances in polysaccharide-based self-healing hydrogels for biomedical applications. Carbohydr. Polym. 2022, 283, 119161 10.1016/j.carbpol.2022.119161. 35153030

[ref181] Quan L.; Xin Y.; Wu X.; Ao Q. Mechanism of Self-Healing Hydrogels and Application in Tissue Engineering. Polymers (Basel) 2022, 14 (11), 2184 10.3390/polym14112184. 35683857 PMC9183126

[ref182] Yang P.; Ju Y.; Hu Y.; Xie X.; Fang B.; Lei L. Emerging 3D bioprinting applications in plastic surgery. Biomater Res. 2023, 27 (1), 1 10.1186/s40824-022-00338-7. 36597149 PMC9808966

[ref183] Chen J.; et al. Antibacterial adhesive self-healing hydrogels to promote diabetic wound healing. Acta Biomater 2022, 146, 119–130. 10.1016/j.actbio.2022.04.041. 35483628

[ref184] Ju Y.; Hu Y.; Yang P.; Xie X.; Fang B. Extracellular vesicle-loaded hydrogels for tissue repair and regeneration. Mater. Today Bio 2023, 18, 100522 10.1016/j.mtbio.2022.100522. PMC9803958 36593913

[ref185] Gil-Castell O.; Ontoria-Oviedo I.; Badia J. D.; Amaro-Prellezo E.; Sepúlveda P.; Ribes-Greus A. Conductive polycaprolactone/gelatin/polyaniline nanofibres as functional scaffolds for cardiac tissue regeneration. React. Funct Polym. 2022, 170, 105064 10.1016/j.reactfunctpolym.2021.105064.

[ref186] Liu H.; et al. An Electroconductive Hydrogel Scaffold with Injectability and Biodegradability to Manipulate Neural Stem Cells for Enhancing Spinal Cord Injury Repair. Biomacromolecules 2023, 24 (1), 86–97. 10.1021/acs.biomac.2c00920. 36512504

[ref187] Yang Q.; et al. Exosomes-loaded electroconductive nerve dressing for nerve regeneration and pain relief against diabetic peripheral nerve injury. Bioact Mater. 2023, 26, 194–215. 10.1016/j.bioactmat.2023.02.024. 36923267 PMC10008840

[ref188] Uddin M. S.; Ju J. Effect of crosslinking agents on drug distribution in chitosan hydrogel for targeted drug delivery to treat cancer. Journal of Polymer Research 2020, 27 (3), 81 10.1007/s10965-020-02059-8.

[ref189] Osswald C. R.; Kang-Mieler J. J. Controlled and Extended In Vitro Release of Bioactive Anti-Vascular Endothelial Growth Factors from a Microsphere-Hydrogel Drug Delivery System. Curr. Eye Res. 2016, 41 (9), 1216–1222. 10.3109/02713683.2015.1101140. 26764892

[ref190] Liu J.; Tian B.; Liu Y.; Wan J.-B. Cyclodextrin-Containing Hydrogels: A Review of Preparation Method, Drug Delivery, and Degradation Behavior. Int. J. Mol. Sci. 2021, 22 (24), 13516 10.3390/ijms222413516. 34948312 PMC8703588

[ref191] Boffito M. Hybrid Injectable Sol-Gel Systems Based on Thermo-Sensitive Polyurethane Hydrogels Carrying pH-Sensitive Mesoporous Silica Nanoparticles for the Controlled and Triggered Release of Therapeutic Agents. Front Bioeng Biotechnol 2020, 8, 1 10.3389/fbioe.2020.00384. 32509740 PMC7248334

[ref192] Jalababu R.; Rao K. S. V. K.; Rao B. S.; Reddy K. V. N. S. Dual responsive GG-g-PNPA/PIPAM based novel hydrogels for the controlled release of anti- cancer agent and their swelling and release kinetics. Journal of Polymer Research 2020, 27 (4), 83 10.1007/s10965-020-02061-0.

[ref193] Ovando-Medina V. M.; Reyes-Palacios G. A.; García-Montejano L. A.; Antonio-Carmona I. D.; Martínez-Gutiérrez H. Electroactive polyacrylamide/chitosan/polypyrrole hydrogel for captopril release controlled by electricity. J. Vinyl Addit. Technol. 2021, 27 (4), 679–690. 10.1002/vnl.21842.

[ref194] Pochan D. J.; Pakstis L.; Ozbas B.; Nowak A. P.; Deming T. J. SANS and Cryo-TEM Study of Self-Assembled Diblock Copolypeptide Hydrogels with Rich Nano- through Microscale Morphology. Macromolecules 2002, 35 (14), 5358–5360. 10.1021/ma025526d.

[ref195] Auriemma G.; Russo P.; Del Gaudio P.; García-González C. A.; Landín M.; Aquino R. P. Technologies and Formulation Design of Polysaccharide-Based Hydrogels for Drug Delivery. Molecules 2020, 25 (14), 3156 10.3390/molecules25143156. 32664256 PMC7397281

[ref196] Zhang Y. S.; Khademhosseini A. Advances in engineering hydrogels. Science (1979) 2017, 356 (6337), 3627 10.1126/science.aaf3627. PMC5841082 28473537

[ref197] Annabi N.; et al. 25th Anniversary Article: Rational Design and Applications of Hydrogels in Regenerative Medicine. Adv. Mater. 2014, 26 (1), 85–124. 10.1002/adma.201303233. 24741694 PMC3925010

[ref198] Vasanthan K. S.; Srinivasan V.; Pandita D. Extracellular matrix extraction techniques and applications in biomedical engineering. Regenerative Med. 2021, 16 (8), 775–802. 10.2217/rme-2021-0021. 34427104

[ref199] Wang R.; Wang Y.; Yang H.; Zhao C.; Pan J. Research progress of self-assembling peptide hydrogels in repairing cartilage defects. Front Mater. 2022, 9, 1 10.3389/fmats.2022.1022386.

[ref200] Liu X. Three-dimensional-printed collagen/chitosan/secretome derived from HUCMSCs scaffolds for efficient neural network reconstruction in canines with traumatic brain injury. Regen Biomater 2022, 9, rbac043 10.1093/rb/rbac043. 35855109 PMC9290528

[ref201] Ning L.; et al. 3D bioprinting of scaffolds with living Schwann cells for potential nerve tissue engineering applications. Biofabrication 2018, 10 (3), 035014 10.1088/1758-5090/aacd30. 29911990

[ref202] Nguyen L. T. B.; Hsu C.-C.; Ye H.; Cui Z. Development of an in situ injectable hydrogel containing hyaluronic acid for neural regeneration. Biomedical Materials 2020, 15 (5), 055005 10.1088/1748-605X/ab8c43. 32324167

[ref203] Li J.; et al. Dual-enzymatically cross-linked gelatin hydrogel promotes neural differentiation and neurotrophin secretion of bone marrow-derived mesenchymal stem cells for treatment of moderate traumatic brain injury. Int. J. Biol. Macromol. 2021, 187, 200–213. 10.1016/j.ijbiomac.2021.07.111. 34310990

[ref204] Finklea F. B.; Tian Y.; Kerscher P.; Seeto W. J.; Ellis M. E.; Lipke E. A. Engineered cardiac tissue microsphere production through direct differentiation of hydrogel-encapsulated human pluripotent stem cells. Biomaterials 2021, 274, 120818 10.1016/j.biomaterials.2021.120818. 34023620

[ref205] Long G.; et al. Engineering of injectable hydrogels associate with Adipose-Derived stem cells delivery for anti-cardiac hypertrophy agents. Drug Deliv 2021, 28 (1), 1334–1341. 10.1080/10717544.2021.1943060. 34180762 PMC8245104

[ref206] Kim K. S.; et al. Transplantation of 3D bio-printed cardiac mesh improves cardiac function and vessel formation via ANGPT1/Tie2 pathway in rats with acute myocardial infarction. Biofabrication 2021, 13 (4), 045014 10.1088/1758-5090/ac1e78. 34404035

[ref207] Hu Y.-F.; et al. Biomaterial-induced conversion of quiescent cardiomyocytes into pacemaker cells in rats. Nat. Biomed Eng. 2022, 6 (4), 421–434. 10.1038/s41551-021-00812-y. 34811487

[ref208] Ravi S.; Chokkakula L. P. P.; Giri P. S.; Korra G.; Dey S. R.; Rath S. N. 3D Bioprintable Hypoxia-Mimicking PEG-Based Nano Bioink for Cartilage Tissue Engineering. ACS Appl. Mater. Interfaces 2023, 15 (16), 19921–19936. 10.1021/acsami.3c00389. 37058130

[ref209] Yan J.; et al. Hydrogel-hydroxyapatite-monomeric collagen type-I scaffold with low-frequency electromagnetic field treatment enhances osteochondral repair in rabbits. Stem Cell Res. Ther 2021, 12 (1), 572 10.1186/s13287-021-02638-6. 34774092 PMC8590294

[ref210] Bordini E. A. F.; et al. Injectable Multifunctional Drug Delivery System for Hard Tissue Regeneration under Inflammatory Microenvironments. ACS Appl. Bio Mater. 2021, 4 (9), 6993–7006. 10.1021/acsabm.1c00620. PMC13037552 35006932

[ref211] Liu C.; et al. 3D Printed Gelatin/Sodium Alginate Hydrogel Scaffolds Doped with Nano-Attapulgite for Bone Tissue Repair. Int. J. Nanomedicine 2021, 16, 8417–8432. 10.2147/IJN.S339500. 35002236 PMC8722573

[ref212] Zhang H. 3D Printing Hydrogel Scaffolds with Nanohydroxyapatite Gradient to Effectively Repair Osteochondral Defects in Rats. Adv. Funct Mater. 2021, 31 (1), 2006697 10.1002/adfm.202006697.

[ref213] Ding X.; et al. 3D-Printed Porous Scaffolds of Hydrogels Modified with TGF-β1 Binding Peptides to Promote *In Vivo* Cartilage Regeneration and Animal Gait Restoration. ACS Appl. Mater. Interfaces 2022, 14 (14), 15982–15995. 10.1021/acsami.2c00761. 35363484

[ref214] Riau A. K.; Ong H. S.; Yam G. H. F.; Mehta J. S. Sustained Delivery System for Stem Cell-Derived Exosomes. Front Pharmacol 2019, 10, 1 10.3389/fphar.2019.01368. 31798457 PMC6868085

[ref215] Khayambashi P.; Iyer J.; Pillai S.; Upadhyay A.; Zhang Y.; Tran S. Hydrogel Encapsulation of Mesenchymal Stem Cells and Their Derived Exosomes for Tissue Engineering. Int. J. Mol. Sci. 2021, 22 (2), 684 10.3390/ijms22020684. 33445616 PMC7827932

[ref216] Khayambashi P.; Iyer J.; Pillai S.; Upadhyay A.; Zhang Y.; Tran S. Hydrogel Encapsulation of Mesenchymal Stem Cells and Their Derived Exosomes for Tissue Engineering. Int. J. Mol. Sci. 2021, 22 (2), 684 10.3390/ijms22020684. 33445616 PMC7827932

[ref217] Thomas V.; Yallapu M. M.; Sreedhar B.; Bajpai S. K. Breathing-in/breathing-out approach to preparing nanosilver-loaded hydrogels: Highly efficient antibacterial nanocomposites. J. Appl. Polym. Sci. 2009, 111 (2), 934–944. 10.1002/app.29018.

[ref218] Fan L. Exosomes-Loaded Electroconductive Hydrogel Synergistically Promotes Tissue Repair after Spinal Cord Injury via Immunoregulation and Enhancement of Myelinated Axon Growth. Advanced Science 2022, 9 (13), 2105586 10.1002/advs.202105586. 35253394 PMC9069372

[ref219] Gerlach J. Q.; Griffin M. D. Getting to know the extracellular vesicle glycome. Mol. Biosyst 2016, 12 (4), 1071–1081. 10.1039/C5MB00835B. 26888195

[ref220] Li M.; Ke Q.-F.; Tao S.-C.; Guo S.-C.; Rui B.-Y.; Guo Y.-P. Fabrication of hydroxyapatite/chitosan composite hydrogels loaded with exosomes derived from miR-126–3p overexpressed synovial mesenchymal stem cells for diabetic chronic wound healing. J. Mater. Chem. B 2016, 4 (42), 6830–6841. 10.1039/C6TB01560C. 32263577

[ref221] Henriques-Antunes H.; et al. The Kinetics of Small Extracellular Vesicle Delivery Impacts Skin Tissue Regeneration. ACS Nano 2019, 13 (8), 8694–8707. 10.1021/acsnano.9b00376. 31390518

[ref222] Vasco C.; et al. The Role of Adhesion Molecules and Extracellular Vesicles in an In Vitro Model of the Blood-Brain Barrier for Metastatic Disease. Cancers (Basel) 2023, 15 (11), 3045 10.3390/cancers15113045. 37297006 PMC10252721

[ref223] Hao D.; et al. Engineered extracellular vesicles with high collagen-binding affinity present superior *in situ* retention and therapeutic efficacy in tissue repair. Theranostics 2022, 12 (13), 6021–6037. 10.7150/thno.70448. 35966577 PMC9373818

[ref224] Li J.; Mooney D. J. Designing hydrogels for controlled drug delivery. Nat. Rev. Mater. 2016, 1 (12), 16071 10.1038/natrevmats.2016.71. 29657852 PMC5898614

[ref225] Siepmann J.; Siepmann F. Modeling of diffusion controlled drug delivery. J. Controlled Release 2012, 161 (2), 351–362. 10.1016/j.jconrel.2011.10.006. 22019555

[ref226] Xu L.; Liu Y.; Tang L.; Xiao H.; Yang Z.; Wang S. Preparation of Recombinant Human Collagen III Protein Hydrogels with Sustained Release of Extracellular Vesicles for Skin Wound Healing. Int. J. Mol. Sci. 2022, 23 (11), 6289 10.3390/ijms23116289. 35682968 PMC9181212

[ref227] Amsden B. Solute Diffusion within Hydrogels. Mechanisms and Models. Macromolecules 1998, 31 (23), 8382–8395. 10.1021/ma980765f.

[ref228] Huang C.-C.; et al. 3D Encapsulation and tethering of functionally engineered extracellular vesicles to hydrogels. Acta Biomater 2021, 126, 199–210. 10.1016/j.actbio.2021.03.030. 33741538 PMC8096714

[ref229] Ma Y.; Brocchini S.; Williams G. R. Extracellular vesicle-embedded materials. J. Controlled Release 2023, 361, 280–296. 10.1016/j.jconrel.2023.07.059. 37536545

[ref230] Henise J.; Yao B.; Ashley G. W.; Santi D. V. Autoclave sterilization of tetra-polyethylene glycol hydrogel biomaterials with β-eliminative crosslinks. Engineering Reports 2020, 2 (1), e12091 10.1002/eng2.12091.

[ref231] Wei C.; Song J.; Tan H. A paintable ophthalmic adhesive with customizable properties based on symmetrical/asymmetrical cross-linking. Biomater Sci. 2021, 9 (22), 7522–7533. 10.1039/D1BM01197A. 34643623

[ref232] Man K.; et al. Controlled Release of Epigenetically-Enhanced Extracellular Vesicles from a GelMA/Nanoclay Composite Hydrogel to Promote Bone Repair. Int. J. Mol. Sci. 2022, 23 (2), 832 10.3390/ijms23020832. 35055017 PMC8775793

[ref233] Yang S.; et al. MSC-derived sEV-loaded hyaluronan hydrogel promotes scarless skin healing by immunomodulation in a large skin wound model. Biomedical Materials 2022, 17 (3), 034104 10.1088/1748-605X/ac68bc. 35443238

[ref234] Toth M.; Fridman R. Assessment of Gelatinases (MMP-2 and MMP-9) by Gelatin Zymography,. In Metastasis Research Protocols; Humana Press; pp 163–174. 10.1385/1-59259-136-1:163. PMC3845455 21340898

[ref235] Tang J. Injection-Free Delivery of MSC-Derived Extracellular Vesicles for Myocardial Infarction Therapeutics. Adv. Healthc Mater. 2022, 11 (5), 2100312 10.1002/adhm.202100312. 34310068

[ref236] Wang C.; et al. Engineering Bioactive Self-Healing Antibacterial Exosomes Hydrogel for Promoting Chronic Diabetic Wound Healing and Complete Skin Regeneration. Theranostics 2019, 9 (1), 65–76. 10.7150/thno.29766. 30662554 PMC6332800

[ref237] Henriques-Antunes H.; et al. The Kinetics of Small Extracellular Vesicle Delivery Impacts Skin Tissue Regeneration. ACS Nano 2019, 13 (8), 8694–8707. 10.1021/acsnano.9b00376. 31390518

[ref238] Zhang Y.; et al. Umbilical Mesenchymal Stem Cell-Derived Exosome-Encapsulated Hydrogels Accelerate Bone Repair by Enhancing Angiogenesis. ACS Appl. Mater. Interfaces 2021, 13 (16), 18472–18487. 10.1021/acsami.0c22671. 33856781

[ref239] Han W. J.; Lee J. H.; Lee J.-K.; Choi H. J. Remote-controllable, tough, ultrastretchable, and magneto-sensitive nanocomposite hydrogels with homogeneous nanoparticle dispersion as biomedical actuators, and their tuned structure, properties, and performances. Compos B Eng. 2022, 236, 109802 10.1016/j.compositesb.2022.109802.

[ref240] Kubota T.; Kurashina Y.; Zhao J.; Ando K.; Onoe H. Ultrasound-triggered on-demand drug delivery using hydrogel microbeads with release enhancer. Mater. Des 2021, 203, 109580 10.1016/j.matdes.2021.109580.

[ref241] Kasiński A.; et al. Dual-Stimuli-Sensitive Smart Hydrogels Containing Magnetic Nanoparticles as Antitumor Local Drug Delivery Systems—Synthesis and Characterization. Int. J. Mol. Sci. 2023, 24 (8), 6906 10.3390/ijms24086906. 37108074 PMC10138940

[ref242] Zhou Y.; Liu G.; Guo S. Advances in ultrasound-responsive hydrogels for biomedical applications. J. Mater. Chem. B 2022, 10 (21), 3947–3958. 10.1039/D2TB00541G. 35593215

[ref243] Huebsch N.; et al. Ultrasound-triggered disruption and self-healing of reversibly cross-linked hydrogels for drug delivery and enhanced chemotherapy. Proc. Natl. Acad. Sci. U. S. A. 2014, 111 (27), 9762–9767. 10.1073/pnas.1405469111. 24961369 PMC4103344

[ref244] Zhou Y.; et al. Human adipose-derived mesenchymal stem cells-derived exosomes encapsulated in pluronic F127 hydrogel promote wound healing and regeneration. Stem Cell Res. Ther 2022, 13 (1), 407 10.1186/s13287-022-02980-3. 35941707 PMC9358082

[ref245] Wang Y.; et al. VH298-loaded extracellular vesicles released from gelatin methacryloyl hydrogel facilitate diabetic wound healing by HIF-1α-mediated enhancement of angiogenesis. Acta Biomater 2022, 147, 342–355. 10.1016/j.actbio.2022.05.018. 35580827

[ref246] Xu Y.; et al. miR-126–3p-loaded small extracellular vesicles secreted by urine-derived stem cells released from a phototriggered imine crosslink hydrogel could enhance vaginal epithelization after vaginoplasty. Stem Cell Res. Ther 2022, 13 (1), 331 10.1186/s13287-022-03003-x. 35870968 PMC9308191

[ref247] Razzauti A.; Lobo T.; Laurent P. Cilia-Derived Extracellular Vesicles in Caenorhabditis Elegans: In Vivo Imaging and Quantification of Extracellular Vesicle Release and Capture. Methods Mol. Biol. 2023, 2668, 277–299. 10.1007/978-1-0716-3203-1_19. 37140803

[ref248] Fan X.; et al. ‘Y’-shape armed amphiphilic star-like copolymers: design, synthesis and dual-responsive unimolecular micelle formation for controlled drug delivery. Polym. Chem. 2017, 8 (36), 5611–5620. 10.1039/C7PY00999B.

[ref249] Suwardi A. Machine Learning-Driven Biomaterials Evolution. Adv. Mater. 2022, 34 (1), 2102703 10.1002/adma.202102703. 34617632

[ref250] Li F.; et al. Design of self-assembly dipeptide hydrogels and machine learning via their chemical features. Proc. Natl. Acad. Sci. U. S. A. 2019, 116 (23), 11259–11264. 10.1073/pnas.1903376116. 31110004 PMC6561259

[ref251] Mei Y.; et al. Combinatorial development of biomaterials for clonal growth of human pluripotent stem cells. Nat. Mater. 2010, 9 (9), 768–778. 10.1038/nmat2812. 20729850 PMC3388774

[ref252] Yang J.; et al. Polymer surface functionalities that control human embryoid body cell adhesion revealed by high throughput surface characterization of combinatorial material microarrays. Biomaterials 2010, 31 (34), 8827–8838. 10.1016/j.biomaterials.2010.08.028. 20832108 PMC2992358

[ref253] Kwaria R. J.; Mondarte E. A. Q.; Tahara H.; Chang R.; Hayashi T. Data-Driven Prediction of Protein Adsorption on Self-Assembled Monolayers toward Material Screening and Design. ACS Biomater Sci. Eng. 2020, 6 (9), 4949–4956. 10.1021/acsbiomaterials.0c01008. 33455289

[ref254] Gubskaya A. V.; et al. Logical Analysis of Data in Structure-Activity Investigation of Polymeric Gene Delivery. Macromol. Theory Simul 2011, 20 (4), 275–285. 10.1002/mats.201000087. 25663794 PMC4316747

[ref255] Rostam H. M.; et al. Immune-Instructive Polymers Control Macrophage Phenotype and Modulate the Foreign Body Response In Vivo. Matter 2020, 2 (6), 1564–1581. 10.1016/j.matt.2020.03.018.

[ref256] Lee J.; Oh S. J.; An S. H.; Kim W.-D.; Kim S.-H. Machine learning-based design strategy for 3D printable bioink: elastic modulus and yield stress determine printability. Biofabrication 2020, 12 (3), 035018 10.1088/1758-5090/ab8707. 32252038

[ref257] Ruberu K.; et al. Coupling machine learning with 3D bioprinting to fast track optimisation of extrusion printing. Appl. Mater. Today 2021, 22, 100914 10.1016/j.apmt.2020.100914.

[ref258] Paxton N.; Smolan W.; Böck T.; Melchels F.; Groll J.; Jungst T. Proposal to assess printability of bioinks for extrusion-based bioprinting and evaluation of rheological properties governing bioprintability. Biofabrication 2017, 9 (4), 044107 10.1088/1758-5090/aa8dd8. 28930091

[ref259] Mas-Bargues C.; et al. Extracellular Vesicles from Healthy Cells Improves Cell Function and Stemness in Premature Senescent Stem Cells by miR-302b and HIF-1α Activation. Biomolecules 2020, 10 (6), 957 10.3390/biom10060957. 32630449 PMC7357081

[ref262] Bellmunt À. M.; López-Puerto L.; Lorente J.; Closa D. Involvement of extracellular vesicles in the macrophage-tumor cell communication in head and neck squamous cell carcinoma. PLoS One 2019, 14 (11), e0224710 10.1371/journal.pone.0224710. 31697737 PMC6837305

[ref263] Li L.; et al. Exosome-liposome hybrid nanoparticle codelivery of TP and miR497 conspicuously overcomes chemoresistant ovarian cancer. J. Nanobiotechnology 2022, 20 (1), 50 10.1186/s12951-022-01264-5. 35078498 PMC8787930

[ref264] Li Q.; et al. Genetically Engineered Artificial Exosome-Constructed Hydrogel for Ovarian Cancer Therapy. ACS Nano 2023, 17 (11), 10376–10392. 10.1021/acsnano.3c00804. 37194951

[ref266] Hu M. S.-M.; et al. The Role of Stem Cells During Scarless Skin Wound Healing. Adv. Wound Care (New Rochelle) 2014, 3 (4), 304–314. 10.1089/wound.2013.0471. 24761362 PMC3985511

[ref267] Jeon Y. K.; Jang Y. H.; Yoo D. R.; Kim S. N.; Lee S. K.; Nam M. J. Mesenchymal stem cells’ interaction with skin: Wound-healing effect on fibroblast cells and skin tissue. Wound Repair and Regeneration 2010, 18 (6), 655–661. 10.1111/j.1524-475X.2010.00636.x. 20955344

[ref268] Liang X.; Ding Y.; Zhang Y.; Tse H.-F.; Lian Q. Paracrine Mechanisms of Mesenchymal Stem Cell-Based Therapy: Current Status and Perspectives. Cell Transplant 2014, 23 (9), 1045–1059. 10.3727/096368913X667709. 23676629

[ref269] Shafei S.; et al. Exosome loaded alginate hydrogel promotes tissue regeneration in full-thickness skin wounds: An in vivo study. J. Biomed Mater. Res. A 2020, 108 (3), 545–556. 10.1002/jbm.a.36835. 31702867

[ref270] Maxson S.; Lopez E. A.; Yoo D.; Danilkovitch-Miagkova A.; LeRoux M. A. Concise Review: Role of Mesenchymal Stem Cells in Wound Repair. Stem Cells Transl Med. 2012, 1 (2), 142–149. 10.5966/sctm.2011-0018. 23197761 PMC3659685

[ref271] Ojeh N.; Pastar I.; Tomic-Canic M.; Stojadinovic O. Stem Cells in Skin Regeneration, Wound Healing, and Their Clinical Applications. Int. J. Mol. Sci. 2015, 16 (10), 25476–25501. 10.3390/ijms161025476. 26512657 PMC4632811

[ref272] Sinno H.; Prakash S. Complements and the Wound Healing Cascade: An Updated Review. Plast Surg Int. 2013, 2013, 1–7. 10.1155/2013/146764. PMC3741993 23984063

[ref273] Schwab A. Extracellular vesicles from infected cells: potential for direct pathogenesis. Front Microbiol 2015, 6, 1 10.3389/fmicb.2015.01132. 26539170 PMC4611157

[ref274] Fang S.; et al. Umbilical Cord-Derived Mesenchymal Stem Cell-Derived Exosomal MicroRNAs Suppress Myofibroblast Differentiation by Inhibiting the Transforming Growth Factor-β/SMAD2 Pathway During Wound Healing. Stem Cells Transl Med. 2016, 5 (10), 1425–1439. 10.5966/sctm.2015-0367. 27388239 PMC5031180

[ref275] Liang X.; Zhang L.; Wang S.; Han Q.; Zhao R. C. Exosomes secreted by mesenchymal stem cells promote endothelial cell angiogenesis by transferring miR-125a. J. Cell Sci. 2016, 129 (11), 2182–2189. 10.1242/jcs.170373. 27252357

[ref276] Jiang T.; Wang Z.; Sun J. Human bone marrow mesenchymal stem cell-derived exosomes stimulate cutaneous wound healing mediates through TGF-β/Smad signaling pathway. Stem Cell Res. Ther 2020, 11 (1), 198 10.1186/s13287-020-01723-6. 32448395 PMC7245763

[ref277] Hoang D. H. Differential Wound Healing Capacity of Mesenchymal Stem Cell-Derived Exosomes Originated From Bone Marrow, Adipose Tissue and Umbilical Cord Under Serum- and Xeno-Free Condition. Front Mol. Biosci 2020, 7, 1 10.3389/fmolb.2020.00119. 32671095 PMC7327117

[ref278] Zhang Y.; et al. Exosome/metformin-loaded self-healing conductive hydrogel rescues microvascular dysfunction and promotes chronic diabetic wound healing by inhibiting mitochondrial fission. Bioact Mater. 2023, 26, 323–336. 10.1016/j.bioactmat.2023.01.020. 36950152 PMC10027478

[ref279] Li Q.; et al. MiR146a-loaded engineered exosomes released from silk fibroin patch promote diabetic wound healing by targeting IRAK1. Signal Transduct Target Ther 2023, 8 (1), 62 10.1038/s41392-022-01263-w. 36775818 PMC9922687

[ref280] Jimi E.; Hirata S.; Osawa K.; Terashita M.; Kitamura C.; Fukushima H. The Current and Future Therapies of Bone Regeneration to Repair Bone Defects. Int. J. Dent 2012, 2012, 1–7. 10.1155/2012/148261. PMC3312195 22505894

[ref281] Exosomes derived from bone marrow mesenchymal stem cells improve osteoporosis through promoting osteoblast proliferation via MAPK pathway.

[ref282] Sun J.; et al. Engineering preparation and sustained delivery of bone functional exosomes-laden biodegradable hydrogel for in situ bone regeneration. Compos B Eng. 2023, 261, 110803 10.1016/j.compositesb.2023.110803.

[ref283] Zhang Y.; et al. Umbilical Mesenchymal Stem Cell-Derived Exosome-Encapsulated Hydrogels Accelerate Bone Repair by Enhancing Angiogenesis. ACS Appl. Mater. Interfaces 2021, 13 (16), 18472–18487. 10.1021/acsami.0c22671. 33856781

[ref284] Zhu Y.; Jia Y.; Wang Y.; Xu J.; Chai Y. Impaired Bone Regenerative Effect of Exosomes Derived from Bone Marrow Mesenchymal Stem Cells in Type 1 Diabetes. Stem Cells Transl Med. 2019, 8 (6), 593–605. 10.1002/sctm.18-0199. 30806487 PMC6525563

[ref285] Khazaei F.; Rezakhani L.; Alizadeh M.; Mahdavian E.; Khazaei M. Exosomes and exosome-loaded scaffolds: Characterization and application in modern regenerative medicine. Tissue Cell 2023, 80, 102007 10.1016/j.tice.2022.102007. 36577349

[ref286] Taylor D. A.; Sampaio L. C.; Gobin A. Building New Hearts: A Review of Trends in Cardiac Tissue Engineering. American Journal of Transplantation 2014, 14 (11), 2448–2459. 10.1111/ajt.12939. 25293671

[ref287] Liu B.; et al. Cardiac recovery via extended cell-free delivery of extracellular vesicles secreted by cardiomyocytes derived from induced pluripotent stem cells. Nat. Biomed Eng. 2018, 2 (5), 293–303. 10.1038/s41551-018-0229-7. 30271672 PMC6159913

[ref288] Zou Y.; et al. Restoring Cardiac Functions after Myocardial Infarction-Ischemia/Reperfusion via an Exosome Anchoring Conductive Hydrogel. ACS Appl. Mater. Interfaces 2021, 13 (48), 56892–56908. 10.1021/acsami.1c16481. 34823355

[ref289] Wang L. L.; et al. Sustained miRNA delivery from an injectable hydrogel promotes cardiomyocyte proliferation and functional regeneration after ischaemic injury. Nat. Biomed Eng. 2017, 1 (12), 983–992. 10.1038/s41551-017-0157-y. 29354322 PMC5773070

[ref290] Luo H.; et al. microRNA-423–3p exosomes derived from cardiac fibroblasts mediates the cardioprotective effects of ischaemic post-conditioning. Cardiovasc. Res. 2019, 115 (7), 1189–1204. 10.1093/cvr/cvy231. 30202848

[ref291] Wen Z.; et al. Mesenchymal stem cell-derived exosomes ameliorate cardiomyocyte apoptosis in hypoxic conditions through microRNA144 by targeting the PTEN/AKT pathway. Stem Cell Res. Ther 2020, 11 (1), 36 10.1186/s13287-020-1563-8. 31973741 PMC6979357

[ref292] Zheng H.; et al. Hemin enhances the cardioprotective effects of mesenchymal stem cell-derived exosomes against infarction via amelioration of cardiomyocyte senescence. J. Nanobiotechnology 2021, 19 (1), 332 10.1186/s12951-021-01077-y. 34674708 PMC8532335

[ref293] Santoso M. R. Exosomes From Induced Pluripotent Stem Cell-Derived Cardiomyocytes Promote Autophagy for Myocardial Repair. J. Am. Heart Assoc 2020, 9 (6), 1 10.1161/JAHA.119.014345. PMC7335524 32131688

[ref294] Heris R. M.; et al. The potential use of mesenchymal stem cells and their exosomes in Parkinson’s disease treatment. Stem Cell Res. Ther 2022, 13 (1), 371 10.1186/s13287-022-03050-4. 35902981 PMC9331055

[ref295] Yang C.; et al. Silk Fibroin Hydrogels Could Be Therapeutic Biomaterials for Neurological Diseases. Oxid Med. Cell Longev 2022, 2022, 1–12. 10.1155/2022/2076680. PMC9085322 35547640

[ref296] Deng Y.; et al. Exosomes derived from microRNA-138–5p-overexpressing bone marrow-derived mesenchymal stem cells confer neuroprotection to astrocytes following ischemic stroke via inhibition of LCN2. J. Biol. Eng. 2019, 13 (1), 71 10.1186/s13036-019-0193-0. 31485266 PMC6714399

[ref297] Xin H.; et al. MicroRNA-17–92 Cluster in Exosomes Enhance Neuroplasticity and Functional Recovery After Stroke in Rats. Stroke 2017, 48 (3), 747–753. 10.1161/STROKEAHA.116.015204. 28232590 PMC5330787

[ref298] Liu X. Hypoxia-pretreated mesenchymal stem cell-derived exosomes-loaded low-temperature extrusion 3D-printed implants for neural regeneration after traumatic brain injury in canines. Front Bioeng Biotechnol 2022, 10, 1 10.3389/fbioe.2022.1025138. PMC9562040 36246376

[ref299] Zhang Z.-W.; et al. Intravenous infusion of the exosomes derived from human umbilical cord mesenchymal stem cells enhance neurological recovery after traumatic brain injury via suppressing the NF-κB pathway. Open Life Sci. 2022, 17 (1), 189–201. 10.1515/biol-2022-0022. 35415238 PMC8932398

[ref300] Park J. Electrically Conductive Hydrogel Nerve Guidance Conduits for Peripheral Nerve Regeneration. Adv. Funct Mater. 2020, 30 (39), 1 10.1002/adfm.202003759.

[ref302] Cao J.-Y.; et al. Exosomal miR-125b-5p deriving from mesenchymal stem cells promotes tubular repair by suppression of p53 in ischemic acute kidney injury. Theranostics 2021, 11 (11), 5248–5266. 10.7150/thno.54550. 33859745 PMC8039965

[ref303] Cao J.; et al. Three-dimensional culture of MSCs produces exosomes with improved yield and enhanced therapeutic efficacy for cisplatin-induced acute kidney injury. Stem Cell Res. Ther 2020, 11 (1), 206 10.1186/s13287-020-01719-2. 32460853 PMC7251891

[ref304] Huang J. Mesenchymal Stem Cells-Derived Exosomes Ameliorate Ischemia/Reperfusion Induced Acute Kidney Injury in a Porcine Model. Front Cell Dev Biol. 2022, 10, 1 10.3389/fcell.2022.899869. PMC9171021 35686052

[ref305] Yang Z.; et al. 3D-Bioprinted Difunctional Scaffold for In Situ Cartilage Regeneration Based on Aptamer-Directed Cell Recruitment and Growth Factor-Enhanced Cell Chondrogenesis. ACS Appl. Mater. Interfaces 2021, 13 (20), 23369–23383. 10.1021/acsami.1c01844. 33979130

[ref306] Ma K.; et al. Articular chondrocyte-derived extracellular vesicles promote cartilage differentiation of human umbilical cord mesenchymal stem cells by activation of autophagy. J. Nanobiotechnology 2020, 18 (1), 163 10.1186/s12951-020-00708-0. 33167997 PMC7653755

[ref307] DiStefano T. J.; et al. Hydrogel-Embedded Poly(Lactic- *co* -Glycolic Acid) Microspheres for the Delivery of hMSC-Derived Exosomes to Promote Bioactive Annulus Fibrosus Repair. Cartilage 2022, 13 (3), 194760352211139 10.1177/19476035221113959. PMC9434687 36040157

[ref308] Lyu Y.; et al. Injectable Hyaluronic Acid Hydrogel Loaded with Functionalized Human Mesenchymal Stem Cell Aggregates for Repairing Infarcted Myocardium,. ACS Biomater Sci. Eng. 2020, 6 (12), 6926–6937. 10.1021/acsbiomaterials.0c01344. 33320638

[ref309] Heirani-Tabasi A.; et al. Cartilage tissue engineering by co-transplantation of chondrocyte extracellular vesicles and mesenchymal stem cells, entrapped in chitosan-hyaluronic acid hydrogel. Biomedical Materials 2021, 16 (5), 055003 10.1088/1748-605X/ac0cbf. 34144542

[ref310] Sang X.; et al. Thermosensitive Hydrogel Loaded with Primary Chondrocyte-Derived Exosomes Promotes Cartilage Repair by Regulating Macrophage Polarization in Osteoarthritis. Tissue Eng. Regen Med. 2022, 19 (3), 629–642. 10.1007/s13770-022-00437-5. 35435577 PMC9130414

[ref311] Wu X.; Crawford R.; Xiao Y.; Mao X.; Prasadam I. Osteoarthritic Subchondral Bone Release Exosomes That Promote Cartilage Degeneration. Cells 2021, 10 (2), 251 10.3390/cells10020251. 33525381 PMC7911822

[ref312] Fan W.-J. Exosomes in osteoarthritis: Updated insights on pathogenesis, diagnosis, and treatment. Front Cell Dev Biol. 2022, 10, 1 10.3389/fcell.2022.949690. PMC9362859 35959489

[ref313] Zhou Y.; et al. Exosomes derived from miR-126–3p-overexpressing synovial fibroblasts suppress chondrocyte inflammation and cartilage degradation in a rat model of osteoarthritis. Cell Death Discov 2021, 7 (1), 37 10.1038/s41420-021-00418-y. 33627637 PMC7904758

[ref314] Pishavar E.; et al. Advanced Hydrogels as Exosome Delivery Systems for Osteogenic Differentiation of MSCs: Application in Bone Regeneration. Int. J. Mol. Sci. 2021, 22 (12), 6203 10.3390/ijms22126203. 34201385 PMC8228022

[ref315] Chen M.; Luo D. A CRISPR Path to Cutting-Edge Materials. New England Journal of Medicine 2020, 382 (1), 85–88. 10.1056/NEJMcibr1911506. 31893521

[ref316] Montoya C.; Du Y.; Gianforcaro A. L.; Orrego S.; Yang M.; Lelkes P. I. On the road to smart biomaterials for bone research: definitions, concepts, advances, and outlook. Bone Res. 2021, 9 (1), 12 10.1038/s41413-020-00131-z. 33574225 PMC7878740

[ref317] Huang J.; Xiong J.; Yang L.; Zhang J.; Sun S.; Liang Y. Cell-free exosome-laden scaffolds for tissue repair. Nanoscale 2021, 13 (19), 8740–8750. 10.1039/D1NR01314A. 33969373

[ref318] Xu J.; et al. Injectable stem cell-laden supramolecular hydrogels enhance in situ osteochondral regeneration via the sustained co-delivery of hydrophilic and hydrophobic chondrogenic molecules. Biomaterials 2019, 210, 51–61. 10.1016/j.biomaterials.2019.04.031. 31075723

[ref319] Qiu H.; Liu S.; Wu K.; Zhao R.; Cao L.; Wang H. Prospective application of exosomes derived from adipose-derived stem cells in skin wound healing: A review. J. Cosmet Dermatol 2020, 19 (3), 574–581. 10.1111/jocd.13215. 31755172

[ref320] Safari B.; Aghazadeh M.; Davaran S.; Roshangar L. Exosome-loaded hydrogels: A new cell-free therapeutic approach for skin regeneration. Eur. J. Pharm. Biopharm. 2022, 171, 50–59. 10.1016/j.ejpb.2021.11.002. 34793943

[ref321] Zhang J.; et al. Exosome and Exosomal MicroRNA: Trafficking, Sorting, and Function. Genomics Proteomics Bioinformatics 2015, 13 (1), 17–24. 10.1016/j.gpb.2015.02.001. 25724326 PMC4411500

[ref322] Bari E.; et al. Pilot Production of Mesenchymal Stem/Stromal Freeze-Dried Secretome for Cell-Free Regenerative Nanomedicine: A Validated GMP-Compliant Process. Cells 2018, 7 (11), 190 10.3390/cells7110190. 30380806 PMC6262564

[ref323] Bruno S.; Chiabotto G.; Camussi G. Extracellular Vesicles: A Therapeutic Option for Liver Fibrosis. Int. J. Mol. Sci. 2020, 21 (12), 4255 10.3390/ijms21124255. 32549355 PMC7352992

